# Barriers and facilitators of care among visceral leishmaniasis patients following the implementation of a decentralized model in Turkana County, Kenya

**DOI:** 10.1371/journal.pgph.0004161

**Published:** 2025-03-31

**Authors:** Mariam Macharia, Collins Okoyo, Dawn Maranga, Jane Mbui

**Affiliations:** 1 Eastern and Southern Africa Center for International Parasite Control, Kenya Medical Research Institute, Nairobi, Kenya; 2 Department of Epidemiology, Statistics and Informatics, Kenya Medical Research Institute, Nairobi, Kenya; 3 NTD Programme, Foundation for Innovative New Diagnostics (FIND), Nairobi, Kenya; 4 Center for Clinical Research, Kenya Medical Research Institute, Nairobi, Kenya; Drugs for Neglected Diseases initiative, UNITED STATES OF AMERICA

## Abstract

Turkana County is among the eleven counties in Kenya endemic for Visceral Leishmaniasis (VL). Early diagnosis and management is crucial to improving quality of life, reducing related morbidity and mortality, and disease transmission. In Turkana County, Foundation for Innovative New Diagnostics (FIND) has undertaken significant work in supporting the decentralization of access to VL treatment. Despite this decentralization efforts, barriers to access care for VL still exist. This study sought to investigate the barriers and facilitators to VL care during the implementation of the decentralized model in Turkana County. A descriptive qualitative study was conducted in four health facilities within Turkana County. Data was collected using 13 in-depth interviews (IDIs) with VL patients/caregivers of children below 18 years, and 16 key informants’ interviews (KIIs) with health care workers. Data was audio recorded using a digital voice recorder, transcribed verbatim and analyzed using NVivo version 12. Due to low awareness of VL, majority of the patients did not directly associate VL symptoms to the disease. Barriers to seeking care were long distances to healthcare facilities, high transportation and other associated costs, and the initial use of traditional remedies. Facilitators for accessing care included referrals from diagnostic centers, medical camps and outreach programs, and support from community health promoters (CHPs). Notably, there was no reported stigma at either the individual or the community level among the VL patients. Despite patients awareness of the VL diagnosis and treatment centers, there was delay in seeking care due to inaccessibility of the centers. The delay is attributed to the large vast rural nature of the county, and the low socio-economic status of the affected communities. There is need for socio-economic support for the patients to access the treatment centers, and to provide comprehensive management to enable the attainment of the VL elimination goal in Kenya.

## 1.0 Introduction

Visceral Leishmaniasis (VL), also known as kala-azar, is a vector-borne disease caused by the protozoa *Leishmania* and transmitted by the bite of a sandfly [1–2]. VL ranks among parasitic infections worldwide with epidemic potential, thus a serious global public health concern [3–4]. However, due to extensive underreporting, the exact incidence rates remain unclear with new cases per year estimated to lie between 50,000 to 90,000 globally, with a case fatality rate exceeding 95% when left untreated [3,5]. Approximately, 60% of all VL cases worldwide occur in East Africa [3,6,7]. In Kenya, VL is endemic in 11 counties namely, Turkana, Baringo, West Pokot, Isiolo, Garissa, Wajir, Marsabit, Kajiado, Kitui, Tharaka Nithi, and Mandera with the estimated national case burden being around 2,000 cases annually [8].

In Kenya, VL is diagnosed using standard diagnosis algorithm as provided for by the Ministry of Health as contained in the VL diagnosis and case management guidelines of 2017 [9]. The algorithm includes clinical case definition in combination with the laboratory-based tests such as rK39 RDT, DAT, microscopy, culture, and molecular tests [9–12]. In the facilities where this study was done, VL was diagnosed using rK39 RDT. The guidelines provides that concomitant diseases and infections including TB, malaria, gastrointestinal infections, malnutrition, pneumonia, respiratory infections, skin infections, bacterial infections, among others be assessed as part of the diagnosis [9]. The standard treatment for VL in Kenya is a combination of Sodium StiboGluconate (SSG) and paromomycin administered for 17 days [9,13,14]. This same standard treatment was administered to majority of the patients attending the sample health facilities under study.

The World Health Organization (WHO) has earmarked VL for elimination in all endemic countries by the year 2030 by reducing the case fatality rate for primary VL to below 1%, and reducing the incidence of new cases to less than 1 case per 10,000 population [6,15,16]. This definition applies to all VL endemic countries in Africa [17–18]. However, the number of individuals suffering from VL globally has largely remained the same over the years, with only a slight reduction observed in the recent years [3,5]. Significant barriers to access to care constitute a major threat to the elimination efforts. Some of these barriers include delays in diagnosis and treatment, and persistent disease transmission, thus leading to increased morbidity and mortality [6]. In Kenya, VL diagnosis and treatment is free in the endemic areas, however, barriers to access care constitute a major challenge to disease control and elimination [8].

As a way to address these barriers, a decentralized model for VL management has been proposed [6,15]. A decentralized model would be characterized by the expansion of health facilities that can provide VL diagnosis and treatment to the affected communities. In Kenya, the Foundation for Innovative New Diagnostics (FIND) has undertaken significant work in supporting the decentralization process by expanding the number of health facilities offering VL diagnosis and treatment in Turkana County. In this county, they expanded the number of health facilities from six in 2018 to twenty-two in 2022. This study sought to investigate the barriers and facilitators to VL care during the implementation of the model in Turkana County.

## 2.0 Methods

### 2.1 Decentralization of visceral leishmaniasis care

Decentralization involves expanding the services related to VL diagnosis, treatment, and management from tertiary hospitals and central health facilities to local and peripheral health centers that are closer to the affected populations. The goal of decentralization is to improve access to timely and effective care, reduce delays in diagnosis and treatment, and contribute to sustainable VL control and elimination. The decentralization model described in this study was implemented in Turkana County through the support of FIND during the period 2018 to 2022. The overarching aim of this decentralization was to increase access to VL care for the underserved affected populations in the county. The success of this model in Turkana, has led to its implementation in Wajir and Marsabit counties in Kenya. This study will give empirical evidence for adoption of this model in other VL endemic countries.

### 2.2 Study design and sites

A descriptive qualitative study was conducted in four health facilities within Turkana County, Kenya. Turkana county was purposively selected due to its implementation of the decentralized model of VL care. The four health facilities within the county were purposively selected as they had VL patients under treatment during the data collection period. These facilities included, Lodwar County Referral Hospital (LCRH), Namoruputh PAG Health Center (NPHC), Lopiding Sub-County Hospital (LSCH), and Kakuma Sub-County Hospital (KSCH).

### 2.3 Study populations

In-depth interviews (IDIs) were conducted with all VL patients undergoing treatment and caregivers of minors (patients below 18 years) within the selected facilities. Further, key informants’ interviews were conducted with healthcare workers who were purposively selected from the same facilities which were treating the VL cases. The selected healthcare workers were directly involved in VL diagnosis and treatment and included clinicians, nurses, laboratory technologists and pharmacists. The selection process followed the laid down recruitment criteria that included screening of the participants to ensure that they meet the inclusion criteria and then subsequently consenting them to take part in the study. All the patients, the caregivers and the healthcare workers gave written informed consent.

### 2.4 Data collection

Data was collected between 27^th^ November and 15^th^ December 2023. The team trained four research assistants who were conversant with English, Kiswahili and the local language on study procedures and protection of human participants. IDIs and key informant interviews (KIIs) guides were used to moderate the interviews. The IDIs guide contained questions on participants knowledge, health-seeking behavior, barriers and facilitators to VL care and their perceptions. The KIIs guide contained questions on management of the disease, and the whole spectrum of VL care including treatment, reporting and stock management. Interviews were conducted in a quiet room within the health facility, and audio recorded using digital voice recorders. Patient interviews took approximately one hour while healthcare workers KIIs took 30 to 45 minutes. The interviews were conducted and stopped once the level of saturation was reached.

### 2.5 Data management and analysis

Patient interviews were conducted in Turkana language, while healthcare workers interviews were conducted in either English or Kiswahili depending on the participant choice. Data was transcribed verbatim, and the scripts translated to English. Back translation of the scripts was conducted by a different research assistant from the one who transcribed to ensure that the English transcript reflected the actual interview. Once this was established, the scripts were thoroughly read by the social scientist within the study team and organized in Microsoft word for manual analysis. Thematic areas were identified. The data was then entered into NVivo version 12 for further analysis. The analyzed data was then presented in text form.

### 2.6 Ethics statement

The ethical approval for this study was obtained from the AMREF Ethics and Scientific Review Committee (ESRC) under reference number P1476-2023. A research permit No. NACOSTI/P/23/25936 was obtained from the National Commission for Science, Technology, and Innovation (NACOSTI). Written informed consent was sought from all study participants before the start of the interviews and written permission to access the facilities sought from the heads of the respective health facilities and administrative approval from the County Department of Health.

## 3.0 Results

### 3.1 Socio-demographic characteristics of the participants

Thirteen (n=13) IDIs were conducted with patients and caregivers of minors (patients below 18 years) on treatment for VL from the four health facilities. The age range for the patients was between one and a half years and 30 years. Of these patients, 11 were children aged between one and a half years to 16 years, one was an emancipated minor aged 17 years, and one was an adult patient aged 30 years. As the minor patients could not participate in the interviews, eleven caregivers were interviewed. Over half (n=6) of the caregivers were female with majority having no formal education or employment (Table 1).

**Table 1:** Socio-demographic characteristics of IDI participants.

Characteristic	n=13	Percent (%)
**Gender of patients**		
Male	11	84.6
Female	2	15.4
**Age of patients in years**		
< 5	2	15.4
5-10	4	30.8
11-15	4	30.8
16-20	2	15.4
> 20	1	7.8
**Gender of caregiver**		
Male	5	45.5
Female	6	54.5
**Age of caregivers in years**		
< 25	2	18.2
25-35	4	36.4
36-45	4	36.4
> 45	1	9
**Education status of participants**		
No education	10	76.9
Primary incomplete	3	23.1
Primary complete	0	0
**Occupation of participants**		
Unemployed	6	46.2
Housewife	1	7.6
Farmer/pastoralist	6	46.2
Employed	0	0

A total of sixteen KIIs were conducted with the healthcare workers sampled from the four health facilities. There was an equal number of male (n=8) and female (n=8) participants. The age of the participants ranged between 27 to 46 years. All the participants had a tertiary level of education. The participants comprised of five nurses, four clinical officers, four pharmacists, and three laboratory technologists (Table 2).

**Table 2:** Socio-demographic characteristics of healthcare workers.

Characteristic	Frequency (n=16)	Percentage (%)
**Gender**		
Male	8	50.0
Female	8	50.0
**Age**		
20-29	4	25.0
30-39	9	56.3
40+	3	18.7
**Occupation**		
Clinical Officers	4	25.0
Pharmacists	4	25.0
Nurses	5	31.3
Laboratory Technologists	3	18.7

### 3.2 Participant knowledge on causes, transmission, and prevention of visceral leishmaniasis

Participants knowledge and awareness about VL were assessed using the validated qualitative guides. The participants were asked about the causes, symptoms, people at risk, transmission, severity, and prevention measures of VL.

The community refers to VL as “kala-azar” or “*Etid*” in their local language. The caregivers reported a distended abdomen as one of the symptoms their children were presenting with but unfortunately failed to associate it with VL. This was majorly due to the association of common VL symptoms to malaria, TB and liver disease. This led to delays in seeking medical care and subjecting patients to traditional forms of treatment.

*“Many people in Turkana also get kala-azar but it is always mistaken for the liver disease and that’s why they make some cuts in patients’ abdomen when you have the symptoms.”* (IDI_Caregiver)

*“The boy got sick with the symptoms like fever…mmh… so we thought it is malaria because we took him to a facility called Tulabalany where he received the medicines, but the condition persisted. So, we realized… eeh its different disease*.” (IDI_Caregiver)

The majority of the IDI participants learnt that their patient was suffering from VL at the health facility. The other reported sources of information on VL included community members, friends, neighbors, and through personal experiences.

*“The doctor was the one who tested, I was curious to know what he was suffering from. When I brought him to the government hospital, the doctor said he has kala-azar. I had no knowledge of what kind of disease this is.”* (IDI_Caregiver)

The cause of the disease was unknown to a majority of the IDI participants with only four associating causes directly to sandflies. Other reported causes included supernatural causes related to religion and witchcraft, drinking dirty water, and food related infection from eating fatty foods, raw meat, and drinking raw milk.

“*Sources of water from riverbanks and water from wells and pans…when they drink such water it creates a wound at the abdomen that causes enlargement of spleen…”* (IDI_Caregiver)

On transmission, IDI participants indicated that they were aware that VL could not be transmitted from one person to another. They therefore did not perceive themselves as a risk to others or other infected members of the family or community as a risk to them. This might have contributed to the absence of stigma within communities of Turkana County.

*“This disease is not spread from one person to another. When the person is infected, he/she will stay with it until it is treated...mmh…if the sandfly bites you, you are the only one who will be sick. You can even share food with the infected person, and you will not get it, you will not get the disease even by sitting near the infected person.”* (IDI_Caregiver)

On prevention, IDI participants indicated the need to break the existing anthills and discourage children from playing near anthills as ways of protecting themselves. Other prevention measures stated were seeking early care, improving the housing structures and use of mosquito nets.

*“For me I prefer destruction of anthills …mmh... also inventing the best medicines that may prevent those sandflies from coming out to bite people, and treatment of the anthills to be invented to flush out the sandflies.” (*IDI_Caregiver)

Data from both IDIs and KIIs appeared to indicate that children below 15 years and herders are at high risk of VL. Children were more susceptible due to their low immunity, poor hygiene practices, and social behavior of playing or resting on the anti-hills as they tend to livestock, which exposes them to bites of infected female phlebotomine sandflies.

*“…young children because their immune system is too weak...”* (IDI_Caregiver)

*“Children below the age of 15 years are prone to VL because of their immunity and also because they are mostly used as herders, they are the ones used to look after animals, so they are exposed to those areas where anthills are found. Thus, the risk of getting VL.*” (KII_HCW*)*

### 3.3 Health seeking behaviour for visceral leishmaniasis

Data shows that majority of the patients were aware that VL services were offered in the select facilities that they visited to seek treatment. The respondents were further aware of other facilities offering VL care both in Turkana County, neighboring Pokot County and in Uganda. This knowledge they indicated was mainly acquired from family, friends and other community members.

*“They seek medication at Kacheliba Hospital in West Pokot*…*it is only me that came to this side, other people seek treatment at Kacheliba.” (*IDI_Caregiver)

*“They seek treatment at this facility (NPHC) unless those that are living at far areas like Loreng’kipi who seek medication at Namudat Hospital in Uganda, they are assisted with transport by a local priest.”* (IDI_Patient)

### 3.4 Barriers in seeking care for visceral leishmaniasis

Despite awareness of IDI participants on where to seek care for VL, they did not visit the facilities immediately after symptoms developed. The wait period ranged between two weeks to several months with majority taking over one month before visiting the hospital. The participants indicated several challenges related to access to care as follows.

#### 3.4.1 Distance to the health facility.

The majority of the participants from both IDIs and KIIs indicated that patients reside in rural areas far away from the treatment centers. These areas are marred by poor road networks, lack of means of transport, and rough terrain. One of the participants indicated that he had to walk for over 3 days to access the facility which is 120 kilometers away from his home. Others indicated that the only form of transport available to them is motorbikes. Further, healthcare workers indicated that they were often forced to refer patients to facilities that have the capacity to offer blood transfusion services for severely anemic VL patients.

*“So in terms of any case that may come...be it a severe VL case we are fine, only that those cases that may require blood transfusion we are not able to offer such services from our end because we cannot transfuse blood from our place here, so we are supposed to send them to Lodwar. Even Lorugum, our Sub-County Hospital of late they are not offering that service. So, it becomes more strenuous to the patients when you are sending them far away for transfusion.”* (KII_HCW)

#### 3.4.2 Costs associated with visceral leishmaniasis treatment.

Despite VL treatment being free, there were other reported associated costs that patients had to incur to access care. These costs included transportation costs, costs on food, admission card costs, blood screening and transfusion, and costs for buying hematinic drugs (Table 3).

**Table 3:** Associated costs of treatment and indicated service spent on.

Participant ID	Amount Spent (In dollars)	Service spent on
001	N/A	No information
002	55	Food, medication and transport
003	15	Transport
004	38	Transport
005	23	Food and hospital bill
006	115	Food, drugs and nutritional support
007	N/A	No information
008	4	Admission card, malaria test and blood transfusion
009	12	tests
010	1	Admission card
011	4	Blood screening
012	62	Transport, food and blood transfusion
013	4	Diagnosis

*“I have sold three goats because there was no money. I spent almost $62. I used it to pay for blood transfusion, testing for kala-azar and for transportation through motorcycles. It is just that and some was used to buy some food for him to eat. I did not pay for doctor’s fee*.” (IDI_Caregiver)

#### 3.4.3 Use of traditional forms of treatments.

The use of traditional forms of treatment for VL patients was reported as a major barrier to early care within the communities. Majority of the IDI participants stated that they sought traditional treatments for VL before seeking medical care in the hospitals. These treatments were sought due to low awareness, community belief system that VL is treated by making incisions on the distended abdomen and allowing some blood to ooze out, a practice known as bloodletting, belief in supernatural causes, and advice from community traditional healers. The traditional forms of treatment included incisions on the abdomen and application of burnt camel and cow dung on the incision, visiting traditional healers, feeding the patient on blood from animals, and use of herbs among others.

*“…also before coming to the hospital, camel dung was put inside a fire and removed when it was too hot, it was administered to the incisions on the body until blood came out, this brought more pain in the body. Many cuts were done using the small knives.” (*IDI_Caregiver)

*“I will say aah’...they take maybe like two months...yeah … because you know for... they may try out some traditional interventions like aah’...they call it bloodletting where they incise the area around the spleen. They incise then some blood gets spilled over...so some even go to the extent of butchering a goat you know… those are traditional interventions. Those interventions do not work that’s when they come to the hospital...when the patients are usually weak...so it’s average of two months…yeah.” (*KII_HCW)

#### 3.4.4 Who makes the decision on health matters?.

The IDI respondents reported that adult males were the main decision makers in health seeking for the family. As the head of the household, the man made the decision on when and where to seek care, and financial decisions to meet associated costs of seeking VL treatment. This exclusive decision-making role by the adult male presents a challenge in early treatment for the patients, especially if the adult male is not knowledgeable about the disease. Additionally, being a pastoralist community, most adult males are not at home.

*“The husband is the one to decide if the patient will be taken for medication or not, he is the head of the family so his suggestion will be respected and followed. In the process of looking for transport to reach to the facility, husband again is the one to decide if the goat will be sold in order for them to get the money for transport for going to hospital, if he says no, it remains that way.*” (IDI_Caregiver)

#### 3.4.5 Failure to diagnose visceral leishmaniasis at initial hospital visit.

Several participants indicated that they had visited the local health facilities seeking medical care for the patients. The participants indicated that a blood sample was taken for testing. However, a positive VL diagnosis was not realized leading to most of them being put on malaria treatments. The participants indicated that even after being treated for malaria the symptoms persisted forcing them to visit other health facilities.

*“He started from Komen health center and they said it was malaria and we bought medicines from a chemist. No one tested blood to know if it was Kala-azar. They took blood sample for malaria only. He was treated for malaria, but the protruding abdomen did not heal.”* (IDI_Caregiver)

#### 3.4.6 Fear of loss of livelihood.

Some of the IDI respondents expressed fear of losing their source of livelihoods in terms of lost earnings, or death of their livestock during the time of treatment for VL. This fear was due to prolonged hospital stay and having no one to tend to their livestock.

“*For sure, doctor, our place is very far and at home we have livestock to keep so when we came here with my wife because of this boy’s condition. All those livestock and other animals might be lost or stranded because of our stay here…”* (IDI_Caregiver)

#### 3.4.7 Insecurity.

Data from KIIs indicated that insecurity within some areas contributed to delayed seeking of care for VL among communities of Turkana. This is because insecurity forced patients to wait for availability of government vehicles as a means of transport to foster their own personal security.

*“It forces them to look for a police vehicle coming to Lokichoggio so that they can use those vehicles to come to this sides because of security. So, it means somebody can stay at home for those 5 Months not being treated, 2 months, 3 Months like that.” (*KII_HCW)

#### 3.4.8 Lack of adequate awareness about visceral leishmaniasis as a disease.

Some of the IDI participants reported that the delay in seeking healthcare was occasioned by low awareness levels of VL among the community members. However, the healthcare workers noted that since decentralization, there has been an improvement in awareness levels at the patient level leading to reduced period of seeking medical attention.

*“Initially, the community did not have enough knowledge about VL. So, they used to perform some aah …” surgical” traditional procedures at home. Most patients in fact come with those traditional marks on their abdomen, as they thought maybe the spleen has enlarged so by cutting the abdomen you would be reducing the infection. But currently, with the awareness created for kala-azar, the health seeking behavior is on top. I said patients are coming as early as one month seeking for… even to be tested for kala-azar. If they feel the spleen is enlarged, they also relate it to VL.” (*KII_HCW)

#### 3.4.9 Lack of sufficient healthcare personnel within the facility.

Upon visiting the facilities, the IDI participants reported good experiences and health outcomes leading to improvement in general health and wellbeing and resumption of normal duty after initiation of treatments. However, the patients had a complaint about the health facilities having inadequate healthcare workers, especially nurses and doctors, leading to patients waiting long hours at the facility before being attended to. Further, healthcare workers indicated the need for adequate staffing within the facilities to reduce workers fatigue thus optimizing the provision of services.

*“More nurses should be brought to the facilities to avoid patients staying in hospital for a long time waiting for one nurse to attend to them, and who is also occupied with other duties.”* (IDI_Caregiver)

*“Also staffing I think the…the clinicians are not enough for example we have only one clinical officer. If that one clinical officer goes or is called to a meeting or other engagements at the county level, we remain without a clinical officer. Even the nurses are not that many…like now we are two.‘eeh’‘‘mmh’ so that is understaffing because we are few.”* (KII_HCW)

### 3.5 Facilitators of seeking of care of leishmaniasis cases

Factors that led patients to visit the health facilities included deteriorating health of the patient, fear of their child dying, and failure of traditional treatments. The choice of the health facility a patient visited was informed by personal belief that health facilities have trained health personnel, perceived good treatments, advice from friends and family members, referrals from VL diagnostic sites, referrals during community outreach programs, and referrals and financial support by the community health promoters (CHPs).

*“I chose to come to hospital because I knew that this is the best place where my child’s case will be settled and I believed that when I get to the facility, my child will bounce back strong due to medication.”* (IDI_Caregiver)

### 3.6 Perceptions towards visceral leishmaniasis

There was little or no expressed self or community stigma associated with VL. The IDI participants reported that community members regarded VL as a normal but deadly disease that required adequate medical attention. There was community support in terms of visiting the patients during admission, provision of monetary support, provision of food and prayers for the patients during the treatment duration.

*“...the community takes the VL patients like other ordinary people...they are not separating such kind of people in the community, so I have never seen any negative attitude towards them*…*”* (IDI_Patient)

## 4.0 Discussion

Access to improved diagnosis and treatment is among the proposed control strategies for VL towards WHO elimination goal 2030 [6,15]. In Kenya, efforts have been made by FIND to decentralize VL services by increasing the number of facilities that can diagnose and treat VL within Turkana County. This effort is aimed at reducing the time taken to seek diagnosis and to reduce VL related morbidity and mortality in the affected community. Despite this decentralization effort, challenges still remain in the access to VL treatment and care. This study assessed some of the underlying barriers and facilitators to access of VL care in Turkana County, Kenya.

This study observed that participant awareness about the decentralized treatment centers was relatively high. The high level of awareness of the existence of the treatment centers could be attributed to the implementation of decentralization process, a finding that has been supported by other studies in South Sudan [19]. However, they expressed the existence of barriers to access the centers. The barriers included long physical distance to the centers, insufficient funds to cover associated costs of transport and food, insecurity, use of traditional treatments, and fear of loss of livelihood during the treatment period. These barriers pose a significant threat to the control and elimination efforts of VL in the study area and thus need to be addressed. Some of the suggested ways to address these barriers by the participants included, continued decentralization of VL care so as to bring the services closer to the people affected by the disease, including VL patients in the national cash transfer program and improvement of security in the affected area.

Most of the participants in our study reported use of traditional forms of treatment for VL, a practice that appears to be a vital part of the VL treatment ecosystem in the county. This practice is however different from the neighboring Baringo County where studies have shown that VL patients seek medical treatment as opposed to traditional forms of treatment [20]. The use of traditional forms of treatments is mostly driven by community socio-cultural beliefs and practices. This calls for implementation of a targeted VL health education program within the affected communities to dispel the associated myths. In this community, as findings have shown, men play a critical role in influencing the health seeking behavior of the family members. Therefore, the VL health education program should also involve men for effective behaviour change in the community.

An additional barrier to early diagnosis is the inadequate health providers’ knowledge on VL symptoms. This study found that the health providers working at the primary level facilities in the county failed to suspect VL and only treated the patients for malaria. This finding is corroborated by studies conducted in Ethiopia that established the existence of gaps in healthcare provider knowledge on VL diagnosis [21]. Therefore, there is need to build capacity for healthcare workers within the VL endemic areas in diagnosis and treatment of the disease.

In this study, participants indicated personal belief that health facilities have trained health personnel, good treatments, advice from friends and family members, referrals from diagnostic sites for VL, referrals during community outreach programs, and referrals by the community health promoters and lack of stigma against the disease as key facilitators to seeking care and treatment for VL. The VL control and elimination program should target these facilitators as part of their programming package to the affected communities. These facilitators can be carefully coined and some delivered by the community health promoters, as part of their enhanced role in the primary healthcare delivery plan of improving social support and wellbeing at the community level.

### 4.1 Study limitations and strengths

The study had some limitations as follows: i) *The study design*: the study was not designed to assess impact of the decentralization model in removing barriers to VL care in a pre and post intervention design. We however conducted a cross-sectional descriptive study at the end of the model implementation to enable us get an in-depth understanding of the barriers and facilitators following a five-year period of implementation of the model in a rural community. The assumption here is that a successful implementation of this model would lead to fewer or no barriers to care. The established barriers and facilitators will be coined in a care package to refine the decentralization model in the same county or other endemic areas. ii) *Non-inclusion of healthy community members:* the non-inclusion of healthy community members who do not have VL, and opinion leaders in the community limited our ability to obtain the full perception of all community members in the study area. iii) *Absence of FGDs:* this study did not conduct any FGDs with the study participants thus limiting the full extent of the information obtained. The FGDs were not included in this study because the participants were patients who were receiving VL treatment, and healthcare workers who were involved in the treatment of these patients, and the day to day running of the facilities. Hence, it was not feasible to conduct an FGD with this group of participants. However, these limitations were overcome by obtaining first-hand experience of the VL patients through rigorous IDIs with a wide number of patients, and rigorous KIIs with the key healthcare workers attending to these patients. This is a key strength to this paper seeking to influence policy decisions for VL management in Kenya.

## 5.0 Conclusion

This study assessed key barriers and facilitators influencing the access to VL diagnosis and treatment in Turkana County, Kenya following the implementation of a decentralised model of care. These barriers need to be carefully addressed within the program implementation plan for successful elimination of VL in Kenya. The facilitators present strong promotive factors that should be enhanced within the program intervention package.

## Supporting information

S1 Appendix VL Patient In-depth Interview Guide. This document provides the guide used for conducting in-depth interviews with visceral leishmaniasis (VL) patients or their caregivers. It includes the questions and prompts designed to explore patient experiences, perceptions and barriers and facilitators to care.
(PDF)

S2 Appendix Knowledge, Attitudes, and Practices (KAP) Tool for Healthcare Workers.
This tool outlines the survey instrument used to assess the knowledge, attitudes, and practices of healthcare workers involved in VL care.
(PDF)

S1 Data Qualitative Transcripts. This file includes the following transcripts: •VL Patient In-depth Interview Transcripts: Verbatim transcripts of interviews conducted with VL patients, capturing their insights and lived experiences. •Healthcare Worker Key Informant Interview (KII) Transcripts: Transcripts from key informant interviews with healthcare workers, detailing their perspectives on decentralized care models for VL.
(ZIP)
