# Supplementary material for: Barriers and facilitators of care among visceral leishmaniasis patients following the implementation of a decentralized model in Turkana County, Kenya
Source: PLOS Glob Public Health. 2025 Mar 31;5(3):e0004161. doi: 10.1371/journal.pgph.0004161 (PMC11957299; doi:10.1371/journal.pgph.0004161)
Supplement: S1 Appendix — This document provides the guide used for conducting in-depth interviews with visceral leishmaniasis (VL) patients or their caregivers. It includes the questions and prompts designed to explore patient experiences, perceptions and barriers and facilitators to care. (PDF) [file pgph.0004161.s001.pdf]

Appendix 5.1: VL Patient In-depth interview guide

The survey of VL Patients will be carried out to understand the understanding of VL, Attitudes and perceptions, Knowledge and barriers to VL Treatment. *The survey will be delivered to patients diagnosed with VL.*

Interviewer name: (Please introduce yourself briefly)

Socio-Demographic Characteristics

1a) Sex (Male/Female)

b) Sex of child

2. Age in Years of patient (Exact age in years)

- Age of child in years

3. Marital Status (Enter one answer)

4. Religion (Enter one answer)

5. Level of Education (Enter one answer)

6. Main occupation (Enter one answer)

TURN ON THE RECORDER

QUESTIONS ON VL

KNOWLEDGE

1. How many days have you/your child been admitted at this facility?
2. Tell me about the condition for which you/your child are/is suffering from?
3. What do you think causes the disease you are suffering from? (Probe on causes)
4. Briefly describe some of the symptoms experienced by a person with the condition/kalazar
5. From where did you learn about the condition you/ your child is/are suffering from? (probe on source of information- is it family, friends, health facility, media etc)
6. Is there any other member of your household or community member you are aware of that has suffered a similar disease?
   - What symptoms did they have
   - Did they seek treatment- from where and after how long?
   - Was the condition treated successfully?
7. Do you think this condition is a problem within the village you come from?
8. Compared to malaria and other conditions, how would you describe VL burden in your area?
9. Whom do you think is most at risk of getting kalazar?
   -probe on persons
   -probe on areas where VL is more prevalent?
   -probe on when a person is most at risk
   -what factors within your environment increase risk of someone getting VL
10. Tell us more about the disease and how you think it is spread?
11. What do you think you can do to protect yourself and your child from the disease?

12. Briefly tell me how the disease is diagnosed
13. Briefly tell me how the disease is treated

HEALTH SEEKING BEHAVIOUR

14. When did you first become aware that you were ill?
15. What are some of the symptoms you experienced before coming to the facility?
16. What symptom made you feel the most need to visit the health facility?
17. For how long did you have the symptoms before visiting the facility?
18. What made you wait for (indicate number of days in 17 above) before seeking for treatment?
    -Please tell me any factors that prevented you from seeking help earlier for your illness
19. Did you seek an alternative source of treatment before coming to the facility? Or how did you deal with the symptoms before visiting the facility
    - Talk about what they were diagnosed on
    - Talk about treatments provided and whether they were effective
20. What are the challenges you experience as a Kala azar patient?
    - How much effort did getting VL care take?
21. What factors motivated you to seek help outside of your household for your illness?
    - What are your reason for choosing the hospital?
22. What measures if any helped you during your process of seeking care
23. Among your household, who decides on whether to seek or not seek care when a person gets sick?
24. Were you aware you could get diagnosis and treatment for Kala Azar in this facility before you fell ill? **(Yes or No)**
    b. If no to question 1, who informed you of this facility?
    c. If yes, where did you get this information? Who told you about it?
25. Where do your community members seek help for the condition you are suffering from?
    -what do people in your community say/believe about the place they seek care
26. Please tell me of your experience on the healthcare you are receiving
    - When you got to the hospital what tests were conducted on you for them to determine the condition?
    -how do you perceive the waiting time from receiving diagnoses to treatment
    -Please tell me about treatments that you are taking?
    -what is your opinion on how the treatment is administered? The taste, side effects etc
    - Do you feel that the medication you are being given will heal the condition?
    -do you have any concerns on the treatment and the care you received?
    -do you have any concerns on follow-up of care once you leave the facility?
27. What kind of support are you receiving from family and friends to help you cope with the long hospital stay and kalazar treatment? (Please tell us what you think of the support you have received).
28. How much does it cost you as a Kalazar patient, in terms of personal expenses? (An estimate is ok). Probe on What are the expenses for/what did you spend on e.g transport to hospital, meals, medication, doctor/nursing fees etc
29. In considering, the steps you took, what do you think you would do differently now if you could start from the beginning?
30. What changes/interventions would you suggest to improve VL care and access to VL Care?
    -to protect people from getting VL

-to improve diagnosis, care and treatment for those with VL

31. If any of your friends or relatives developed VL, what would you recommend to them in terms of treatment?
32. Are you aware of any past interventions for VL in the county?

BARRIERS TO ACCESS OF CARE

33. Kindly give more information about the barriers to access of VL diagnosis care and treatment (probe on the barrier that causes greatest problem)
34. Please tell me what type of people have the greatest challenge accessing VL Treatment and why?- age, gender, underlying conditions etc
35. What are the measures you feel should be put in place to address the barriers and improve access to VL services?-policy makers, healthcare authorities, NGOs etc.
36. What can you tell me about the risk of developing VL once a person leaves Turkana County and if you are aware of any available resources outside Turkana for VL Care

PERCEPTIONS OF VL

37. What do community members say about the condition you are suffering from?
    -And about people with the condition
38. What is the impact of community perceptions on VL care and diagnosis?
39. What can be done at the community level to reduce stigma?
40. What is the best way to involve the community in strategies to combat and control VL
