# Supplementary material for: Barriers and facilitators of care among visceral leishmaniasis patients following the implementation of a decentralized model in Turkana County, Kenya
Source: PLOS Glob Public Health. 2025 Mar 31;5(3):e0004161. doi: 10.1371/journal.pgph.0004161 (PMC11957299; doi:10.1371/journal.pgph.0004161)
Supplement: S2 Appendix — This tool outlines the survey instrument used to assess the knowledge, attitudes, and practices of healthcare workers involved in VL care. (PDF) [file pgph.0004161.s002.pdf]

KNOWLEDGE/ PERCEPTIONS AND PRACTICE TOOL FOR HEALTHCARE WORKERS

Interview Questions:

1. Knowledge of VL: Tell me about Visceral leishmaniasis in the area (probes)

   a) What causes the disease
   b) How is VL transmitted from one person to the other?
   c) Which category of individuals is most at risk of VL and why? Probe on disease burden
   d) What are the symptoms that patients with VL Present to the facility with?
   e) On average how long do VL patients in this area take before seeking treatment after developing symptoms? (Probe on the reasons e.g distance, cost of treatment, cultural beliefs, pastoralism etc
   f) How do you handle patients once they present to the facility with the indicated symptoms? - Probe on; diagnosis and treatment offered
   g) What treatment do you offer for VL within this facility?
      - ➢ Questions on how they currently conduct VL treatment.
      - ➢ Questions on how they currently follow up VL patients after treatment.
      - ➢ Questions on major drug toxicities
   h) Questions on how they currently conduct VL stock management.
   i) Questions on how they currently conduct VL data reporting.
   j) Has any member of the community succumbed to the disease? - Probe on mortality
   k) What part of VL Diagnosis, treatment is most challenging for you?
   l) What part of VL diagnosis, care and treatment is most enjoyable for you?

2. **Perception of preparedness for system adaptability**

   a. How prepared do you feel to handle the provision of VL services within this facility? Probe on what has made their work in provision of VL Care easier

   b. Are you concerned about work demands that may come with managing VL cases in your facility?

      - ✓ Question on willingness to perform VL screening at part of their work routine

      - ✓ Questions on willingness to perform VL diagnosis as part of their work routine

      - ✓ Questions on willingness to perform VL treatment as part of their work routine

      - ✓ Questions on willingness to perform VL stock management as part of their work routine

- ✓ Questions on willingness to perform VL data reporting as part of their work routine

c. Has managing VL cases in your facility in any way affected your work schedule or your wellbeing (probe: more stress due to more workload, more working hours etc)…Probe on challenges faced in VL care, diagnosis and management

d. Have received any specific training or skill development related to the provision of VL services? (Probes: can you please share your experience with it?

e. Have you received more resources e.g., personnel/equipment to help you manage VL cases following decentralization of VL care in the County?

f. Do you think that bringing Visceral leishmaniasis services to this clinic has in any way affected other services at the facility?

3. What does the community say about VL and what is the impact of such perceptions on on care seeking?
4. If we were to roll out VL Diagnosis, care and management programs to other health facilities, what areas would you recommend we improve?
5. Whom do you think should be trained at the community level to improve health seeking behaviour for VL patients?

Do you have any questions for us? Thank you for your time and consideration. End interview
