# Supplementary material for: Barriers and facilitators of care among visceral leishmaniasis patients following the implementation of a decentralized model in Turkana County, Kenya
Source: PLOS Glob Public Health. 2025 Mar 31;5(3):e0004161. doi: 10.1371/journal.pgph.0004161 (PMC11957299; doi:10.1371/journal.pgph.0004161)
Supplement: S1 Data — This file includes the following transcripts: •VL Patient In-depth Interview Transcripts: Verbatim transcripts of interviews conducted with VL patients, capturing their insights and lived experiences. •Healthcare Worker Key Informant Interview (KII) Transcripts: Transcripts from key informant interviews with healthcare workers, detailing their perspectives on decentralized care models for VL. (ZIP) [file pgph.0004161.s003.zip › HCW and IDI transcripts/healthcare workers/Res 001_FACILITY 1.docx]

VL DECENTRALISED STUDY

HEALTHCARE WORKER INTERVIEW

**INTERVIEW**

Q1.Tell me about what causes the disease or kalazar?(popping sound of central processing unit)

RES.Kalazar!!!..yeah.I think according to what I understand,,yes,,kalazar is... a disease that is caused by a parasite called leishmania and mostly we have a vector that’s the sandflies that carries the disease from..ah..an infected individual to other person so mostly its spread through a bite by a sandflies..yeah,,okay.

Qb.so how is this kalazar been transmitted from one person to the other ?

RES.is just that the way I have explained is through a bite by..aa.. an infected sandflies..mmh,,

Qc.so which category of individuals is mostly affected or risk of getting this disease or condition?....

RES: looking at kalazar I think one is the region where somebody stays because we have those areas that are known to be endemic for the disease and also..oh.in every other disease the immune system are..a very key thing in determining those who have are at risk..so the immune system of an individual can also..can also….. be a predisposing factor..yeah and so many other things..okay..mmh…..

Qd.so can you tell me symptoms of kalazar?..symptoms of kalazar..?

RES:….symptoms…fever is one of them..hepatosplenomegaly…..that is enlargement of the spleen and liver..(popping sound)..mmh mostly they have anemia…...that is dropping of blood level..okay..yeh,,aah,,there so many(laugh)..weight loss..

Int:yes..we have also..bleeding..

Res: bleeding tendencies..yeah

Qe.so on average how long do kalazar patients in this area take before seeking treatment after developing symptoms?

RES.I think that’s not definite..that will depend..(phones vibrating)on particular patients..we have people who with poor health seeking behavior..so we have some that will wait until the patients is malnourish,,,.actually the patients is..ee..wasted almost completely wasted with other complications that’s when they seek medical attention but for those who are bit enlightened they come early enough and may be when they are diagnosed and treatment initiated they..they…they get.. the treatment abit timely..so..on on the period that people take…I think from my experienced..yes..people quiet take some time before coming to facility. They come to the facility when things are really bad..mmh..okay

Int:Basing on distance and also transportation ?

RES I think for Turkana county they have tried to make sure this service is available in the various sub counties..so somebody doesn’t have to come from loima to get treatment in lodwar so mostly we have treatment sites in the various sub counties but you see we have those that believes in,, referrals hospitals we have people coming from as far as from….mmhh.. kibish to lodwar we have people coming from lokitaung to lodwar..yeh..but generally the treatment and diagnosis has been decentralized to the various sub counties..okay.yeah…

Qf.So how do you handle those patients once they present themselves to the facility…since i.. with the indicated symptoms?

RES: Since..eh..I just work in the lab I don’t..aaah.. handle the clinical bit but for us in the lab what normally happen once we get a request from a clinician that this is a patient that we have examined and we feel that symptoms correlates with to kalazar..we normally do a diagnosis in the lab..aa we..we.we..have various methods that we used in our facility,,we do a rapid diagnosis..we also do..a. DAT.,that’s direct agluconsion test but because of turn around time for direct agluconsion test we normally tends to inclined towards the RDT because the…the RDT turn around time is less than thirty minutes..okay..yeah..so it is aa the patients is able to get a timely results and eventually they get to.. start the medication immediately…okay..yeah

Qg.what treatment do you offer for kalazar within this facility..?

RES: ….i think that’s for the clinical team because that’s not what we do for us we only diagnosed and we forward to clinician to manage…okay..yeah…

Que: So briefly tell me on how they currently conduct VL stock management..?

RES:…..aah..for VL stock management I think for the lab we handled mostly the suppliers for diagnosis so those are RK39,..RDT kits that we used and also the DAT commodities that is direct aglucotinition test commodities..so..for the supplies we get mostly we get our VL supplies from aaa FIND that is an organization that supports us in diagnosis of leishmaniasis…….so on stock normally what happen when we receive our stock we update what we have receive in stock pads and what we receive is also used by various other facilities within the county so we normally have distribution list so we distribute to various testing sites yeah now since we have been we are like the hub for the supplies we monitor our stock level and when our levels run low we make timely requisition to the organization..that is the FIND so that atleast we don’t services interruption most of the time..yeah.

Que:….what about…..on what about on VL data reporting?

RE:…..on data reporting we do a… monthly report… for VL….. actually……. all the facilities that we have empowered to perform the VL testing we get reports from them so these reports are pooled in and keyed in to KHIS…

Int: KHIS?...

Res: That is….

Int: Kenya Health or what KHIS…..?

Res: KHIS is like aaahh….is a platform where we….. all the reports ….all reports that relate to health are keyed in …so that at the national level they are able to see like for example what Turkana does and what every other counties does …yeah…so that it can inform some of the decisions at the national and actually county level ..yeah

Que: so on which reporting tool ….is it…eeeh… for this condition?

Res: For the lab we normally do a …aaaah lab summary ….that is we do aahh…..a summary for all the tests that we do, the positivity rates and everything…so we used 706 to summarize all the tests…..mmmhh

Que: That is 7……summary sheets?

Res: 706 Summary forms

Q.has any member of the community succumbed to the disease in this facility?

RES: ….that one you can not get that answer from the lab because mostly after diagnosis sometimes we don’t know what happens…mmh yeah

Q.what part of VL diagnosis treatment is most challenging to you?

RES:…...part of VL diagnosis that’s challenging….. I think the only challenging bit that we sometimes have in relations to diagnosis is….is like sometimes last year we had…aaah stock out for the kits and this is something that was happening country wide so we had no option …..but atleast we reverted to the direct aglucotination test despite the long turn round time and at least we were able to get diagnosis though late diagnosis due to the long turn around time.

QL.in VL or visceral leishmaniasis diagnosis treatment which part do you enjoy mostly?..in care or treatment…

RES: .In diagnosis I can say…. I enjoy every other bit (laughing moment) because that’s what actually I do so I enjoy what I do..yeah..the whole of it.

Qm.so compared to malaria how would you rate the VL burden in the county?

RES.no I can say malaria is what we have in malaria…. is high than the VL though the VL is….… I think malaria is high….malaria is higher than VL.

QN.can you tell me on the relationship between the HIV and VL?

RES. Like I told you when we were starting some of the predisposing factors to having this disease is a weakened immune system and as we understand that when somebody get infected with HIV the immune system get compromise so such characters when they get the sandfly bite theypicl almost immediately they get infected by vl unlike the…. the immune competent individual..

2Qa.how prepared do you feel to handled the provision of VL services within this facility?

Res: I didn’t get your question?

Int: (repeats question)

RES.i think for us we are okay… since we cannot complain about the kits we get the kits timely enough for I think we are well prepared to handle the VL cases…. though the other aspects that sometimes brings in a challenge is the blood issue ….because when someone gets infected with Kalazar…or the leishmania……. they intend to have very low blood levels …so most of them before they get treatment they need blood transfusion and as you know that blood is one of the very rare commodity that you don’t buy anywhere you totally depend on volunteers who come in to donate blood so sometimes we have VL patients but we don’t have blood..such challenges…..but I think we always try our best

Qb.are you concerned about work demands that may come with managing VL cases in your facility?...based on part on willingness to perform VL screening as part of work routine…?

RES……..so I think for our facility …because you see within a day …. Aah…one day we get roughly ..not more than ten samples for VL and this is something that is within our means and we are able to handle it and ….on demand we are able to handle what we get…...the cases we receive we are able to perfectly handle….mmh

Q,on the part of diagnosis???

RES: .yeah….thats what I am…..been talking about because majorly what I do is diagnosis.

Q. Treatment is for??

Res: Treatment is for the …..thats for clinical bit…the nurses and clinical officers and the MOs

Q.also the stock management??

Res:…..no on stock management is departmental….so we have those commodities that are for diagnosis …yes we have those commodities that are for treatment …..so for us we manage stock that is related to diagnosis…so the .. that’s the kit that are used for testing. ….yes then the pharmacy and may be the nurses they control the medication that are used to for the treatment of the same disease…yeh …

Int: thank you.

Qc.has managing VL cases in your facility in any way affected your work schedule or your well being?

RES.NO…..(long pause)

Int: what about the challenges ???

RES: ..challenges as I earlier alluded…

Int:When doing diagnosis…?

Res: when doing diagnosis..the challenges we normally have is when may…… be when we have challenges with the supplies…(supplies)…yeah when sometimes like I told you earlier that like last year we had stock out for the kits throughout the country so..we..we..we.we…really struggled during that period so mostly those are some of the challenges we have….. may be if we can have constant supply of the kits for diagnosis we will be sorted…. then again we also need to find a way of training as many personnels as possible so that they are able to do microscopy….because microscophy is the standard for confirmation of VL treatment success so for you to confirm that this patient has recovered from VL you are supposed to perform microscopy but for our facility we have a challenge..challenges are one,we don’t have trained personnels to do splenic aspiration..yes..we don’t have personnel’s to do microscopy so we need training for the lab to perform the microscopy examination and also training for the clinician to perform splenic aspiration....yeah…..

Qd.have you received any specific training or skill development related to the provision of VL services?

RES.yes ..yeah for the lab we normally have trainings on how to do the rapid testing and also we have had trainings in the past on how to perform the direct aglucotination test..so the only bit that’s still missing is microscophy that have already talk about..okay..

Qe.have you received more resources like personnel, equipment to help you manage VL cases following decentralization of VL in the county?

RES.i think for us we have received some….we normally receive some items not major equipments…but some items for diagnosis that we normally receive from FIND …FIND I said is the organization that supports VL diagnosis and treatment services in the county actually not only in our county but in Kenya.

Qf.do you think that bringing visceral leishmaniasis services to this facilities has in any way affected other services???

RES: NO……,actually it has helped us to improve because sometimes you can manage a patients assuming it…..is aahhh malaria instead of having a proper diagnosis and getting the actual results so you can…managing malaria then when you get to a point where you able to do diagnosis for VL you find out that you are dealing with VL instead of what you have been treating…okay thank you..yeh..

Q3.what does the community say about VL and what is the impact of such perceptions on care seeking?

RES.i think we have…ee. two groups of people here …we have groups of people that….that believe in getting medication at the hospitals and we also have another group that believe in various traditional methods of treatment…yeah for those that who have coming to the hospital I think we normally have…very good outcomes but for those that go for the traditional ….eeehh…traditional..(medication)..the traditional medication they have ….. eventually they end up in the hospital when it is too late so some of them you would find somebody dying because of VL due to that delay of ee treatment…yeh..

Q4.if we were to roll out VL diagnosis,care and management programs to other health facilities what areas would you recommend we improve??

RES…..i think we need to maintain steady supply of commodities that’s the diagnostic reagents…..(diagnostic reagents),,anddd…th.ethethe medication..yes and also because you see like we say some of this guys they are…very wasted and atleast they need something like a supplement to to make them abit stronger as their get medication so we can also improve on provision of the supplements for VL cases that’s atleast …we have when somebody is undergoing treatment we give them some supplements it will go a long way in..in..in improving our outcomes…okay…yeah.

Q5.the last questions eeh…yes..whom do you think should be trained at the community level to improve health seeking behavior for VL patients??

RES: ..i think we have CHVS OR CHPS I think if..if those guys are properly trained on some of this things we will be able to pick the cases as early as possible so that atleast we should get some good outcomes…yeh…yeh..and will make sure that they will talk people about…atleast they would demystify some of this myth that some of the people have on traditional medicines and the modern kind of medicines so that people can weigh the two and get to understand why we really advocate for modern medicines…

Int: okay

Res: yeh.

Int: thank you so much for your cooperation for your time…

Res: you are actually welcome I think this is important because the reason as to why we come to the facilities or rather to..to work in a health facility is to make sure we get the best for our patients so….(yes) when we have some of this interviews (research) and such studies…I think it can go along way in helping us improve the quality of care that we provide to our patients…so I am as well grateful

Int:…thank..you so much

Res: Much welcome
