# Supplementary material for: Barriers and facilitators of care among visceral leishmaniasis patients following the implementation of a decentralized model in Turkana County, Kenya
Source: PLOS Glob Public Health. 2025 Mar 31;5(3):e0004161. doi: 10.1371/journal.pgph.0004161 (PMC11957299; doi:10.1371/journal.pgph.0004161)
Supplement: S1 Data — This file includes the following transcripts: •VL Patient In-depth Interview Transcripts: Verbatim transcripts of interviews conducted with VL patients, capturing their insights and lived experiences. •Healthcare Worker Key Informant Interview (KII) Transcripts: Transcripts from key informant interviews with healthcare workers, detailing their perspectives on decentralized care models for VL. (ZIP) [file pgph.0004161.s003.zip › HCW and IDI transcripts/healthcare workers/Res 002_FACILITY 2.docx]

VL DECENTRALISED STUDY

KEY INFORMANTS INTERVIEW

LOCATION: FACILITY 2

INTERVIEW

Q1. I want to ask you about the knowledge you have about VL….visceral leishmaniasis…..the kalazar
a)What causes kala Azar
RES:…(paper noise)…Kala Azar is caused by a sandfly 'mmmh '

b)How is VL transmitted ?
RES:'mmmh ' transmission...it's through a bite of a tsetsefly.

Que:How is it transmitted from one person to the other?

Res: kala Azar….kalazar is not a communicable disease.

c) Which category of individuals is most at risk of getting kalazar?

RES:'Category'. mmh ' let's say under five years...'whoozing sound by a fly'.

Que: Why do you think it’s the under five the most at risk?
Res: Under five mostly ,,...of the…...'eeeh' let's say it's because of the low immune and 'mmmmh…ehhhe ' the other thing is whenthey are mostly exposed especially when the parents spread their nets ,they are not on the nets they are mostly playing outside.

Que: and where do you think are the areas that are most at risk of contracting the disease?

Ans: 'risk areas'…….lets say bushy places ....eeeehn ...unconducive housing like those made of grass ''mmh'..let's say vulnerable families who can't even afford a net.and those who cannot build appropriate housing '':eeeh' conducive environment and now bushes are not cleared….,there is stagnant water there,there are alot of anthills…….

d)(paper rustling)..What are the symptoms that patients of VL present to the facility with?
RES:'mmh'they come with nosebleeding, persistent high temperatures of above 38 and 39, they come with headache,they come with..tummy big… protruded abdomen….Some of the organs become big that's why they come with protruded abdomen.They also have lost of weight and they also come with low Hb….they generally ….you see them very weak.


e)On average how long do Kalazar patients in this area take before seeking treatment after developing the symptoms?
RES:''aarh' ….how long do they take?,,,,,,,,most of don't like hospital and also the distance from their home to the facility because these people come from far and these kala Azar patients live in far places so they take almost a month as they treat the disease traditionally …..i don’t know what they do….and when they see that it's not helping that's when they come when they one month sick it even two months.Thay come with low hb.'mmh


f)How do you handle patients once they present to the facility with the indicated symptoms.
RES:so when they come and we access them,we take priority those with fever,we take the pressure. First thing after assessment, you take temperatures because mostly they come with high temperatures then you manage the fevers. If it comes down then as you call the lab tech to take the blood samples. You treat first the presenting symptoms those that can be treated that time, like you subside the fevers, mostly then, Kala Azar patients have malaria always then you treat for malaria. After treatment for malaria that's when you initiate the kala Azar treatment as you also focus on hb .''mmmh" "voices from the hospital"

g) So am going to ask about the treatment now, how you treat them , conduct them and how you follow the patients after treatment and about the toxicities of drugs.
RES:"mmh" so after diagnosing of kala Azar,if for example you ''aaa' have been confirmed of malaria ,you send and confirm for kala Azar and because they always have low hb,so you treat first Malaria ,you start for anti - malarials and martinicks. You can also give Ranferon and also if Ifas can help bring it down you put on Ifas .'ooh ' tat' you ,after,when  they present with the symptoms 'ooh' the samples that you send and the laboratory"check Napitek,BS for mps that is malaria ,hb level you also want to check the level of hb ,and then Kala Azar.Kalazar you use R.A.T or RK 39 and RK 28 that are with the RDT now.So you sent for malaria,hb,Kala Azar and also P.I.T.C,yess.,'ammmh' aaah' HIV test that is pitc .So after the lab results you will see there if it's confirmed malaria, confirmed kala Azar and anaemia you start the treatment of malaria, yeah.And  aaah' you also you manage anaemia at the same time.So you put on a  tinity that is Ranferon and Ifas that treat anaemia.Then you put on Al that is if the patient can take orals you give Al to take,but if the weak person ,he/she is vomiting put on I V Artesunate that is you are treating malaria.So after treating malaria,and you see the malaria symptoms are okay,then you treat,you start now treatimg Kala Azar.Fir kala Azar treatment,there is Ampysom,there is Pentostam and then there is Paramomycin.So Ampysom and all these things you do ,you calculate as the age of the patient that is you give dosage as per the age,''theee' the weight I mean.Yes the weight .And then the treatment goes for 17 days that is for Ampysom' aah' not Ampysom ,for Pentostam and Paramomycin it goes for 17 days but for Ampysom, 'ooh' not sure 'ooh' 5days not 10 days. "Conversing with a colleague" "eeh".


• How do you currently conduct VL treatment?
RES: To me can not answer that question.It is a question to be answered by a clinician.

• How do you coduct follow up for VL patients after treatment?

So after 17 days of completion of kala Azar treatment which Is the medication,'mmh' we usually give another 2 by the way these patients we advice them to stay around because the drugs are daily once aday once aday.So it's once a day and they come from far we advice to get a place to stay because we don't have enough to 'inaudible' we can't stay with them in the hospital because we don't have rooms that we can keep them also there is no food that we ,that will make them stay here.We tell them to look for relatives or a place to stay so long as they come for drugs in the hospital."eeeh" so after 17 days of completion of treatment,we give another one week,  'yeah' one week.when we give them one week ,one week after one week we check  hb cause we want to release with less hb of above 10.'mmmh'So after one week we check hb.When hb is above 10,then we discharge.As we discharge ,we give them another 6 months to go and stay at home.After 6 months,they come for another check up.

• How would you describe the toxicities of the drugs?
RES: "toxicities?So mostly Ampysom, 'mmmh' that patient starts to  itch ,he/ she starts itching and feel hotness ,and wants to urinate, that is Ampysom.

• What about stock management,how do you conduct stock management and data reporting?
 RES: I don't order,it's Steve that does the ordering and so he deals with ordering the drugs,making sure they are available. Steve is the acting pharm tech so he might know or he knows about the, but what am sure there i s''aaamm' the facility don't order or buy the kala Azar  what' medication.'eeh'.Those who were supporting the kala Azar patients are the ones who bring them.("Maybe I should ask ")

• What about data reporting how do you do that?
RES: 'Data reporting' it's the C.O that does data reporting.

j) Has any member of the community succumbed to the disease?
RES:'member of my community '..'around this place'.."whose" 'pardon' mmmh since we came no,no one,Yes no death due to kala Azar.

k) What part of kala Azar diagnosis, treatment is most challenging for you?
RES :I think it's care…..care because these people come from far places right ? and aah' they have no homes around,some even don't have relatives around and others are the ones chased from Uganda and they have never been ……"they are immigrants".So when you advice them to find somewhere close to the facility at least they be accessible to be near it becomes now to beg with them and it's not that they are refusing,they don't have the relatives around so keeping them at the facility there are no space for them,there are no house for them especially food,they have no food and these people they are supposed "..as long as" they ,eeh ..when you give drug they are also supposed to be supported nutritionally and these people with low hb and anaemia at least if they can get food to eat treatment too can pick.You find that you are giving medication,then the patient there. You found me  walking around with a girl child "mmmh..that child is not eating because the porridge she is given here has no sugar .It is a milled porridge made with maize,it has no sugar .Where she stays is not their home so she doesn't get food there.So you get the child is very weak that is why I was taking her to check hb .Hb is coming down before the , compared to the first one so that's another challenge in care.They don't have food.

l)What part of VL diagnosis,care and  treatment is most enjoyable to you?
RES:Let's say to diagnose ,you Know the signs and symptoms,you know the...when they are supposed to  ..the tests they are supposed to test.So you know the disages you want to give .you enjoy I mean the treatment,to diagnose and to prescribe.But in care .

m) Compared to malaria how would you rate the VL burden in the county?
RES: Malaria leads,malaria leads yeah than Kala Azar.

n)I need you to tell me the relationship between HIV and Kala Azar.
RES:"HIV and Kala Azar?" .."Trollies moved".. HIV lowers the immunity of a person same as to kala Azar.And also "eeh'' yeah HIV patients when they become sick ,they also run fevers the same as kala Azar ."mmh"

QUE 2
a)How prepared do you feel to handle the provision of VL services within this facility?
RES:'yeah ' we have the drugs for the patients for kala Azar that's why we are prepared,they are always available,so they are also available.'aaaah' also the clinicians are always there mmh.

b)What are your concerns about work demands that may come with managing kala Azar cases in your facility?
RES:Also staffing I think the, we are also,the clinicians were not that enough for example we have one C.O.Which that one clinical officer if he goes ,or get called to a meeting or other engagements at the county level,so we remain without a C.O like we are now.We have remained without a [C.O.so](http://c.o.so/) we manage.All the nurses are the ones that manage.Even the nurses are not that enough and now we are two.'eeh'''mmh' so that is staff, under- staffing because we are few.one clinical officer who is also  engaged at the county level.And the other is that we are running out of stock of drugs.'yeah' (not audible).

Drugs also is a problem 'mmh' pressure for food too ,you get to see a patient but you don't have that support nutritionally.So you get to see a patient you gave the drugs but you are not getting to manage. The hb is not raising because the patient is not supported nutritionally.

• How willing are you to perform the VL interms of screening as part of your work routine?
RES:we are always there 'yeah'.Am always there.I come in the morning,we usually make sure they get injected.''you find them not there'? They were already injected. So I remained with the child because she does not eat.

c)Has managing Kala Azar cases in your facility in any way affected your work schedule or your well-being?
RES:No.only the stress for patients food "hahaha"..its just for the food for these patients.''yeah'' because we confuse for them to live even with their relatives ,the result and food support ,so they eat and come take their medicines ,so they should just get a shade to sleep at least.Because there's also hunger outside there,and you can't accommodate someone as well as give them food.
We Don't go for trainings or this people I don't if they give but have never gone for Kala Azar training .I just use the knowledge I got from school. "Aah' that's the one I practice with in the facility.but we've not gone any kala Azar training.

d)Have you ever received any specific training or skill development related to the provision of VL services?
RES:No. Personally but am sure there are those that have went the C.O and the nurse incharge who is also not around.I think they have attended not all the staffs.

e)Have  you received more resources e.g personel/equipment to help you manage VL cases following decentralization of VL care in the county?
RES: There were those work aids that they kept there, that shows the signs  and symptoms, you manage ,how you prescribe.

f)Did you think that bringing visceral Leismaniasis services to this clinic has in any way affected other services at the facility?
RES:No.

QUE 3:What does the community say about kala Azar and what's the impact of such perceptions on care seeking?
RES : So 'aarh.  'they '..The community has their own beliefs,,they ,before they reach in the facility,they arrive already with cuts .They think it's a kidney disease'yeah' the cultural beliefs,what can I say,you get that they had already tried to treat themselves by cutting themselves.And then 'aah' under treatment,seeking the  treatment I think it only distance that hinder them ,you seeking that treatment.And also they know,they know .. how the kala Azar present .They know themselves.They even pulpate.So they just come.. eeeh .Mostly they come saying the patient himself says ''am sick kala Azar". So  you are like "how did you know?"so they know the signs, especially they pulpate the spleen when they see it is big they usually sure it's Kala Azar but now distance is what makes them take long but they know we provide kala Azar treatment.

QUE 4;If we were to rollout kala Azar diagnosis,care and management programs to other health facilities,what areas would you recommend we improve?
RES:''mmh"maybe I can, supply, supply those drugs and other things.Also they have to provide those who provide or diagnose or who access the patient.These trainings,they have to equip them on the diagnosis,signs , treatment and how different Kala Azar presents to the healthcare workers has to be equiped on how to diagnose Kala Azar.And also they have to provide this patients with food 'mmh'...the other thing is if possible if they can build Kala Azar site in one of the facilities providing kala Azar." Not even one but in any health facilities providing kala Zar .They should build a shade for them to complete their treatment because mostly ,the after, when they are injected this injections some aren't able to walk.so walking becomes hard ,we just encourage them .If they can find somewhere within the facility,where to stay after and until they complete treatment.

QUE 5: Whom do you think should be trained at the community level to improve health seeking behaviour for VL patients?
RES:'the''.Are they called [C.H.E.Ws](http://c.h.e.ws/) or what because they.The [C.H.E.Ws](http://c.h.e.ws/) ,the chiefs, and also these religious leaders because they usually have big numbers of people.so when they pass the information and the knowledge they always catch and big population.And so the C.H.E.W is the main person who is the community ,chiefs target their barazas.

Any question for me?
RES: maybe I don't have any question but my request to you,these have to be considered nutritionally,The patients.
