# Supplementary material for: Barriers and facilitators of care among visceral leishmaniasis patients following the implementation of a decentralized model in Turkana County, Kenya
Source: PLOS Glob Public Health. 2025 Mar 31;5(3):e0004161. doi: 10.1371/journal.pgph.0004161 (PMC11957299; doi:10.1371/journal.pgph.0004161)
Supplement: S1 Data — This file includes the following transcripts: •VL Patient In-depth Interview Transcripts: Verbatim transcripts of interviews conducted with VL patients, capturing their insights and lived experiences. •Healthcare Worker Key Informant Interview (KII) Transcripts: Transcripts from key informant interviews with healthcare workers, detailing their perspectives on decentralized care models for VL. (ZIP) [file pgph.0004161.s003.zip › HCW and IDI transcripts/healthcare workers/Res 003_ FACILITY 2.docx]

VL DECENTRALISED STUDY

HEALTH CARE WORKER INTERVIEW

FACILITY 2

**INTERVIEW**

Que: Tell more about VL in the area?

Res: Okay VL in the community is aahh… one of the diseases that are we are…fighting being among our top 10 diseases in our,, in our list of top 10 diseases

Que: yes

Res: So …it affects majorly people coming from the interior communities where they heavily reside in Bush and hilly areas and those are the heavily affected but aahhh its treatable….its preventable and it can be contained when early detected

Que: How Does it transmit from one person to the other?

Res: aaah…VL is transmitted through insect bite "eeh" insect bite, the insect,,, is called aafa sandfly.

Que: Yes

Res: So when…once the infected sandfly bites someone the person gets the disease.

Que: And what are the causes of the disease?

Res: The causes of the Kalazar or the..VL aaah,, results from the bite, from the sandfly. So once the infected sandfly bites you it leaves you with the parasites …you become ill and another sandfly bites you, it takes to the other person so thats how it is transmitted from one person to the other.

Que: So that is the main causes of it?

Res: yes

Que: Which category of individual is most at risk of this kind of disease?

Res: The risk population aahh….is one that resides especially in the bushy areas, the nomadic people or the nomadic community, The people who are not ah....eeh....clearing the Bush around their homes, the people who are living in the Bushy and hilly areas and also the areas which are heavily infested with the ant…or ants…. which built the anthills. So….Anthills also harbours the sandfly

Que: Is it burden to those peoples living in the bushes?

Res: Is it?

Que: Is it a burden to those people living in those areas?

Res: "yeah "..its… true it is a burden because maybe out of 10, the people come from those sides when they come to the facility, the primary diagnosis for them is always VL, so maybe 3 out of 10 they are negative but 7 are positive of VL. So it is a burden to them.

Que: Why the disease attacks Nomads mostly?

Res: Okay, they are the most affected because a,,, Sandflies are in bushy areas and this people looking for pasture and water,…. Pasture mostly found in Bushes, so its prone that those Sandflies at some points will bite people in the bushy looking for pasture because that is there area of residence.

Que: Oh okay

Res:"mmmh "

Que: What are the symptoms that the patients with VL present to the facility?

Res: 1. They come with complains of fever, 2. They come with complaints of headache, they also have enlarged spleen, some have enlarged "eeh" organs like liver, also some have complaints of nose bleeding and also aah….also some are pale , when look at them they are pale, showing the indication of low blood levels in their bodie.

Que: On average, How long do VL patients in this area take before seeking for treatment?

Res: due to the glaring challenges of….. of lack of transportation, poor infrastructure of roads , long distances aaahh….and this people living in interior part of this area. ideally 3 to 5 days someone take 1-2 weeks before arriving to seek the medication from the facility.

Que: Okay, and what are the cost of treatment?

Res: Cost, here is free. There is no amount of money that is involved when doing the ….when they are on treatment, the only treatment that are maybe something the cost might be card is…when there is this the drug called an…antihematic… drug called ranferon, which we give especially the low HB guys …it is sold 500 but the other IFAS and everything is free.

Que: How did the community take patients that are affected by the disease?..in terms of cultural beliefs

Res: …As far as VL is concerned there is no stigmatisation around it, they perceive it just like any other disease that is treated, they reside with this people, there is no side of sidelining or someone being stigmatised,,, so they take like…just like any other normal disease.

Que: How do you handle patients once they present to the facility with the indicated symptoms?

Res: aaah,,,we quickly do our clinical assessment, we send them to Lab, basing on the signs and symptoms presented, and once the Lab confirms that this is indeed Kalazar or the VL then we initiate treatment right away. Although…some come with Malaria and same time with VL so we treat Malaria first and now we initiate the VL treatment.

Que: What treatment do you offer for VL?

Res: Okay here we have the SSG paranomycin, SSG sodium stibogluconate and the paranomiycin We also offer veritam B or ambisom injection.

Que: And how do the patient conduct on treatment?

Res: Come up again?

Que: How do they currently conduct on treatment?

Res: the patients?

Que: No, you as the health worker.

Res: Okay,,,to be honest I request to come up with the question again.

Que: How do you conduct treatment?

Res: The treatment is here is done on daily basis, depending on the arrangement that someone has prescribed on, Done on Morning, every morning so like for SSG paranomycin we do it for 17 days and for ambisom we do it ,,,eeeh,, on alternate days, if someone is given today we skip a day or… tomorrow and "eeh"we do another dose like alternate days for 7 doses, that is how we conduct our care and treatment.

Que: Do you usually follow up patients after treatment?

Res: Yes we do, whereby we check the HB, we check the weight, we check the vitals and if someone is deteriorating we switch him or her to the second line if she was on first line we switch her to the second line and continue monitoring him or her, then if they are okay we give them a DSA or a next date of visiting 6 Months follow up and now we completely close their file if they are fully cured.

Que: Is there any toxicities on drugs

Res: yeah…There is been one case though some times back around 2019-2020, but since then we have not heard any case all our patients are responding well to the treatment, no report of any adverse drug reaction.

Que: How do you conduct on Stock management for VL?

Res: We have the stock cards which are available at our point of service delivery, we take "eeeh " The commodities out of the cabinets which are lockable we suppress them to the stock card and at end of the month we do physical count, we request for the new stock , so we have our routine our every day stock cards updating that we do on daily basis.

Que: How about data reporting, how do you conduct it?

Res: Data reporting, is done on monthly basis a,,, Where our record person takes the,,,, especially those who have been treated and finished their treatment. There is a system W.H.O one where she put the data there on behalf of the sub county HIRO. She also do monthly reporting on our workloads on the number of patients that we have treated and the number of patients that were sent to the lab and turn positive and we also review some of the cases in our monthly data review meetings.

Que: Has any member of the community succumbed to the disease?

Res: "yeah" Have heard the case though sometimes back, a Child died but it was due to negligence of the parents. He refused to take the child for transfusion in Lodwar, so and the child was having low HB of aaah… 3 and the child succumbed. And so the other day we heard a case where due to cultural things that happened around the VL context… the child was stubbed in thempiercing….the stubbing wound was so deep and actually the child succumbed.

Que: Which part of VL Diagnosis, treatment is most challenging for you?

Res: a,, On our part the most challenging part is maybe when the time of a,,, out of stock commodity, especially the RK 39 which is used for primary diagnosis and when it is out of stock we have to take the samples to Lodwar for dat or DAT. So during that period sometimes it might take long because from here to Lodwar is also a ….quite a long distance for that become so challenging at our side. And maybe when we are out of stock some of the commodities… like this year we had some crises on to do the SSG paranomymic commodities although now we are well stocked.

Que: oh Okay. And Which part of it do you usually enjoy on VL? While treating VL patients which part do you usually enjoy?

Res: The enjoying bit is always when we are seeing our patients "eeeh" getting the right diagnosis one, and 2. Getting the right treatment and they are improving because Some of them they come here very sick , and by day 3-4 on treated they are now fully…they are now on recovery mood that is the joy. When we see our VL patients getting back on their feet.

Que: Compared to Malaria, how would you rate VL burden in the facility, community or at the County? "Nurse talking "

Res: VL burden here is…as a facility we are narrowed down to the community that are in interior and and hilly side of our side, So out of 10, that come with Malaria will get around 4 with Kalazar. So 4 ratio 4/10 . So it's quite high especially on those sides but to the community surrounding the facility here this centre we Don't have cases. The burden is quite high to the communities presiding in the bush areas that you see we treat here as a daily basis

Que: Okay, and How do VL relate with HIV?

Res: VL HIV relationship aah…... There is none that have come across in out treatment experience so I cannot say much about that. But rather let me comment…. VL with Malaria,,, yes, VL Malnutrition,,,Yes. There is a big if co- relationship there that we have seen and we have treated them.

Que: How prepared do you feel to handle the provision of VL services within this facility?

Res: How prepared?

Que: Do you feel to handle the provision of VL services within the facility?

Res: aah….We are okay, we are well equipped like right now…. So in terms of any case that may come ..be it a severe VL case we are fine, only that those cases that may require blood transfusion we are not been able to offer such services from mour end because we don't transfused blood from our place here, so we are supposed to send them to Lodwar even Lorugum our Sub-County Hospital of late they are not offer that service, So it becomes more strenuous to the patients when you are sending them far away for transfusion

Que: Are you concerned about work demands that may come with managing VL cases in your facility?

Res: aaahhh…No because, we are used to it, we are okay and it is part of our job

VL: Okay. So you are willing to perform VL screening as part of working routine..?

Res: yea h we do…yes we do. That is what we always do it is like part of our activities, they are part of our workloads so any form of screening is always done and we always continue to be done.

Que: Has managing VL cases in your facility in any way affected your work schedule?

Res: Not at all.

Que: your wellbeing?

Res: Not at all, because it part of work and it is part of our activity

Que: What are the challenges that you faced in VL care ?

Res: come up again.

Que: Challenges you faced on VL care

Res: The challenges I think have highlighted some…sometimes back there that a 1. Maybe sometimes the supply of the commodities is erratic is not continuous, we are out of stock, like today we cannot do RK 39 test because we are out stock, a,,, also the transfusion services we don't do them here, and most of this people have low HB that require transfusion so that is a big challenge and lastly the nutrition component is never addressed by the donor or partners who are funding the program yes…though we gets some milk supplements but that is not enough for breakfast lunch and supper "child crying " so it is a quite challenging.

Que: Have you received any specific training related to provision of VL services?

Res: "yeah" we have though my colleagues have, in personal attended such training while I was away but the feedback is here with us and we are well updated.

Que: Will you mind to share your experience?

Res: They want to do,,, something do with the RK 28 and RK 39, DAT the Laboratory personnel went for that also there was something to do with VL management of this patients, the updated guide line so we are well updated, we did our CME here and everyone is having the update information as far as those trainings are concerned.

Que: Following decentralization of VL in the county, how have you received any equipment to help you manage VL cases?

Res: On equipment …not much but I can must say that I if I may categorise the kits as equipment as received to the RK 39 we received them, we received the research ones the RK 28 we received them for research purposes and we also received some equipment for transportation of samples to the Lab also DAT for further analysis so we received.

Que: Do you think that bringing VL services to this facility has in any way affected other services at the facility?

Res: Not at all. If anything it has improved our level of care and our level of service to our patients. Because we have a case here referring to the Lorugum or Lodwar it could mean that it could lose more patients but now we treat them here, we managed everything here so we save life's because we are offering such services

Que: How does the community say about VL?

Res: The community perspectives,,, about,,,on VL they take it just like any other disease

Que:yes

Res: No much stigma about it, no much stigmatisation about it. Cause they bring them here no one is kept at the community as much as they tried their tradition methods and and fail they will bring them here no one is hidden at the community level that can not be seen by the doctors here.

Que: If were to roll out VL diagnosis, care and management programs to other health facilities, what areas would you recommend we improve?

Res: Improve on the supply of the commodities, 1.the RK39 improve on …that they should always be there. 2. Improve on the supply of medicines or the medications so that there is no shortage of drugs….should also be there when someone is diagnosed. He or she initiated with treatment. 3. Aa,,, at least have one blood transfusion site decentralised from Lodwar to some of this facilities around so that we shorten the distance of going for transfusion to Lodwar, lastly Provide basic storage facilities for some of this drugs on VL like carpheritism B requires a fridge, requires power in that facility so it as to be decentralised to other facilities some of this facilities will lack this things, so bring them closer to them so that people can get the all diagnostic care that is required.

Que: Whom do you think should be trained at the community level to improve health seeking behaviour for VL patients?

Res: The VL people that got the disease, treated and cured. Those should be the VL champions at the community level; just like we have the cancer champions out there they should be the ones telling the community about the VL and create awareness about it

Que: We have come to the end of our interview,, do you have any question?

Res: Not much, not a question but maybe ah,,, maybe a compliment to say thank for the interview, Thank you for the session. We hope that the challenges we face will be well addressed and we hope for a positive feedback out of it, we should not just end here. We want to see a great improvement from the areas where we are need so that our people or our patients can get the quality care that they deserve and also reduce straining them where necessary so that at end of the Day they are better and they go back on their normal businesses as usual

Que:Okay

Res:"mmmmh"
