# Supplementary material for: Barriers and facilitators of care among visceral leishmaniasis patients following the implementation of a decentralized model in Turkana County, Kenya
Source: PLOS Glob Public Health. 2025 Mar 31;5(3):e0004161. doi: 10.1371/journal.pgph.0004161 (PMC11957299; doi:10.1371/journal.pgph.0004161)
Supplement: S1 Data — This file includes the following transcripts: •VL Patient In-depth Interview Transcripts: Verbatim transcripts of interviews conducted with VL patients, capturing their insights and lived experiences. •Healthcare Worker Key Informant Interview (KII) Transcripts: Transcripts from key informant interviews with healthcare workers, detailing their perspectives on decentralized care models for VL. (ZIP) [file pgph.0004161.s003.zip › HCW and IDI transcripts/healthcare workers/Res 004_FACILITY 2.docx]

VL DECENTRALISED STUDY

KII INTERVIEWS WITH HEALTHCARE WORKERS

SOCIO -DEMOGRAPHIC CHARACTERISTICS


INTERVIEW

Q1. I want to ask you about the knowledge you have about VL….the kalazar

a)What causes kala Azar?
RES :"" mmmh" sandfly 'mmh'

      b)How is Kalazar transmitted from one person to the other?
      RES :It is through a bite of a sandfly,'eeh'
     
      c)Which category of individuals is most at risk of getting kala Azar?and why?
      RES: The herdsmen and children

que: why do you think its herdsmen and children?

Res: Because they are the ones who are found in bushy areas and area that,that eeh anthill are found so they like grazing their animals near those areas and sometimes they sleep under the trees like the backs of the trees are open and you find that these sandfly normally rest 'eeh ' between the,, between the backs of the trees or it comes out from the anthill.
     
      d)What are the symptoms that petients with kala Azar present to the facility with ?
      RES:Most of them come with the,'mmh' complaining of fever, 'mmh' some of them have big belly or extruded belly and some of them are very weak. Some of them complain of they lack appetite' they lack appetite'.

       e)On average how long do kala Azar in your area take before seeking treatment after developing symptoms?
       RES:Most of them take even around one month, after a month is when they come to the facility.

Que: what are the reasons?
Res: Reason 1:One is transportation;they stay in far areas.two…. Lack of knowledge or awareness about kala Azar.They just see the patient maybe next ,next time signs may change. ...mmmh..

        f)How do you handle patients once they present to the facility with the indicated symptoms?
        RES:Whey they come to the facility, welcome them,I welcome them to the lab,I explain to them the test that we are going to do to them and also tell them about,the samples that am going to send to kemri or to Nairobi or to Lodwar.I tell them when the results will come maybe it will take a month or two weeks,so they wait for that time.But before they get that results,we have a diagnostic test that is RK 39 that one gives us results on the same day that we receive the patient.' yeah'...about kala Azar...When Rk 39 is not there, isend the…eeeeh… samples to LCRH for DAT test.
    
          g)What treatment do you offer for kala Azar within this facility?
RES :To me I cannot answer that question,that is a question that can be answered by the clinician.

*How are Kala Azar patients being followed up after treatment?
After treatment,'mmh'.they are informed to come back after three to four months.If they are still presenting with kala Azar symptoms ,they are referred for diagnosis,if diagnosis gives us a negative ,the patient is okay.When it present with a positive ,then we start treatment afresh.We start with treatment afresh,If negative you just tell the patient to go back to the community and should take care of themselves and cover,and use nets always.

h)How do you conduct stock management?
RES: on my side when I remain with almost five test kits or four,I do call the lab coordinator from Lodwar that is Mr Onyango Onyango Steve then he is the one to supply for me."diagnostic kits for kala Azar patients".. Even the sub county cordinator Mr Dismas Epeyon is also responsible for that.When we receive the stock of the commodity I have the stock card,so I normally use the stock card to monitor my stock, if i have ten maybe this week have used four ,when it remains two to Three i raise an alarm to those who are supposed to supply that is the lab cordinator from Lodwar,to the sub county cordinator for the supply

I) Briefly tell me how you currently conduct VL data reporting?
RES:I do reporting on my side,I report how many patients are there.I have a register,this register for Kala Azar and this register captures the tests that have done,it captures the details of the patient.So I do for each month...yes..

j)Has any member of the community succumbed to the disease?
RES:..Those who have died?..No .This year no..'mmmh' Thay are those who come,who have stayed with the disease for more than a year,or even six months.So when they come to the facility,after testing,when they start with the treatment..eeh..when they start treatment,you see the patient is not intuition so they end up dying.Me I have heard on one.

k)What part of kala Azar Diagnosis, treatment is most challenging for you?
RES:"microscopy"that. ..eeh.. microscopy, diagnosis of microscopy have not done.We have not done that so am not conversant with that because,I mean this one is done in big labs."conversing with a patient".

l)What part diagnosis care and treatment is most enjoyable for you?
RES:"mmh" is when I get the patient and the results comes clear either positive or negative so I assure the patient with the result that is negative or it is a positive." Talking to a patient"

*How can describe the toxicities of Kala Azar treatment drug?
RES :It is very toxic""phone ringing"..mmh' about toxicity I have not done that ,you can ask the clinicians because they are ones that know.

m)Can you tell me the relationship between HIV and Kala Azar?
RES:Okay HIV is about the immunity and the ,when someone is infected with HIV,that means the immunity is low at the same time gets infection of Kala Azar so this person becomes very weak.

n)Compared to malaria how would you rate kala Azar burden in the county?"
RES: Kala Azar and malaria?They go together.When malaria is high kala Azar also increases because this is the time now the sandfly or the anopheles mosquito breed so there is high infections when it comes to human beings.

QUE 2.
a)How prepared do you feel to handle the provision of kala Azar services within this facility?
RES:'mmh' In this facility we normally give nets to these patients and also to give awareness about Malaria,medicine,we ensure that they are treated well and also they are provided with nets.So they are informed to sleep under nets all the nights?

Que: What has made your easier in provision of kala Azar care?
Res: the supply of commodities,when there is supply of commodities it becomes on my side because it helps me identify the infection at earlier moments than when we stay without commodities so the patient also takes long to get the result on the infection of Kala Azar and even treatment.When the supply is there ,their is no shortage so patients get it at the right time.

b)What are your concerns about work demands that may come with provision of kala Azar cases in your facility?
RES: If it's possible,for kemri to get someone who is specific testing Kala Azar and also supply of commodities like the test kits , like the packing bags,specimen packing bags.If we get someone who will be responsible for that,I think we will be somewhere.

*What are your willingness on  performing Kalazar screening as part of your work routine?
Res: I feel like it has to continue.
*What about diagnosis?
RES:Even diagnosis,we feel like it has to continue daily because this is an endemic era whereby so many people are infected.

c)Has managing kala Azar cases in your facility in any way affected your work schedule or your well-being?
RES:..mmh..When it comes to testing 'ooh' eeeh'..it is more tedious because when I have other clients or other patients,..'phone beeping'..and I get a kala Azar patients I normally take time with them,it can take me around thirty minutes or fourty minutes because collecting this samples  ensure that we have the approximate labelled can take time so I concentrate more to these kala Azar patients than these other patients.Because there is collecting of DDS,DAT ,urine so before I collect the samples I have to first to explain to the patient.That's why am saying it's better if kemri or the partner they get someone who will be responsible for kala Azar only.To kala Azar patients, collecting samples it will be more better.Like now if you see I am the only one lab tech so when i see a kala Azar patient ,I stop the other patients.I concentrate with them.
Because there is filling of forms ,there is a form we normally fill,how the results have turn .

d) Have you received any specific training or skill development related to the provision of Kala Azar services?
Res:No.

e)Have you received more resources e.g personnel/equipment to help you manage the kala Azar cases following decentralization of Kala Azar services?
RES:No.They send only commodities."It has arrived so I just receive them".There is a time I don't know which when I was on my leave I was told there are people came here I think around august.They came here at the facility but have never met with them.'furniture repair around'.
"Conversing with a colleague"

f)Do you think that bringing kala Azar services to this clinic has in any way affected other services at the facility?
RES:No.It has not affected other services.It has helped us.When it comes to diagnosis,we have really improved.We have improved on diagnosis.If you get a patient actually we diagnise at the same day,the same time and when the patient ask for space we taking this assistance the same day."Lab sounds"

3.What does the community say about kala Azar and what is the impact of such perceptions on care seeking?
RES: People in the community are really happy and they appreciate how we diagnos and treat at the same facility.And this facility is like a hub so it's like a parrow point for other dispensaries and facilities because it's the only facility treating kala Azar in Loima .So this community is appreciating how we treat and how we diagnos kala Azar in this place.
The community is also appreciative,they are appreciating the treatment they are getting and diagnosis.

4)If we were to roll out kala Azar ,care and management programs to other health facilities,what areas would you recommend we improve?
RES:I think we should improve on awareness.Have awareness about kala Azar ,supply of commodities both diagnostic and treatment or medicine for Kala Azar.
And also training on Kala Azar.''mmh" what else.

5)Whom do you think should be trained at the community level to improve health seeking behaviour for kala azar patients?
RES:we have chvs,chps and the [c.h.e.ws](http://c.h.e.ws/) and also the chiefs.Ward admin ''mmh"

Any other question you want to ask me?
Res: My question is when I have shortage of commodities,and the lab cordinator at the Lodwar or the however is chaired  is not responding,Who else should I ask the commodities or how am I going to get the commodities.Right now I don't have even ,,There is a commodity that is that has run off is not there.Since I communicated there is no response.What can I do about that?

       

      
