# Supplementary material for: Barriers and facilitators of care among visceral leishmaniasis patients following the implementation of a decentralized model in Turkana County, Kenya
Source: PLOS Glob Public Health. 2025 Mar 31;5(3):e0004161. doi: 10.1371/journal.pgph.0004161 (PMC11957299; doi:10.1371/journal.pgph.0004161)
Supplement: S1 Data — This file includes the following transcripts: •VL Patient In-depth Interview Transcripts: Verbatim transcripts of interviews conducted with VL patients, capturing their insights and lived experiences. •Healthcare Worker Key Informant Interview (KII) Transcripts: Transcripts from key informant interviews with healthcare workers, detailing their perspectives on decentralized care models for VL. (ZIP) [file pgph.0004161.s003.zip › HCW and IDI transcripts/healthcare workers/Res 005_FACILITY 3.docx]

VL DECENTRALISED STUDY

HEALTH WORKER INTERVIEW – FACILITY 3

Q1a) What causes this disease called kalazar?

RESPONSE: It is caused by a sand fly…..(paper rustles)

Que: Its caused by?..... a sandfly

Res:it’s caused by a sandfly (paper rustles)

b) How is VL transmitted from one person to another?

RESPONSE:when a sandflies is bitten…aaahh..when a person is bitten by an infected sandfly….from….. from one person to another …..

Que: From one person to another?

Res: Yes

c) Which category of individuals is most at risk of VL and why?

RESPONSE: Children

Que: children

Res: because they are the once are more at risk because they usually hang around during the evenings

d) what are the symptoms that patients with VL present to the facility?

RESPONSE: Fever, stomach swelling, loss of weight and fatigue

e) On average how long do VL patients in this area take before seeking treatment and after developing symptoms?

RESPONSE: They take long time….mmh before they realize to come to the facility.

f) How do you handle patients once they present to the facility with the indicated symptoms?

RESPONSE: We receive them ……,then you take them to the CO and then the CO is the one who will direct them to do a lab testing in the lab….to verify the outcome of the disease.

g) What treatment do you offer for VL within this facility?

RESPONSE: They are injected stiboguloconate and paramomycin injections………for seventeen days (interviewer mmh)

-On how are you conducting VL treatment

RESPONSE:…(door opens and interruptions)

Que: -How do you follow up for VL treatment

RESPONSE: We follow up using the CHVs of the community.

h) How do you normally conduct VL stock management in this facility

RESPONSE: We make sure that there is enough stock for the patient ….enough stock to the patient and also it should readily available for them.

i) And how do you conduct VL data reporting

RESPONSE: The report is driven by medical laboratorist , is the person who usually do the report….mmmh for the VL patient. (phone vibrates)

j) Has any member of the community succumbed to the disease?

REPONSE: NO

K) What part of VL Diagnosis treatment is most challenging for you?

RESPONSE: Fever

L) What part of diagnosis care treatment care is most enjoyable for you?

RESPONSE: There is nothing to enjoy there. (the researcher laughed)

m) Haya….can you tell me about HIV and VL relationship or the relationship between kalazar and HIV?

RESPONSE: Yes this two diseases are related in terms of weight loss, loss of appetite and fatigue…….and that is the relationship between the two….yeah the two.

n) Compared to malaria how would you rate the VL burden in the county?

RESPONSE: It is around sixty percent.(paper rustles)

2a) How prepared do you feel to handle the provision of VL services within this facility, how prepared?

RESPONSE: I prepared by making sure that the commodities are readily available….mmmh…. at most times,…….

Que:You make sure that before they come the commodities are ……

Res: Eeeh…. …. the commodities are just there.

b) Are you concerned about work demands that may come with managing VL cases in your facility?

RESPONSE: YES, I’m concerned.

Que: Do you wish to perform screening at part of their work routine

RESPONSE: YES, I am willing to perform the screening of VL……….

Que: you usually perform screening…..

Res: yes the VL

-What of treatment as part of their work routine

RESPONSE: we are always willing………..

Int: you are always willing to perform the treatment as part of your work routine…

Res: Yes.

Que: What of on VL stock management as part of your work routine

RESPONSE: we are ready ….we are readily available for commodity management (the door was opened) for VL

Que: -What of on question on willingness to perform VL data reporting as part of their work routine

RESPONSE: We are willing ….to do the report….data report….

Int:you are always willing to do the…..

Res: Yes the report….data reporting……

Int:. the data reporting….

Res: YES.

C) Has managing VL case in your facility in any way affected you’re your work schedule or your wellbeing

RESPONSE: NO,….(long pause) it has not affected anything, it is okey….

Int: it has not affected anything concerning your work…

Res: it has not affected anything, …..it is just as a routine work …we usually do..

INT: is a usually work…

Res: mmmmmh

Int: Has managing VL cases in your facility in any ,ooh……you have already answered that.

d) Have you received any specific training or skilled development related to the provision of VL services?

RESPONSE: NO, I have not received any training…

Int: you have not received any training…

Res: YES.

e) Have you received more resources e.g personnel equipment to help you manage VL cases following decentralization of VL care in the county?

RESPONSE: NO, I have not received any resources…

Int: you have not received any resources….

Res: YES.

f) Do you think that bringing VL services to this clinic or facility has in any way affected other services at the facility? (door was opened)

RESPONSE: Haha, NO it has not affected this services….. it is just part of the routine work we usually do….

Int: it is a routine work…

Res: ..YES.

Q3) What does the community say about VL and what is the impact of such perceptions on care seeking?

RESPONSE: VL is a very bad disease……

Int: that is what the community are saying….

Res: YES.

Q4) If we were to roll out VL Diagnosis care and management programs to other health facilities, what area would you recommend we improve?

RESPONSE: We improve on the commodity, availability in the area by making sure….by making sure…..there is no stock out of the commodity, second maybe by making sure that the infected persons or children, you provide them with nets……with nets…..with nets and some food stuffs whoever those comes from far…mmmh….mmmh.

Q5) Whom do you think should be trained at the community level….level …….Whom do you think should be trained at the community level to improve health seeking behavior for VL patients?

RESPONSE: The CHVs should be trained…..

Int: why…why CHVs…..

Res: because they are the once who comes in contact with the community there, at all time.

N/B Do you have any question for….for us or any question you can ask?

RESPONSE: The question I will just ask, is….just I wanted the team to bring more knowledge that we can know more about this Leishmaniasis, terms of trainings, outcomes…(outcomes), the current regiments.. mmmh, the current regiments if any…we know those things…yeah…mmmh…

Int: thank you for your time and consideration…we…I really appreciate your information…mmmh…mmmh,

Res: welcome.
