# Supplementary material for: Barriers and facilitators of care among visceral leishmaniasis patients following the implementation of a decentralized model in Turkana County, Kenya
Source: PLOS Glob Public Health. 2025 Mar 31;5(3):e0004161. doi: 10.1371/journal.pgph.0004161 (PMC11957299; doi:10.1371/journal.pgph.0004161)
Supplement: S1 Data — This file includes the following transcripts: •VL Patient In-depth Interview Transcripts: Verbatim transcripts of interviews conducted with VL patients, capturing their insights and lived experiences. •Healthcare Worker Key Informant Interview (KII) Transcripts: Transcripts from key informant interviews with healthcare workers, detailing their perspectives on decentralized care models for VL. (ZIP) [file pgph.0004161.s003.zip › HCW and IDI transcripts/healthcare workers/Res 006_FACILITY 3.docx]

VL DECENTRALISED STUDY

HEALTHCARE WORKER INTERVIEW

**INTERVIEW**

Q1.what causes the disease called kalazar?

RES..mmh kalazar (children crying) is caused by a sandflies …. Which are found mostly in the antihills

Que…which place where you find the anthills…

Res: it mostly found …. in the reserve areas…in the mountains…places with.antihills.(inaudible)

Qb.how is kalazar transmitted from one person to another?

RES.i have no knowledge on transmission but I only know that you get from bite of a sandflies and I don’t know that it is transmitted from one person to another…..

Qc.which category of individuals is most at risk of VL present to the facility with?

RES,..mmmmh the ones that mostly at risk are herders men… to get the disease and also small children and women who live near mountains where they take care of livestock……mostly…….

Que: those people who take care of animals

Res: Yes

Que: In the reserve areas

Res: Homesteads which are near the antihills..that family can get kalazar disease …..those who live near the place…..mostly the ones who lived near the antihills are more at risk because they look after livestock…(children crying)and also those families that lived near the anthills..

Que. what are the symptoms that patients with kalazar present to the facility with?

RES:. Patients come with the symptoms of fever…aaa..would complain the..the spleen..there is spleen enlargement and also another one comes when he/she is nose bleeding and that fever,, jaundice and and all those symptoms …feels like malaria…mmh

Que.on average how long do this VL patients in this area take before seeking treatment after developing such symptoms at this facility ?...(children crying)……

RES.once they just come just they…they…sent for test for….. that disease or that once it is positive they initiate treatment…

Que:.they sent for test

Res:…to confirm…..okay…

Que: once they are confirmed,,,,……

Res: they are initiated…….okay

Que:.so what about on reasons like distance depending on those people coming from far like herders men…how do you say about the distance, the transport depending on symptoms they have present in the facility?

RES:….. .with the symptoms some they may come when the….. HB is..is.. maybe the HB is low..so we sent them to facility where they admit and given blood transfusion….and those who are stable may be the HB is above ten we also give matemics as we administer the medication,,………….continuation(sounds from other patients)…..okay………so we advice if they just…maybe allow to may be have a relative or where they can stay as they are coming to…to…be. given the…. or receiving the treatment……

Q.so how do they take or how long do they take to reach to this hospital…. facility ? in your views

RES:..to this facility….. it depends some may just come by foot…and some may be when they can sell animals and may come by motorbikes…… there is a challenge………because some they don’t have any means of coming …..okay….and there is also a challenge of….. food ….because where they stay because they might be just staying..in the relative….not even relative,,,,just a friend so they have a challenge where they come like loreng’…where far….so .there is a challenge…okay...mmh…and for those who are… weak we normally also link with the nutrition….the nutrition department so they are given some uji …maybe … cups….(inaudible) so that supplements….

Que: how do you handle those patients once they present themselves with such..the indicated /presented symptoms ????

RES.we…we…just handle them like the other patients…yes..but after knowing there is a book where we register them in the OPD…yeah…in the clinical….clinician… room……. we have a register there… that’s why we say if there is a severe may be the HB is less than ten..yeah…and the patients is may be weak we…we..we..refer to clinic seven…for transfusion first before we give the….the….treatment…

Que.what treatment do you offer for VL within this facility?...mmmh

RES..mmh we have stibogluconate..yeah and paramomycin…..

Que: .and then how do you currently conduct VL treatment???

RES…treatment…yeah…mmkh…we we we normally take the…….from when we have supply from….. either KEMSA..then we order from…….the pharmacy. Or take from the pharmacy and just give direct to the… according to the dosage ….it is calculated

Res:so you order the dosage from the pharmacy..when giving treatment…y

Res: eah…okay…..(noise from the people)….

Que: ,what about on follow ups ??

RES….follow ups…yeah..mmmmh….

Que: after treating them

Res:…..after treatment…we…we.. give the dose for 17days….then….

Que: so you give them 17 days of treatment……….

Res: yes ….

Que:.what about on drug toxicities…..???

RES…mmmh…drug toxicities….we have never experienced one here…okay..mmh………

Que: .briefly tell me on how they currently conduct VL stock management at this facilty???

RES…mmh..it just come with other orders when it is finish…it is finished…. may be we refer the patients maybe to Kakuma mission…okay..yeah…there is a time may be we have problem on shortage of…. Drugs…sometime the patients are left when they are still continuing with drugs that’s now where they seek treatment from other facilities…..(music playing).

Que.what about on data reporting on the VL cases??...

RES..so we normally register them…….okay

Que.has any member of the community succumbed to the disease??

RES:…...not yet…………okay

Que: .So what part(phone rings) of VL diagnosis,treatment is most challenging for you as a nurse???......basing on diagnosis and treatment

RES…mmmmh..treatment where……may be you have a patient…okay…where the condition is down,,,maybe the HB is low…yes...we find difficulty when initiating the treatment aaa…you see the treatment when it comes again it may cause…. may be nose bleeding or make the patients weak.. okay…so that’s the time you find it difficult to……… you see is it the treatment that will kill the patients or the condition of the patients so you just remain ….undoubt..(laughter)..mmmh…so if start the medication and the patient is weak…will they continue to suffer or…. it will or will make the patients more weak…

Que: .so what part of VL diagnosis care and treatment is most enjoyable for you???

RES..mmmmmmmhm…. may be diagnosing is good just to know the diagnosis of the patients so that…eeeh..he can get what..treatment..yeah..and also is enjoying when the patient is improving when he has come to his/her health..mmh..okay……..

Que: .so compared to malaria how would you rate the VL or kalazar burden in the county??

RES..kalazar is also coming up…yeah…there is..also…aaah..the cases are increasing…..yeh…for kalazar….

Que:….you…on .comparing to malaria is it a burden…??...

RES…yeah is a burden because…malaria..malaria just comes ,,once…,,it comes ,when is rainy season but kalazar is constant….constant…yeh…you just receive cases…even now we four cases…okay..

Que:….so kalazar is constant even in times of rainy,drought…

Res: its just constant….both the seasons..

Que:..can you tell me on the relationship between HIV and VL??? How do you relate both HIV and VL?

RES…ehhh..mmmh…HIV..and…mm..may be because…we are all…like now the… the… the….also the VL there is wasting of body…body wasting…ooh..body loss…yeah…body loss…even for HIV there is also body loss..eeh….okay….

Que: so how you do compare or how do you relate the HIV and VL??....basing on your understanding…

RES:….mmh…eee…I think they are all…….like now kalazar…kalazar is treatable..okay anybody can go to the hospital but HIV they will continue taking drugs…continuously..eeh

Que: so you say kalazar is treatable…

Res: mmmh..when patient come earlier to the….but it also kills..

Que: yes..it also kills..

Res: mmh…..so we need awareness to sensitize our people…eeh..aa. that when…when .living in such areas to be aware of such disease so that they seek…ee medication earlier….yeh……………

Que: .so lets continue…. on perceptions..how prepared do you feel to handle the provision of VL services within this facility?...(patients making noise) or what has made your work easier..basing on VL cases

RES:…how is it made my work easier when dealing eeh…is where when we have this……aaa kits for diagnostic in the lab because sometimes we have also challenges in the diagnostic we find a patient go there and go and be tested and come again.. also when the..the..there is availability of……aaa..drugs..yes…you feel good to see the patient if has been helped if he/she has gotten treatment and is going to improve…yeah….so when you have..aaa…laboratory services kits in the lab and also when we have drugs you feel happy by not again sending the patients to other facilities to seek services..

Que: so you have access drugs…okay……….(noises)..

Res: Yeah

Que: what about on…..are you concerned about work demands that may come with managing VL cases in your facility??

RES…..mmmhh…work demands…yes…okay…

Que:...so basing on willingness to perform VL screening as part of their work routine ??? what can you say about that

RES:…..in our facility we have not started admitting where cases like now for VL….. there is a time we have critical patients may be that one where the patients need…aaah.. staffs,treatment,..mmh..we..we.normally have…we have receive here stable patients….yeah it has not been our concern eeh in serving them because they have been getting services..and those who are critically we have been referring them,……….

Que: .what about on willingness to perform VL diagnosis as part of your work routine??

RES….we have been doing that…..we have never…aa…have the case and never want……to diagnose every time when the patients present with the signs and symptoms of like…ee…splenomegaly …that is our first…we check for kalazar because kalazar is rampant,,.eh..okay..

Que: Willingness to perform treatment?

RES:,..once the patients is diagnosed we just start treatment.

Que:.so it is part of work routine…

RES..yes it is part of work routine…okay,,if the patient has been diagnosed he/she is started treatment…there is no option and he or she is started on treatment

Q.willingness to perform VL stock management as part of work routine???

RES. Stock management….on stock management..some times when we have less stock…we.we.we borrow from KMH(Kakuma mission hospital)…or AMCH as we wait for the other that we have ordered…

Que: so you borrow from Kakuma mission…

Res: Kakuma mission and….and AMCH that’s clinic 7 for IRC..

Que: .on willingness to perform VL data reporting as part of work routine??

RES..yes is work routine..on data reporting…yeah………………..

Que: .has managing VL cases in your facility in any way affected your work schedule or your wellbeing???

RES..NO…okay..or

Que: Okay or have you have any challenge you face in your work schedule while handling this cases?

RES..when you have challenge you consult the..the person concerned with the….kalazar..about the dosage like now may be when you get a old mother with 70 years with kalazar so because may be the weight..does not… so we consult..

Que: so you consult from other?

Res: Clinical… medical staff…

Que: okay..so those are the challenges?

Res: those are challenges….may be you have old mother with 70 years when you give the dosage…so they will be advice..to……okay…

Qd.have you received any specific training or skill development related to the provision of VL services?

RES…mmh

Que: you haven’t received any trainings,,,,…even the skills or training about the VL cases?

Res: NO….okay…

Que.have you received more resources like personnel equipment to help you manage VL cases following decentralization of VL care in the county??

RES…haven’t received

Que: You haven’t received any resources?

Res: aaa..i don’t know may be for the lab or something I don’t know…okay…

Que: so we may say for the labs are the ones that are brought…

Res: .yeah..may be for treatment….for treatment……..may be for diagnostic only…(noises from playing phones)…

Que: Diagnostic tools

Res:mmmmh

Que.do you think that bringing visceral leishmaniasis services to this clinic has in any way affected other services at the facility??

RES.,mmmhhh….Not yet..not really..

Que: so how do you say in such case? If it has not affected the other services..so we say it is a…how do you frame it…

Res: (laughs)

Que: so lets continue……….

Que.what does the community say about VL and what is the impact of such perceptions on care seeking?

RES…on community perception when a person develops enlargement of the spleen..so there they may say this is kalazar so they say “eetid”….so they just know…because .the symptoms..okay..so they know

Que: so the community knows…..

Res: Yes

Que: .If we were to roll out VL diagnosis,care and management programs to other health facilities what areas would you recommend we improve??

RES…mmh..may be in supporting in diagnostic tools…sometimes we have shortage...of tools…also for..on..aa..medication…heeh..treatment..so it end up suffering a lot…when lacking them..so they end up suffering a lot…when they get finished….so there is also…I don’t know if there is…may be there are some..there is a case we start the disease treatment…then because there..there…is interruption on treatment then later then this patient was reinfected..it reoccur ed with the disease as I don’t know if he was bitten again by the sandflies or what?....then he embark to the facility and tested positive…..so I mean that to at least continue with those supplies to improve on services….and also centers where they will be if in any case there is complications cases so to be Identified to know that place may get services…so to take those services near to the people…yeah……..so if also be supported with nutrition supplements…because they are others where food is a problem they come from reserve to here and food is a problem

Que: there are malnourished…so nutritional support

Res: Yes……

Q5.whom do you think should be trained at the community level to improve health seeking behavior for VL patients???

RES…for now the health system is going for CHEWS because they are near to the community…so you prefer CHEWS to be trained and then also health workers like Nurses,Cos…for treatment and care…so CHEWS may be for sensitization that then when you have this signs and symptoms you need to go for checkup..and then also staffs those medical staff son how to handles signs and symptoms…yes..yeah and also on the toxicities on how it comes about and how to to look and well…. what is to be done if you have a reaction….mmmh…we have also personnels…in diagnostic…..lab

Que: mmh…okay …..thank you for your participation we have almost done I think that was the last question…

Res: yeah..

Que: or do you have something to add on….

Res: mmmh…I don’t have..…okay..so thank you for participation I have appreciated to your time for being here for me so I have got your views…okay…

Res: …so there was a question you asked me is kalazar..i wanted to know if kalazar is being…if kalazar is being transmitted from one person to the other…

Que:yes…do you have any objection.?.....

Res: (laughs)/…NO…….. I don’t know…..(more laughter)

End of interview
