# Supplementary material for: Barriers and facilitators of care among visceral leishmaniasis patients following the implementation of a decentralized model in Turkana County, Kenya
Source: PLOS Glob Public Health. 2025 Mar 31;5(3):e0004161. doi: 10.1371/journal.pgph.0004161 (PMC11957299; doi:10.1371/journal.pgph.0004161)
Supplement: S1 Data — This file includes the following transcripts: •VL Patient In-depth Interview Transcripts: Verbatim transcripts of interviews conducted with VL patients, capturing their insights and lived experiences. •Healthcare Worker Key Informant Interview (KII) Transcripts: Transcripts from key informant interviews with healthcare workers, detailing their perspectives on decentralized care models for VL. (ZIP) [file pgph.0004161.s003.zip › HCW and IDI transcripts/healthcare workers/Res 007_FACILITY 3.docx]

VL DECENTRALISED STUDY

HEALTHCARE WORKER INTERVIEW

SOCIO-DEMOGRAPHIC CHARACTERISTICS


**INTERVIEW**
a)What causes kala Azar?
RES: aah'.. "inaudible" it's caused by a sandfly..so it bites aah 'a  person so that's when someone gets infected  by that parasite ,that  Leishmania Donovani so that's theee...I think that's the most common one...yeah... Leishmania.. sandfly.

b) How is VL transmitted from one person to the other?
RES: aaah' okay..from one person to the other... probably it might be through blood transfusion….yeah

c) Which category of individuals is most at risk of getting VL and why?
RES: aaah'..Soo ... aah'..they immuno suppressed individuals for example those with aaah'...there setting here ..those with a people living with HIV,..yeah or at advanced HIV disease.Those are the ones at risk of getting aah'... Visceral Leishmaniasis...but aaah' ..or I didn't get your question?

Ooh'.. category ooh... actually it's the adults... mostly the adults.So those who look after the livestock are the ones who are at risk of Leishmaniasis. Actually it's most common among them..mmmh'...Because they go to where the ... there is this aah'..yeah pastoral communities, where they graze their livestock...there is these aah'...the name.. have forgotten the name  ..the anthill actually the anthill where the sandfly resides that's where they get.. That's where the herders.. get affected…with the .the sandfly..they graze their livestock.mmmh' ... ooh' ...

Que: How is VL a burden to the people

Res: so well..when someone gets sick so , …one is not able to go about their usual activities for example the herders them seeing their livestock aaah' so ..so.one there is an economic burden ...yeah  for example when someone gets sick they have to take them to a facility you know that transportation cost...yeah..then the .. actually mostly aah' it's about the economic aaah' that ...yeah.

d)What are the symptoms that patients with VL present to the facility with?
RES: …aaah'...most of a symptoms actually that result from the parasite affecting the blood cells..for example it's anaemia becoming, getting tired, that's fatigue and then aah'..there is a headache and then palpitations the heart beating fast and then aaah'.. aaah'.. lightheadedness .yes...and then .. that's on anaemia. They   have thrombocytopenia so they come with a starcis nosebleeding.eeeh..then aaah'..some may come with a like abdominal distension... enlargement of the spleen..yeah ..then when you go to the signs...signs eeh ...they come with aah' ..they are sick looking,and then they are febrile when you touch,they may have spleen enlargement and that is spinomegali aaah',and then aah'..they are pale .Those are the most common signs.

e)On average how long do VL patients in this area take before seeking treatment after developing symptoms?
RES: aaah'..so I will say aaah'..they take maybe  like two months...yeah .. because you know for ...the nature they pass through is they may try out some traditional interventions like aah'..they call it blood letting where they cut the.. around the spleen area,They cut then some blood get spilled over ...so some even get..go to the extent of butchering a goat you know , those are traditional interventions.Those interventions do not work that's when they come to the hospital ...when the patients are usually weak...so it's average of two months..yeah...

f)How do you handle patients once they present to the facility with the indicated symptoms?
RES:So first aaah'...youuu ..you do your physical exam ... yeah..just to check whether the spleen is enlarged and then aaah' you do blood test,in our facility here with do Rk 39...that's the one we do here.And then aaah' so  after that confirm the diagnosis that's when we start them on aah'.. treatment but then you also do aah'.. Fullhaemogram now that their blood is less and whether they need transfusion or not.. yeah so we do Fullhaemogram and so..

g)What treatment do you offer for VL within this facility?
RES:So the ones we have here is aaah' sodium stibo Gluconate and Paramomycin...stibo Gluconate..yeah then Paramomycin...P.a.r.a.m.o.m.y.c.i.n...yeah those are the ones available.

*On how they currently conduct VL treatment.
RES...come again...yeah ..yeah.

*Questions on follow up of the VL patients.
RES:oooh'... follow up... aaah' so after aah' you know when you combine the two medications, so target is to give them for 17 days then after like two weeks, they come back after completing the treatment.You do a repeat aah' HB ... yeah so we do like two weeks after completing treatment that's the follow up we do.

*So what about on major drug toxicities?
RES:On major drug toxicity... Aah'.. okay the drug toxicity that we experience here is mostly maybe aah'...bone marrow suppression..bone marrow suppression because have even have anaemia or even aah' they continue to have aah' bleeding even on treatment.yeah..so those are the yeah...the most that reactions they experience here..mmh.

h) Briefly tell me how they currently conduct VL stock management at this facility?
RES: aaah'..so actually that's the thing done by the pharmacist...yeah so there's data here , there's shit where they..they tabulate the valuable drugs..yeah..when they get stock out so we link up with the pharmacy Sub-County pharmacist so that we get the aah' ..we get supplied with the medications...yeah..so  generally thing that was the pharmacist...pharm tech.

I) What about on how they currently conduct VL data reporting?
RES:aarh' ...yeah ...so ..data reporting it  depends so we have  a various sheets for reporting,, there's the one for outpatient , there's MOH 204,205,204 biso ,we carry there and then the HRO like at the end of the month gives a ..final report yeah.. of the month.So there's only another one in the lab ...yeah..on the...on the  cases we diagnose for visceral Leishmaniasis ..yeah..based on the diagnosed cases through Rk 39.So ... there is the one in the lab also at the one...the one for MOH 204 ...the outpatient report...OPD...yeah..MOH 204A 204B.Yeah these are the register.
... aaah'..am not conversant with the ...with that..mmmh..

j)Has any member of the community succumbed to the disease at this facility?
RES:aaah'...I haven't yet met one... yeah..

k)What part of VL diagnosis treatment is the most challenging for you?
RES:The most challenging?... aaah'...well... aaah'...mmmh..eeh...I think the most challenging is diagnosis especially when you have cases of a stock out of aaah' ..lab reagents... yeah...so it becomes difficult to diagnosis case cos you may suspect the cases to visceral Leismaniasis but we at times Rk 39 is aah' ...does not...is out of stock but then don't have other cases maybe doing DAT...mmmh ...that's the most challenging part.
But in treatment, yeah...once we have the medications it's easy because we have the protocol...mmmh.. mmmh.


l)So on part of VL diagnosis care and treatment where do you enjoy most?
RES:aaaah....I will still say diagnosis..yeah.. because it's straight forward  a patient comes with a the symptoms,the signs ... yeah.

m)So can you tell me on the relationship between,how do you relate between HIV and VL?
RES: aaah'..so actually they are aaah' interelated cos when someone similar suppress it's easy one to get aaah'.. infected with a a Leishmaniasis... aaah'..yeah...then when one  gets infected so it gets a severe form of visceral Leismaniasis..yeah ..so if not handled aah' .. Promptly is one will succumb to the infection.mmmh....

n) Compared to malaria how do you rate the VL burden in this county?
RES: Malaria is aaah'...is high compared to Leishmaniasis....mmmh...okay..I will say...in terms of the prevalence or...mmmh ...so malaria is very here in Turkana ...yeah..and then Leismaniasis is somehow less...yeah...so malaria is more.

QUE 2
a)How prepared do you feel to handle the provision of VL services within this facility?
RES: aaah'....aaah'...I feel aaah'...I feel comfortable.. yeah...I feel comfortable doing the services despite the fact that aaah'...the only challenge here is the lack of blood transfusion,cos ,then a atimes we don't get a like a foteracing B.. yeah..so me I feel comfortable ..mmmh..

b)So are you concerned about work demands that may come with managing VL cases in your facility?
RES:..come up again...work demands.. I'm not ..I'm not concerned...yeah..cos here we don't get many cases as said unless well there's aah' ...maybe the stock outs of  aaah' ..ant -Kala Azar medication in  Kakuma mission .. that's when you will get aaah' overflow of many patients here.

*Specifically a question on willingness to perform VL screening as part of their work routine?
RES:..ooh..my willing to perform screening...aaah' ..so...yeah ..we very okay with that yeah ...cos we usually do that..so very okay..we do that as part of routine.

*On the part of diagnosis?
RES:Very okay...yeah.
*What about the treatment?
*
RES: aah'.. treatment side I will as well aah .. Very okay..yeah .. very okay.

*So what about stock management?
RES:.aaah.okay you know that is being done by the lab guys ,the pharm techs..Lab and pharm techs..yeah so .That is not my area of ...yeah.

*Even on the side of reporting?
RES:on the side of reporting..aah' ...you know for us we just diagnose and then we fill so reporting is basically for HRO...yeah..aah'...

c) Has managing VL cases in your facility in any way affected your work schedule or your well-being?
RES:Not really..no ..mmmh.
aaah' ..so the only challenge is when ...is when maybe we have stock outs of anti Kala Azars...yeah..For example maybe if ..if we putting a patient on a Kala Azars then it's remaining five days then one drug is out of stock,then you will have to restart again..yeah ...so that's the main challenge... yeah..stock out of medication.. yeah.
Then the other thing is we don't do that transfusion..yeah ..yeah services available here ... yeah.. blood transfusion...so those are main challenges we are facing.

d) Have you received any specific training or skill development related to the provision of VL services?
RES:No.not yet.eeeh...but I have the protocol that I requested from the county coordinator,who sent me the protocol ...am reading it on myself but haven't yet been trained...yeah...not yet.

e)Have you received more resources e.g personel/equipment to help you manage VL cases following decentralization of VL care in the county?
RES:eeeh... equipments aaah' ..I think equipments..maybe it might be the lab reagents and aah' ..I think actually we do have ,it's only the only challenge we have  occasional stock out of the reagents,that is common in all facilities... yeah...so we aah...yeah we have the..but not all ,we don't DAT,we don't do DAT so we only do RK 39....mmmh.

f)Do you think that bringing visceral Leishmaniasis services to this clinic has in any way affected other services at the facility?
RES:No.

3)What does the community say about VL and what is the impact of such perceptions on care seeking?
RES:aaah'...so..I think our community here is very well informed yeah... about visceral Leishmaniasis cos whenever they see their spleen is enlarged they usually know that they call it (ETID) kala Azar so ,and then so ...and when they notice us they do their traditional interventions that...if that fails they come to the hospital to seek for further management....yeah..so..aaah'...yeah.

4) So if we were to roll out the VL diagnosis,care and management programs to other health facilities,what areas would you recommend we improve?
RES:eeeh ..okay...so first aaah'...in diagnosis maybe we ...provisional aaah' .. diagnostic services such as aah'..maybe DATs aaah'...and then maybe training more staff especially the lab technicians on maybe DATs then other..the all test that diagnose.
Then the... ofcourse aaah' ..maybe doing aaah'...maybe by anual or even yearly training on health care workers ...yeah... so that they equiped with tha....with knowledge about diagnose, manage VL ..yeah.
And then eeeh'...yeah..yeah..

5)So whom do you think should be trained at the community level to improve health seeking behaviour for VL patients?
RES:Eeeh...okay...you know nowadays they have the... community health promoters..yeah...CHPs,they need to be trained well because they are ones who interact with the...the community on a daily basis..yeah....yeah ...mmmh..yeah....okay..mmmh...yeah...sure .. it's my pleasure.... it's very okay.
