# Supplementary material for: Barriers and facilitators of care among visceral leishmaniasis patients following the implementation of a decentralized model in Turkana County, Kenya
Source: PLOS Glob Public Health. 2025 Mar 31;5(3):e0004161. doi: 10.1371/journal.pgph.0004161 (PMC11957299; doi:10.1371/journal.pgph.0004161)
Supplement: S1 Data — This file includes the following transcripts: •VL Patient In-depth Interview Transcripts: Verbatim transcripts of interviews conducted with VL patients, capturing their insights and lived experiences. •Healthcare Worker Key Informant Interview (KII) Transcripts: Transcripts from key informant interviews with healthcare workers, detailing their perspectives on decentralized care models for VL. (ZIP) [file pgph.0004161.s003.zip › HCW and IDI transcripts/healthcare workers/Res 008_FACILITY 3.docx]

VL DECENTRALISED STUDY

HEALTH WORKER INTEVIEW

**INTERVIEW**

Q1a) What causes this kalazar?

RESPONSE: A sand fly……mmh, sand fly…..sand fly….mmmh.

b) Mmmmh…..how is VL transmitted from one person to the other?

RESPONSE: Its cause is the sand fly which is a female….aah and it also does the transfer from one person to another.....mmmh…..yeah…pardon…rudia…..sand fly still transmits from one person to another. Mmh…..mmh.

c) Mmmmh which category of individuals is most at risk of VL and why?......category……..which category of individuals is most at risk……is most at risk of VL and….and why?, hii VL ni…..

RESPONSE: Visceral leishmaniasis…mmh So mostly people from low social economic states, eeeh and also mostly the affected people are the nomads…… nomads….yeah…and pastrolists.

d) Mmmh what are the symptoms that patients with VL… vl is pre… present to the facility with?, symptoms of kalazar.

RESPONSE: Mmmmh most of this patients present with fever which has been there for more than a week or two and also…….and also present with an enlarged abdomen ….mmh for distended abdomen…….what else,…..fever, distended abdomen, general body weakness. mmmh…..yeah.

e) On average how long do VL patients in this area take before seeking treatment after developing a symptom?, on average….mmh how long do VL patients in this area..take it…take before seeking treatment after developing a symptoms?

RESPONSE: In this area I think around one to three months.

f:) How do you handle patients once they present to the facility with the indicated symptoms? How…… we handle this patients…mmh

RESPONSE: Aah….after taking the history then we do the examination…mmh, after doing the the examination then we test for various illnesses…mmh..like we rule out malaria, we do actually RK thirty nine test (39), we also test for other symptoms which the patient can present with…mmh…yeah.

g) What treatment do you offer for VL within this facility?

RESPONSE: In this facility we use a double therapy we….eeh we…we.. use sodium…we do the double therapy, so we use sodium stibogluconate…mh…with paronomycin…mmh…for a total of seventeen days…..okey….yeah.

Que: -How do you follow up the patient after treatment?, how do you follow up the patient after treatment?

RESPONSE: Aaah…..after treatment we do see the patient after six months…mmh….yeah…after six months…they are told to come back after six months but every month they do come for the testing of….for the testing of HB then they are given antifatinics

h) How do you conduct the VL stock management

RESPONSE: The Stock management….mmh…for VL drugs?....eeh….ooh actually…we just…eeh…like we request drugs we take to the pharmacist then the pharmacist starts the registration…in the whole facility. (music from the phone)

i)What of the data…col….data reporting

RESPONSE: For data reporting we have a booklet…..we have a booklet here…mmh.. …which captures all kalazar patients and at the end of the month we just collect data from the booklet…from the booklet….ye..ye..ye..ye. is a case management form for VL patients (unnecessary noise)

j) Has any member of the community succumbed to the disease?

RESPONSE: No that I know…mmh…yeah….i haven’t seen seen a patient die of kalazar since I came here. (music from the phone)

k) What part of VL diagnosis treatment is most challenging for you….part of VL diagnosis….no neglect that one…has any member of the community….ooh you have already answered that one...mmh, what part of VL diagnosis treatment is most challenging for you…..most challenging for you.

RESPONSE: Maybe Splenic aspirate which we don’t do,….mmh….splenic aspirate….mmh…mmh… which we don’t do…yeah, for the rest we can….we can do….for the rest.

l) What part of VL diagnosis care and treatment is most enjoyable for you, enjoyable for you?

RESPONSE: Most enjoyable, ….when the patients starts medication….mmh….pardon….when the patients starts medication…mmh..and the adherence is good....yeah….basically…patients starts medication……and the adherence is good…

Int: appearance or wahat….

Res: adherence….….is good….yeah. (alarm from the phone rings)

m) Can you tell me about HIV and VL relationship or relationship between HIV and VL

RESPONSE: Aah…patients…..patients with HIV…..aaah when they get VL…mmh…their response to medication is….., their mortality rate is increased…mmh… since they have a commobidity and also the common drugs that we have we can’t use them to treat patients with HIV…mmh….we use…aa….an injectable called ambisone….mmh…..which will is not raedily available in all facilities persons, I think it is only found in KMH and LCRH….mmh….yeah.

n) Compared to malaria…mmh…how would you rate VL burden in the county

RESPONSE: In the county…aaah…is less than malaria…aah…is less than malaria….is less than…yeah….is…..is less than malaria…eeh…yeah…VL cases is less than malaria cases

2a) How prepared do you feel to handle the provision of VL services within this facility?

RESPONSE: Aaah we have all the medication we have the diagnotist tests, we are ready…we are good…mmh…yeah.

b) Are you concerned about work demands that may come with managing VL cases in your facility?

RESPONSE: Work demands….aah…like for treatment of kalazar….., its….its a daily medication but it is given once a day….mmh….so mi don’t think it is too demanding.

Que: -DO you take willingness to perform screening…at…at part of their work routine

RESPONSE: Sure

Que: -What of diagnosis and diagnosis…VL diagnosis….mmh…are you willingness to perform VL diagnosis as part of their work routine

RESPONSE: Yes

Que: -What in VL treatment do you take it as….as part of work routine

RESPONSE: Yes, yeah it’s still work routine….mmh….yeah.

c) Has managing VL cases in your facility in any way or another or another affected your schedule

RESPONSE: No

d) Have you received any specific training or skill development related to the provision of VL services?

RESPONSE: Yes………which one….aaah… I have been trained on management of kalazar……management of kalazar….yeah.

e) Do you think that bringing visceral leishmaniasis services to this clinic has in any way affected other services at the facility?

RESPONSE: No…no it hasn’t.

3) If we were to roll out VL Diagnosis, care and management programs to the health facilities what areas would you recommend we improve?

Res: ....sorry if we were to?...if were to……

Que: if we were to roll out…..mmh…VL Diagnosis care and management programs to other health……to other health facilities…mmh..what areas would you recommend we improve?

RESPONSE: The areas I will recommend…aah…maybe a dispensary called Letia because most of the people come from there….mmh….yeah.

4) whom do you think should be trained at the community level to improve health seeking behavior for VL patients?

RESPONSE: CHVs…..CHVs…apart from health care workers we also train the CHVs then lets spread the word to the community…mmh…yeah.

N/B Any question, I think I’m…..i’m through

RESPONSE: Mmmmh….i don’t have any question….mmh…..thanks for your time…thank you.
