# Supplementary material for: Barriers and facilitators of care among visceral leishmaniasis patients following the implementation of a decentralized model in Turkana County, Kenya
Source: PLOS Glob Public Health. 2025 Mar 31;5(3):e0004161. doi: 10.1371/journal.pgph.0004161 (PMC11957299; doi:10.1371/journal.pgph.0004161)
Supplement: S1 Data — This file includes the following transcripts: •VL Patient In-depth Interview Transcripts: Verbatim transcripts of interviews conducted with VL patients, capturing their insights and lived experiences. •Healthcare Worker Key Informant Interview (KII) Transcripts: Transcripts from key informant interviews with healthcare workers, detailing their perspectives on decentralized care models for VL. (ZIP) [file pgph.0004161.s003.zip › HCW and IDI transcripts/healthcare workers/Res 009_ FACILITY 4.docx]

VL DECENTRALISED STUDY

HEALTHCARE WORKER INTERVIEW

**Interview**

Que: Tell more about Kalazar? ,,,,,,What do you know more about Kalazar?

Res: Kalazar is that Visceral leishmaniasis is infection that is mostly is caused by a sandfly. It is mostly found in the anthills, When you are bitten by sandfly it injects you those mascots that transmit Kalazar disease.

Que: How does it transmit from one person to the other?

Res: Through that bite..eeh.. That bite of sandflies. When bitten with the sandfly with effected with those mascots ..it will goes and bite another person that is how it reaches the disease.

Que:"eeh" Which category of individual is most at Risk of getting Kalazar?

Res: The children below age of 15 years

Que: "eeh"

Res: And this Pastrolist communities. Who are living in not in a settlement...nomadic settlement.

Que: Why does it affect children and those Pastrolist?

Res: Because those anthill are found in … those extreme areas..."eeh"

Que: For children?

Res: children are prone because of the immunity and also they are prone for the..... they are mostly used as herders they are the ones used to look after animals so they are exposed to those sites areas where antihills. Thus the risk of getting VL

Que: What are the symptoms that the patients with VL present to the facility?

Res: So usually present with the,,,,, wasting, phenomegally , Fever, they also present with anaemia, and most cases even Malnutrition can attribute to.

Que: On average how long do VL patients in this area take before seeking for treatment?

Res: That one now depends with the locality where the patients or a client is residing from. Those who are residing far away from Health facilities takes a long… a period of time, maybe 2 to 3 months. The splenomegally that more visible. they are able to identify this is Kalazar patient, they are able to can bring that Child to the facility where they can do their diagnosis very well for VL.

Que: Okay, How do you handle the patients once they present to the facility?

Res: patients that lack Normally like any other clients but we have those classical signs and symptoms for Kalazar where a cliniscian will pick that will be client who as signs and suspicion, those signs of Kalazar that they want now to investigated for diagnosis, If they are having it or they are not having it.

Que: Okay, What kind of treatment do you offer for VL in the facility?

Res: We haveboth the long term and the short term treatment "mmmh"

Que: How do you conduct the VL treatment?

Res: the treatment they are … they are given injectable

Que: yes

Res: The injectables that is given for 17 days "mmm" That is paromycin and SSG if they are given a single for SSG they are given for 28 days if they are given a combined dose of SSG and Paranomycin they are given for 17 days. And also those children who are below 2 years they are given ambisom.

Que: Okay,

Res: "mmmh "Que: Do you usually follow up VL patients after treatment?

Res: We follow up rarely, rarely do we do the follow up. When the patient gets healed we are done… . we rarely do the follow up, but if we get any relapse, maybe someone have been treated for Kalazar and come backs with the same symptoms then we advise for parasitological techniques to rule out if it is a VL or other infections that are in patient…the patient is having.

Que: Do drug given affect them? In terms of drug toxicities...

Res: "yeah " to some extent they are affected like SSG it depletes the haemoglobin level for clients so you get maybe if you identify a clients who came with haemoglobin of 7 and was injected with SSG without dose and the.....they are no blood it becomes the challenge, that challenge ends up to be anaemic because of the treatment of the injection to those Daily injections, they depletes the haemoglobin level and the child ends up being anaemic at the end of the treatment. So we keep monitoring the HB as they being given those injections, so we keep monitoring the HB's level else not to create an alarm on another side of as we also giving the treatment.

Que: How do you conduct the VL stock management?

Res: In terms of commodities or ....

Que: Drugs commodities or data reporting how do you conduct it.

Res: so…commoditie Mostly we are supplied through the county, by the aid of the coordinator.. the county coordinator on a Neglected Diseases.

Que: "yeah "

Res: The commodities in terms of drugs, in terms of diagnostic kits, sometimes we also get the privilege to get from other partners like MSF who are also partners from our side here, we do get some drugs here and diagnostic kits from them

Que: Okay, As any member of the community succumbed to the disease?

Res: "yeah " they are there. Yeah they are. Sometimes you get a client who comes to the facility when he,,,,is really chronic, in chronic stages and maybe you ..maybe immune-suppressed not only the Kalazar maybe is infected with HIV or any other chronic illness. So we end up losing that client even before we start the treatment because we cannot change the treatment until we stabilised the client or someone the client come with TB and He is also having Kalazar

Que: So it is caused by delay of coming to the treatment earlier?

Res: yes, coming to the health facility to seek for treatment services makes the client succumbed but those who have been identified at the early stages they don’t succumbed for the disease.

Que: Okay, What part of VL diagnosis, treatment is most challenging for you?

Res: In diagnosis.....VL diagnosis mostly they are using the RK 39 that is a rapid test and also using the DAT. The RK 39 is A rapid test, it takes short period client get the results from after 20 min …22 min they client would have get screen and be identified whether it is VL positive or a negative but now the DAT detects a bit of time because the procedure are long take around 18 hours to get the results. It is the whole day so if you get maybe a client who come from far facilities maybe from remote areas …it will become a challenge for him even to wait for the results, sometimes are bound to be like admitted to the ward for observation they get the results, If positive they are initiated to the treatment.

Que: Which part of VL diagnosis, care and treatment is most enjoyable?

Res: There is nothing like enjoying on this, it is just a service. May it starting from treatment, management, diagnosis...it is just a service, we acknowledge we get a VL patients maybe in a chronic stage he as get the right medication and comes out to be healed, we acknowledge that effort..."yeah" but if you are managing a client and succumbs because of VL or maybe compromised with other conditions those are sad stories we don't like to hear.

Que: Okay, Compare to Malaria, how would you rate VL burden in the community, facility and county level?

Res: VL in relation to Malaria, Malaria here is high, Malaria here is high and VL in comparison to the county in Turkana West where we are at the moment, you know we are neighbouring Ethiopia, we are neighbouring Sudan also neighbouring Uganda on the other side..."yeah" and this is a nomadic… Pastrolist swho move with animals from place to another so the high chances like the..the..the prevalence of Kalazar here in Turkana West or Lokichoggio is higher than the other parts of Turkana that is because of that nature of boundaries and the movement and the nomadic nature so the burden of VL in Turkana, in Lokichoggio is higher compared to other parts....yeah"

Que: Okay, How Does VL relate with HIV?

Res: No that are two different things.

Que: mmm"

Res: "eeeh" HIV is a viral infection while a VL is a parasitic infection…..this are two different and they don't correlate in anyway.

Que: How prepared do you feel to handle the provision of VL services within this facility?

Res: The system that have been put in place

Que:"mmmh "

Res: The system that have been put in place for triaging the patients, clerking, diagnosis, treatment and even admission and even giving supplements if there need of ..maybe there is Malnutrition or co-infection of maybe TB they are able to get all the services with the facility is not like maybe you go to and refer the patients for the other services noo. When the patient walk in the facility they are able to be identified, they are to be diagnosed, they are able be tested, they are to be supplemented if there is need for any supplementation and all the services that are there in one room, even when there is need for blood transfusion… issues they able to do get blood within the facility. So this facility is all round for VL occasions can comfortably get the services with at least..

Que: Concerning about work demands that may come with new managing VL cases in the facility, are you will to work with it, in part of screening...

Res: yes we have been doing it, here at facility…at the outreaches so we have been doing it, it is something that is on going

Que: Will it not affect your work routine?

Res: It does not that is part of the routine work.

Que: How managing VL cases in your facility in any way affected your work schedule or your wellbeing?

Res: No no. It does not affect in any way. It is just part of the routine activities that we are doing..it is just part and parcel of the activities that we are doing

.Que: Okay

Res: "mmmh" they are just part and personable activities.

Que: Thought it affects you, in terms of work hours...

Res: …Maybe in term of diagnosis we are using the DAT …. which takes a bit of time, maybe that lengthy period for waiting for getting results maybe… it can a patient not to patient and sometimes maybe it might walk out and go home not come back to get the results, maybe you get those missed opportunities.

Que: Is there challenges while diagnosing, care...

Res: There is no challenges just normal algorithm we just apply.

Que: No challenges?

Res: yes just the normal national algorithm for diagnosis, treatment and management for VL cases still stands, we haven't received any update for any changes of diagnosis and management of VL

Que: Have you received any specific training or skill development related to the provision of VL services?

Res: Training are very....its a challenge actually, most VL health workers we are …disadvantage in giving capacity building in terms of training, to get more updates on issues of VL and also to keep and also to refresh those who have never handled this cases of VL maybe interms of diagnosis of VL for the new staffs. So there is need for that trainings and capacity building, even workshops and even in meetings in other facilities

Que: Is there any training have you attended?

Res: Within the facility No, outside the facility is very minimal, very minimal.

Que: Could you mind to share your experiences?

Res: Most of the time you see,.. what happen in Turkana most of this bosses are the one goes to the Training, so after going to the training they just come and give us the a bit of the updates from the training. But it would be….it could be more easier and more comfortable if we take the person who is at the service delivering point, that person who is dealing with the client to be capacity builded so that when he comes back, He does exactly what have been updated with, equipped with unlike getting the information from a secondary persons message so maybe something is a challenging so they need to check on it, on identifying diagnostic and management areas of facility that are managing and diagnosing VL so that the officers who are working on the ground hands on with the client are the ones to be capacity builded , trained and equipped with the skill to VL management and the other things that is related to VL.

Que: Have you received more resources eg. Personnel equipment to help you manage VL cases following decentralisation of VL care in the county?

Res: Resources,,, maybe in terms of reporting tools "mmmh yeah" in terms of reporting tools and diagnostickits that…eehhh and drugs but any other resources maybe to motivate even the staff working at that particular end there isn’t.

Que: Do you think that bringing VL services to this clinic has in any way affected other services at the facility?

Res: No it has not affected but it has only complemented the services…

Que:"mmmh"

Res: "yeah" it really complemented the services and enhanced the diagnosis and management of those client, so having VL done here, managed here….. It has even made this place to be a referral site for other facilities that are neighbouring this area. So it was great impact and there is need for them to capacity build and re-model the facility and also we equip the personnel because it is a referral site of getting all the patients from the other facilities, they are the one managing those patients, they are the ones treating those patients so there is also need for that capacity building.

Que: What does the community say about VL?

Res: It is a positive, because now they are getting the services in there door step,,,yeah, for only a few come from far areas …there is disadvantage but also most of them, they privileged because the treatment is free, the diagnosis is free. Everything we doing for them is free so it is a positive indicator.

Que: If were to roll VL diagnosis, care and management programs to other health facilities, what areas would you recommend we improve.

Res: Be even you enroll, you need to…strengthen the existing, they need to strengthen the existing facilities so that they run …they are having everything they need…because VL is not just the issues of diagnosis and management. There are other things that goes in line with VL like issues of Malnutrition have told you, we have the issues of coexistence with other diseases HIV, TB and even the cancer we have the issues of blood components which is very essential, there is the issues of supplements because they also need supplements to be there ...yeah

Que: Whom do you think should be trained at the community level to improve health seeking behaviour for VL patients?

Res: Maybe the CHV'S… the Community Health Volunteers, they need to be given that awareness, they need to be sensitized on the issues of VL. So that they are able to identify the clients of the VL, right away from the community and refer them to the facility for treatment and diagnosis .

Que: I think we come to the end of our interview, "mmm" is any question you would like to ask?

Res:,,,,,aa.. actually no question to ask…. but I just want to emphasis, on the issues of capacity building of the health care workers who are hands on with the clients, two…they also need to have a little budget to see how... because most of the VL patients basically Kalazar actually lack blood component and affecting the blood component most of the clients who are,,, most the clients who are having Kalazar their haemoglobin level are always down. So they need to think on the issue of the blood components like how do you get this blood components within our facility...and have that sustainable program, that at every one moment we are having this product at our dispersal so that this patients are able to benefit from this services and are able to get this products for their own health or their own growth. Then maybe the last thing is issues of personnel also to…. sensitise more personnel and sensitise more health care workers those of VL, bring new updates, bring new diagnostic skill with the shorter period so that the patients results promptly. That is all I can say.

Que: Thank you for the participation.

Time: 21min 31sec.
