# Supplementary material for: Barriers and facilitators of care among visceral leishmaniasis patients following the implementation of a decentralized model in Turkana County, Kenya
Source: PLOS Glob Public Health. 2025 Mar 31;5(3):e0004161. doi: 10.1371/journal.pgph.0004161 (PMC11957299; doi:10.1371/journal.pgph.0004161)
Supplement: S1 Data — This file includes the following transcripts: •VL Patient In-depth Interview Transcripts: Verbatim transcripts of interviews conducted with VL patients, capturing their insights and lived experiences. •Healthcare Worker Key Informant Interview (KII) Transcripts: Transcripts from key informant interviews with healthcare workers, detailing their perspectives on decentralized care models for VL. (ZIP) [file pgph.0004161.s003.zip › HCW and IDI transcripts/healthcare workers/Res 011_FACILITY 4.docx]

VL DECENTRALISED STUDY

HEALTHCARE WORKER INTERVIEW

**Interview**

Que: What do you understand about Kalazar in terms of …what causes it?

Res: It is a Visceral leishmaniasis also called Kalazar..in the common language…aahhh… It is a tropical disease that affects mostly the northern arid lands of Kenya. And…. It affects…eehhh… the major organs of the body for example the….. liver, the spleen and the bone marrow….and aahhh….its aaaa… its symptoms include the fever…massive wasting…massive body wasting and sometimes it can cause severe anemia and,…aaahhh.enlargement…massive enlargement of the spleen and the liver. Those are the classical sign of visceral leishmaniaisis. And then visceral leishmaniasis is also caused by the …its vector is called sandfly and this sandfly mostly inhabit the ……the unused anthills…the evacuated anthills…yeah

Que: and which category of individual is most at risk of getting VL?

Que: okay….it is aaahhh…visceral leishmaniasis is a immunosuppressive ….immunosuppressive….disease, so people living with HIV/AIDS are mostly affected…, pregnant women and children with Malnutrition……the immunosuppressive state

Que: Why does it affect people living with HIV, children with malnutrition? Why does it affect them?

Res: as is said, VL is an immunosuppress state…aaahhh…disease mostly when it affects the bone marrow and your immune status is also low..it causes the condition of the disease….yeah

Que: What are symptoms that patients with VL present to the facility with?

Res: okay…They present with aaahh..very high fevers, massive body wasting, they also have skin lesions….multiple discolored skin lesions especially the site of the bite and they might be having what we call hepatospleenmegaly…the enlargement of the spleen and liver……yeah..those are the classical signs of visceral leishmaniasis

Que: On average how long do VL patients in this area took before seeking for treatment after developing symptoms?

Res: okay…Mostly they take more than 30 days onwards to…to 8 Months.

Que: What made them wait that long?

Res: The challenges include; they came from long distances, …aah…inaccessibility to health care facilities and also, "eeeh " poor information …poor information and also inability to access nets.,…insecticide treated nets (ITNs)

Que: How do you handle patients once they present to the facility with the indicated symptoms

Res: Okay the patient who presents to the facility we subject them to investigations in the Laboratory to rule out other diseases and one of the investigation is …we usually do a aaah……deep stick examination of a aah…(bird chirps) VL and also we usually take blood samples for VL

Que: Whom do you take Blood?

Res: The patients…the patient is the one we take out blood…and also we rule out other co-existing conditions…yeah

Que: What treatment do you offer for VL within the facility?

Res: okay….We have a combination of paramomycin which is 25mg per Kg body weight per day and also we have sodium stibogluconate which is 20Ml/kg. …wait no…paramomycin is 15 mg per kg body weight and sodium stibo gluconate which is 20mg per kg body weight per day. We give a combination of 17 days. And also if we are under stocked with paramomycin..we usually give Sodium stibogluconate as a long…as a long drug for 30 days. That is what we have at the facility so far

Que: Do you follow up VL patients after treatment?

Res: yes…We usually follow up the patients after treatment. We usually do a after 3 months follow-up on the patient. We do usually do a diagnosis test after three months

Que: Do the drugs given affect the patients in terms of…aah…are they toxic to the patients?

Res: yes, most of these drugs are very toxic to the patients so we usually do…maybe sometimes we treat along other condition for example they…hydrate the patient, can give painkillers. ..yes…Just to make sure the patient is comfortable.

Que: How do you usually currently conduct VL stock management

Res: stock management is usually by…aaahh…. according to the commodity that are in the pharmacy, we liase with the pharmacist and aaahh with our client who are present at that month, so we order according to our patients.

Que: And how about the report …who do you present it?

Res: the report usually has..aaaah..We usually have this form here that we report to Neglected Disease …aaahh..Unit at aahhh…County level every month, so every VL patient who is positive or every suspected VL patient we usually report to the Neglected Diseases Unit at the County level every month…..monthly….yes

Que: Has any member of the community succumbed to the disease?

Res: yeah…, we have some few deaths that are reported but they had coexisting infections like Malaria and other maybe typhoid.eeehhh

Que: so has that made them to succumb…or

Res: No…it is not alone…sometimes people succumb not because of VL alone..they come with other co-existing conditions…okay…yeah…so the answer is yes…we have a few clients who have succumbed to visceral leishmaniasis

Que: How many are they?

Res: If I can..aaahh…in the last year at least i can say maybe 2.

Que: Okay.

Res: yes.

Que: What part of VL diagnosis, treatment is most challenging for you?

Res: aaaahhh…The most challenging part in our…aaahh, when it comes to presentation..aah diagnosis and treatment, ….I think it is the treatment part …..the treatment part is the most ineffective way, because aaahhh… most of our clients come from long distances…so getting…them sometimes….aaahhh they miss drugs and the stockouts also from the county level…we have some stockouts of drugs so if we could be having adequate medication and alsothe partners… some partners have withdrawn from co-existing with us when it comes to drugs management like MSF….so that’s a major challenge also and this drugs also are very expensive to procure.

Que: What part of VL diagnosis,care and treatment is most enjoyable for you?

Res: The most enjoyable part is "aahh..." getting to…aaah..getting a person with Visceral leishmaniasis and is healed that is the most enjoyable part because I have achieved your target…..thats the most enjoyable part

Que: Compared to Malaria, how would you rate VL burden in the facility?

Res: Malaria has a massive…has a massive prevalence here, and I think according to our records this is an endemic zone of Malaria now, because of our reports that we usually send to the NTD Unit …yeah…so VL is not that much nowadays because of effective treatment and diagnosis mechanism.

Que: How does VL relate with HIV?

Res: according to whatever I said in the first …aahhh…whatever you asked me the first time..aaahh..VL is immunosuppressive and HIV also is a immunosuppressive so coexistence of VL and HIV it makes the condition worse…because all of them are immunosuppressive states

Que: How prepared do you feel to handle the provision of VL services within this facility?

Res: Okay…We are adequately prepared, because since the ….Neglected Disease unit of the county level was established, we have been having adequate supply of drugs and the diagnosis test. Yeah and manpower and training…most of us are trained on how to handle VL patients

Que: Are you concerned about work demands that may come with managing VL cases in your facility?

Res: Failed cases?

Res: Yes it’s a concern if we treat and maybe there was inadequate preparation of the healthcare worker

Que: Are you concerned about work demands that may come with managing VL cases in your facility? In terms of Maybe screening.. Work routine

Res: Okay…maybe the training part is what I am concerned about, training more health care workers when it comes to handling diagnosis and treatment "Health workers noise"

Que: What about top management?

Res: and also top management…yeah

Que: Has managing VL cases in your facility in any way affected your work schedule or your wellbeing?

Res: Aaaahhhh…not really…not really, because eehh..normal routine examinations…aaah..of other co-morbidities are okay

Que: Is there any challenges you face while taking VL care, diagnosis and its management?

Res: aaaahhh…yes, maybe when it comes to adequate diagnosis….it becomes a problem. Sometimes…aaah…diagnostic tests are aaah…diminished We have to look for other ways to diagnose VL.

Que: okay..and have you received any specific training or skill development related to the provision of VL services?

Res: not really that is… maybe on job training only

Que: okay..on job training…..do you mind to share your experience?

Que: Could you mind to share your experience

Res: yes, I would like to share my experience with the other clinicians…

Que: okay tell me about it…

Res: I would like to share…maybe on the diagnosis we are doing…the rapid…eeh rapid diagnosis of RK 39, RK38 and also DAT that we are doing well right now to my fellow staffs. I would like to also share the diagnosis aspect….maybe the clinical symptoms and signs with my fellow staff through CMEs that we usually teach people every Wednesday….yeah

Que: Do you think that bringing VL services to this facility has in any way affected other services at the facility?

Res: Not really. No services have been affected yeah

Que: What does the community say about VL ?

Ans: About the?

Res: About VL in terms of infection, perceptions?

Res:okay….We have empowered them…to know what to do…we have empowered the community health care promoters to know the signs and symptoms so they disseminate this information to the local community units. Yes…so they are fully aware on clinical signs and they bring to the hospital wherever they suspect there is a client.

Que: what if we were to roll out diagnosis, care and Management programs to other health facilities, what areas would you recommend we improve?

Res: Aaah..we improve…we just improve the diagnostics… the diagnostics to be at the lowest level of health care delivery…yes... to be at the lowest level so that when community health promoters suspect a VL client, to be able to diagnose from the community unit …that is what should be improved.

Que: Whom do you think should be trained at the facility level to improve health seeking behaviour for VL patients?

Res: I think from the …pharmacist ….to the clinicians …..to the medical laboratory technologist and the doctors. I think every health care worker should be given priority yes.

Que: I think we have come to the end of the interview….is there any question or anything you could share?

Res: No I was very happy because eehhh…giving us this chance to share our views concerning viral load(laughs) not viral load…..visceral leishmaniasis and its good that sometimes …incase we find these patients that don’t respond to treatments we need help when it comes to that…..yes…we need help..because sometimes clients come (Interviewer interrupts)…..more research and diagnosis..we need help on that…

Que: Okay. Thank you for participation.
