# Supplementary material for: Barriers and facilitators of care among visceral leishmaniasis patients following the implementation of a decentralized model in Turkana County, Kenya
Source: PLOS Glob Public Health. 2025 Mar 31;5(3):e0004161. doi: 10.1371/journal.pgph.0004161 (PMC11957299; doi:10.1371/journal.pgph.0004161)
Supplement: S1 Data — This file includes the following transcripts: •VL Patient In-depth Interview Transcripts: Verbatim transcripts of interviews conducted with VL patients, capturing their insights and lived experiences. •Healthcare Worker Key Informant Interview (KII) Transcripts: Transcripts from key informant interviews with healthcare workers, detailing their perspectives on decentralized care models for VL. (ZIP) [file pgph.0004161.s003.zip › HCW and IDI transcripts/healthcare workers/Res 012_FACILITY 4.docx]

VL DECENTRALISED STUDY

KEY INFORMANTS INTERVIEW WITH HEALTHCARE WORKERS

**INTERVIEW**

QUE 1.   So I want to ask you about the knowledge you have about VL…Visceral leishmaniasis also known as kalazar         

         a ) So tell me what causes the disease…the kala Azar ?
   RES:  Kala Azar is caused by a sand fly.

           b) How is kala Azar transmitted for one person to the other?
    RES: mmmhh…Through "eeh" a bite from infected sandfly 'mmh'

           c) Which category of individuals is most at risk of the getting kalazar and why?

(birds chirping in background)
     
   RES: Mostly the under 5years and the elderly due to low immunity (child cry in the background)

Que: and its burden…how much do you think kalazar burden is?

Res: as in ?

Que: You have told me that the most at risk persons to get the kalazar is the under 5 and the elderly persons …which area do you think that kalazar is prone and how is the burden in that area?

Res….long pause

Que: okay…lets continue…You said that the under 5 and elderly are most at risk…and you also mentioned about the low immunity system in them….so tell me how is the burden and what areas do you think that the kalazar disease is most found?

Res: The area that ''(clears throat")….okay' ..the kala Azar disease is mostly found is among the people who live in reserve areas mostly in the bush….like maybe they go to herd cattle and then the sandfly bites them ,and that's how they get visceral Leismaniasis.

Que: What are the symptoms that patients with kala Azar present to the facility with?
RES: okay……Most of the patients come with fever, weight loss, some have abdominal distension and others have low Hb.
(birds chirping in background and a slight interruption)
 Que: On average how long do kala Azar patients in this area take before seeking treatment after developing symptoms?.....how long do they take?
Res:……like most of them can take maybe a month ….from a month going forward because you find most of them come from reserve areas, the means of transport….the means of transport before reaching the facility may not be accessible so they take time to come to the facility ('chats from outside').

Que: How do you handle patients once they present to the facility with the indicated symptoms? How do you handle them?
RES: So first of all, …we take the complains and …..then do the physical examination, after physical examination we send them to the lab to do the test.               

Que: What treatment do you offer within this facility? How do you offer VL treatment? 
RES: 'okay after the…… after it is confirmed that it is the…...the patient has kala Azar ,we have IV stibo Gluconate and then I.M Paramomycyn.So when you give IV Stibo Gluconate we combine with I.M Paramomycin and we give them for 17days.But sometimes,…Sometimes you find that there is a time when the Paramomycin is not there so ….we give the stibo Gluconate for 30days." Have you understood?'

Que: How do you currently conduct follow up on kala Azar patients after treatment?

RES: So after treatment, we give them '..aaah..' T.C.A of one month; come back after one month or is it two….we give them a TCA of one month but if they don't come sometimes we use the 'how do we call them ?' community volunteers to look for them."if they don't come back, because we have their data with us'.

Que: How do you currently conduct VL stock management?

RES: By recording….we record the number of Stibo Gluconate and Paramomycin they take on the stock green card….stock card.

Que: How can you describe the major drug toxicities on kala Azar patients?......I can ask in Kiswahili…what are some of the drug toxicities you see once you administer the medicine to the patients?
RES: "drug toxicities' "……. okay most of them when you give the…IV stibo Gluconate,they say like it burns when entering the body. Even if you are using a branular they still say, it burns them .

Que: What else?

Res: That's all I know.

Que: How do you currently conduct the VL stock management?
RES:……We record the number of…the number of Stibo Gluconate and Paramomycin we take.We record in the stock card. And again we have a register,that we register the injections we give out and so we note down.

Que: Which booklet is that?

Res: It’s a book…not this registers,,,it’s a book we improvised

Que: Briefly tell me how you conduct data reporting?
RES:"mmh "we conduct data reporting through using the book that….we.. record the daily injections those of Paramomycin and Stibo Gluconate.

Que: Has any member of the community succumbed to the disease?
RES:..like…..'which period ' or just any…..yes there was a time

Que:How many people died or how is the mortality rate?

Res: The mortality is low but mostly the thing that ends up finishing them is anaemia , because you find most of them…..you see the way they say you cannot start medication while the Hb is very low..its a must you transfuse till the Hb is okay so that you start the medication. So…there are some patients they come…..you can find that a child has come with the mother…they have come from reserve and the Hb is low…..Sometimes you find that our lab doesn't have blood and when we try getting help from Kakuma there is no blood there.So you have to mobilize the relatives to donate and you find a mother has come with the child by herself . The mother sometimes you can find that she is pregnant and so can't donate blood or maybe she doesn't meet the criteria for donation, so you find that you are stuck on how to help the child.

So between that time you are still looking for a blood…blood ,you get that ,,like the one I remember passed, a child who died, the issue was blood. He didn’t have blood…he was paper weight . And then we give him blood…the first blood …the only one we could get from the lab at that time. Now it needed them to mobilize the relatives or donors so to donate so that he could be transfused so the blood could shoot to normal,,by the that time the mother was looking for someone to donate the …..,the child died(uncler voice). Anaemia is what is finishing them.

Que: What part of the VL diagnosis, care and treatment is most challenging for you?
RES:….'like personally'.. Everything is fine despite like challenge we can get through diagnosis is when you do your part and you send the patient to the lab sometimes the sample is supposed to be sent to Kakuma,so that way the patient is supposed to provide with fare like she should give out the money so that the sample should be sent.You find most of them tell you they don't any money to send the sample.
In terms of treatment there is a time we lacked stibo Gluconate and Paramomycin or sometimes we only get stibo Gluconate only and also there are times we had the stibo Gluconate and it became over and you find a patient get only for 10 days and stibo only should go for 30 days.So you be in suspense what to do in such cases, what period do you give the patient to come back. And when they come back do you restart treatment or continue from where you left? That becomes a challenge.

When the patient has received only half the dose….like the patient is halfway with treatment and it becomes finished and he/she is expected to come back when the medicine comes….there lies the challenge for us….like you don’t know whether to continue or restart medication .
(birds chirping loudly)
Que: What part of kala azar diagnosis care and treatment is most enjoyable to you,? Which part of the work makes you most satisfied that you have done the job well?

RES:"mmh" "treatment"..it's enjoyable when all the drugs are available and the patient respond well .You feel good when a patient respond well to treatment.

Que: Compared to Malaria, how would you rate the VL burden in the county?

RES:' okay' if I compare the kala Azar burden to malaria, for kala Azar,….its…first', ' I don't know how to say it' kala Azar,it takes some time to do the diagnosis but for malaria it's instant and then for kala Azar "aah…. ..okay for malaria it's easier to treat malaria.'the..eeeeh… the,,eeh…'like the drug for malaria can be available…. like you can find the drug for treating malaria at…any point …at any pharmacy… like it's easy to find the treatment for malaria but ,for kala Azar,it's hard like you may find the stibo Gluconate and paramomycinis not there so you decide to beg from other facilities but malaria even the patient can buy the drugs from the chemist. But for kala Azar case if it's not in that facility there's know where you can beg from and there's no way you can send a patient to buy that drug.

Que: Can you please tell me on the relationship between HIV and VL?
RES : 'okay for the HIV patient,the immunity is low ,so the patient can easily contract the kala Azar due to low immunity and then when the patient,like…,after the patient conrtact the eeeeeh"  the HIV patients gets kala Azar now the patient will be weak and the patient can easily ,,"okay the patient may respond to treatment but now,the patient will suffer because he/she has two things and now that Kalazar parasite drains/sucks blood ,so,the patient will suffer.

Que: How prepared do you feel to handle the provision of Kalazar services within this facility?

What has made your work easier in provision of kalazar services?
RES:It's easier to provide the Kala Azar treatment when the treatment is available,now we have the Paramomycin and the Stibo Gluconate and that Ampisom it's easier but a  challenge comes when there is no drug. But for now the drugs are there.

Que: What are your concerns about work demands that may come with managing Kalazar cases in this facility?
 Res:okay…fine.. First of all ,,like when the Kalaazar patients come,we treat them as outpatient like they come for the injection….they go and come tomorrow .But you find that they come morning hours but at times you find that there's only one nurse who is handling the general ward rounds plus the outpatient cases. So outpatient include those with Kala Azar patients, So you find we tell them to come at 8am in the morning. Wo when they come They find that at 8am you are alone ,you do the ward rounds,you are in maternity , paediatric ,you are in nbu like you have to do everything. So you sometimes have to ask them to wait, so they and wait upto when the sun becomes hot,a mother tells you 'I have trecked with the kid ,the sun is hot now and then you wonder how you can help her. So The problem is staff are less so you find sometimes the patients delay "mmmh"

Que: How willing are you to perform VL screening as part of your work routine?
 Res: Aaah' so when the patient presents with the…eeeh ' after a patient comes we do history taking,…we do physical examination "okay I do' .The patient comes presents with the symptoms so we take the….eeeh' the history and then physical examination ,after physical examination you can stabilize the patient if the patient has come with fever so you give the paracetamol drug ,after giving paracetamol you send him/her to the lab for sample collection and then comes back to the ward. If it's a seriously sick, patient will admit for sometime,you know sometimes a patient can present with those kala Azar symptoms let's say he/she has been diagnosed of kala Azar and malaria.So we will treat malaria first and when stable we introduce to kala Azar treatment.

Que: How willing are you to perform the VL treatment as part of your work routine
  Res: Am willing to do ,,…..to perform the kala Azar treatment at any time as long as the requirements are there,….mostly the drugs.

Que: How willing are you to perform VL stock management as part of your work routine?
Res: By ""eeeh... By recording every time when I take the"what"…..the….the … the drugs and record on the stock card. (long pause) I record and balance.

Que: How do you perform data reporting as part of your work routine?
  Res: By….i do data reporting by….You record the details of the patient, on the book,….there is a book in which we write inside.

Que: Has managing VL cases in your facility in any way affected your work schedule or your well-being?
RES: Maybe yes or no because just like I said initially, sometimes you are only one staff,…like here we have staff shortages so you find its you one staff for general ward, and at the same time, those kala Azar patients are waiting for you. You see that sometimes they see like you are delaying them and you see you have that burnout ….you see…but we try to make sure we give them the drugs. The problem is the shortage of staff. You see it is unfair for a mum to come at 8am and trekked all the way from far and she waits to alomost 1pm.

Que: How many hours do you think you overwork?
Res: We don't overwork because of kala Azar patients because administering the kala Azar treatment doesn't take even 5 minutes,..you see,.The problem is when you are alone ,you do all  ward rounds and they are all waiting for you. It is not about me overworking…just that it is them that I feel sorry for, ...You see that you are delaying them since 8am…..and it's just like you going to a facility at 8am and being attended to at 12pm ,you can see that it is not fair. And it's not that they have money to get a motorcycle and she has come from her with her child on foot. Then leaves the facility when the sun is too hot,so you find that most of them always prefer if they come at 8am  so you see the sun isn't hot yet, be given the drugs at least he/she would go .
So the bigger problem is the patients delay not the health care worker overworking. ..Problem is the delays caused to the patients

Que: Have you received any specific training/skill development related to the provision of VL services?
RES:No no no.We are using knowledge acquired in school…..'Okay the school knowledge and CME. ….Sometimes okay knowledge I can say number 1 is that of school then that of CME but going anywhere for training not yet …..Just the facility CME.

Que: Have you received any more resources either personel /equipment to help you manage VL cases following decentralization of VL care in the county?

RES: No.


Res: Do you think that bringing visceral Leismaniasis services to this clinic has in any way affected other services in this facility?

RES: No.

QUE: What does the community say about kala Azar?

RES: What the community know about kala Azar is that it's a deadly disease

Que: Any other thing? Their perceptions about kalazar

Res: 'mmmh' most of them see that ,like they come to you and say that,that drug you gave us is not helping.Some don't believe that the drug would help because sometimes you find,you have given medication for let's say 10 days and then this mum comes with the child and she said " the child has not improved…you know what they say.....They come saying that the drugs are not helping

Que: What are the impact of such perception on health seeking behavior?
Res: So when they say that, Once they say the meds are not helping,...eeeh..You find that some…not all…so sometimes you find that even if you tell them…you see after they finish treatment you give them a TCA…so you find time you that after completing treatment, they don't come back. He/she decides to stay back in reserve and not to come back.

Que: If we were to roll the VL diagnosis, care and management programs to other health facilities, what areas would you recommend we improve?

RES : mmh….the area you are supposed to improve is ,they bring more, those testing kits… , like they should make sure at any given time they are available in the lab so that in that way it will make our work easier,and so there is no way you will tell the patient to look for money to send blood sample to either Kakuma .alot of time is wasted and the patient is suffering
They should also provide with treatment sometimes the drugs are not there ,and if possible they can increase the number of staff ……….. (''greets a colleague')

QUE 5: Whom do you think should be trained at the community level to improve health-seeking behaviour for Kalazar patients?
RES: They should train the community Volunteers,

Que: Who else?

Res: The chiefs,” …..The community at large .You know now once the community knows this symptoms….like if you see this symptoms go to the hospital …..this ,this and that ,they should tell you ,'do this go to the hospital and get drugs'.
Do you have any other questions to ask?
RES: what I can ask is after this interview what would you tell us as a facility?

Thank you

Interview Time: 32 mins 47secs
