# Supplementary material for: Barriers and facilitators of care among visceral leishmaniasis patients following the implementation of a decentralized model in Turkana County, Kenya
Source: PLOS Glob Public Health. 2025 Mar 31;5(3):e0004161. doi: 10.1371/journal.pgph.0004161 (PMC11957299; doi:10.1371/journal.pgph.0004161)
Supplement: S1 Data — This file includes the following transcripts: •VL Patient In-depth Interview Transcripts: Verbatim transcripts of interviews conducted with VL patients, capturing their insights and lived experiences. •Healthcare Worker Key Informant Interview (KII) Transcripts: Transcripts from key informant interviews with healthcare workers, detailing their perspectives on decentralized care models for VL. (ZIP) [file pgph.0004161.s003.zip › HCW and IDI transcripts/healthcare workers/Res 013_FACILITY 1.docx]

VL DECENTRALISED STUDY

KEY INFORMANTS INTERVIEW WITH HEALTHCARE WORKERS

FACILITY 1

**INTERVIEW**
Q1.so we are starting what causes this visceral leishmaniasis?...
RES:mmh it is caused by a sandfly.....

Int: so it is caused by a sandfly

Res: Yes…a sandfly

Int:…okay mmh
Qb.so…how is this Visceral leishmaniasis transmitted from one person to the other?
RES. …mmmh from one person to the other..mmh it actually not transmitted from one person to other but..aah..after the sandfly bites someone...yes..it infects the person...

Int: so it affects the person...okay...
Qc.so which category of individuals is most at risk of VL and why?
RES.so….VL actually affects..aa..people from low socio economic..level..yes. that is..aahh..mostly with low immunity..aa.like children mostly...aaaa... pregnant mothers..and people with old age..and people with other comorbidities like TB and HIV....yeah .
Que: .what are symptoms that patients with VL present to the facility with??
RES.mmmh the first one….. actually a patients present with high fevers..and fevers that are not subsiding..you do amalaria test..aa..it rules out malaria so you start suspecting VL and also when you do...aaa..physical examination on abdomen the patients most likely present with the splenomegaly and at times also with the hepatomegaly....mmh..okay
Que: .on average how long do VL patients in this area take before seeking treatment after developing such symptoms?
RES.:…so initially..ee.. initially..aam...around 2018 to 2000..when I started also doing some management for VL patients were presenting with...ee history of more than three months,four months..aa so that was they were coming when the symptoms were worsening the patients were very sick but now with some awareness that has been done..aaa..aa..through trainings of health care workers and community, CHVs on the awareness of the disease..aaa the at moment currently we usually received patients with the history of two, three months....mmm...
Que: .how do you handle those patients once they present themselves to this facility with the indicated symptoms?
RES.okay..aaa for this patient's actually kalazar we are managing them for free ..aaa..you suspect kalazar case we have ruled out the other condition you do a lab test that's is as we used a rapid test that's is RK39 or DAT or if you suspect a relapse we usually do asplenic aspirates yeah so to arrive at diagnosis and once a patients is diagnosed and we also do other...aa..tests like liver function test just to see if the liver is okay aaa full haemogram just to check if hb is okay.. which is very important and most of them present with low HBs so if patients with low HB we actually boost the HB first before we initiate on medication...yeah (phone rung).
Que:.so what treatment do you offer for VL within this facility???Basing on how currently conduct the VL treatment...
RES.So aam..we usually have..aaa..three categories of drugs we have paramomycin  combined with sodium stiboglucognate and if you aaa missing aaa paramomycin...aaa we usually used sodium stiboglucognate for 28days but if you combine the two you give  for 17days then..we have this special cases like..aa.. children under 2yrs… pregnant women with kalazar, HIV coinfected with kalazar and then cases of relapse that's where we used the second line that's....aaaa... amphotericin B..aaa lyphosomolar amphotericin B and that's we usually give them for a period of 6 to 10 days and that's is ..aaa.. inpatients....
Que:.so what about on currently follow up VL patients after treatment??
RES.a.a..after treatment we usually follow them up and we recommend them to come back after six months...yeah just to check..if..if the...the...the symptoms have actually have elapsed like fever if it has gone then also check if the spleen if it regressing well as going back......those are some of things that we usually check...mmh...
Que: what about….on drug toxicities....we have a question on drug toxicities
RES:…yes...on bdl....postkalazar or leishmaniasis so we usually report if there is any side effects noted during the management like we initiating… especially the amphotericin B we usually do...aa.trial test so we check first so if you see any reactions then we stopped so usually monitor for even drug toxicities...yeah...okay...
Que: .briefly tell me on how they currently conduct VL stock management at this facility?
RES.mmmmmh..okay..i am not a pharmacist..but I know...aa.because I usually go over the drugs for the patients in the ward we usually have the stock...a stock..... intake book...is..is call what... ohh outpatient whatever for management of drugs when you actually pick the drugs you record the name of the patients together with amputes you have taken then you minus …..that's the exercise we are been doing so you are not supposed just to pick the drugs and we usually pick the full dose for the patients...yeah...
Q.briefly tell me how they currently conduct VL data reporting?.
RES: .mmmh for us we have what we call the case management form that's where we usually …..all the patients diagnosed with kalazar we first of all record them on the case management form that's eee actually is the...the.. tool that guides us in the management of kalazar and this is the tool that actually... is needed...in the reporting on DHIS...so once the patients complete that treatment or he is captured in that tool we usually have an HRIO specifically designated for kalazar issues so he enters the information on the DHIS....mmmh....
Que: .Has any member of the community succumbed to the disease??
RES.yeah...mmh not.. kalazar per say...aaa but this are correlated with other comorbidities like TB, patients coming with kalazar aaa with other infections like TB,HIV in the last stages with severe anemia so those complications are ones which are actually kills the patients so not aaa kalazar per say...mmh
Qk.what or which part of VL diagnosis, treatment is most challenging to you?....
RES.okay the most challenging part in this eee VL management..aaa is actually aaa obtaining the samples for splenic aspirate...eh..I know the procedures is so… requires more skills...eh.. that's where the challenge is we don't have that much skills in obtaining that samples of splenic aspirate yeah that's the most challenging management of VL especially for the relapse cases.e..yeah..
Que; .what part of VL diagnosis care and treatment is most enjoyable for you??
RES:….me I really enjoy administering amputation because aaa most people think I am the only person who knows how to do it so I have been doing the all it's in every ward and currently I see every one now is doing it without even our presence there....okay ..mmmh
Que: ….compared to malaria how would you rate the VL burden in this county??
RES.aaaahh..that one can actually be substantiated with the report and aaa....Iam not the... thee...surveillance officer who is able to know the numbers to…aahh…to compare the numbers but currently as we talking now...aaa.. today malaria is on the top of the list..and actually I think this is the season we have many cases of malaria...yeah.. than before..yeah..but VL has also been a burden from the time we started managing them but the cases have been going now down..but for malaria as we are talking today is aburden now in the county..I..I..have we work have new species of malaria I think is from Ethiopia...
Que: .Can you tell me on the relationship between HIV and VL??
RES:….mmmmh..i don't think we have that direct relationship but in most cases...eeh..aah as I said earlier..mmh most cases we usually get VL co-infected with HIV I think is because of low immunity you know VL you can have VL in our body system not unless our immunity has gone down that's the time now the symptoms start appearing so those patients with HIV with low immunity levels or high viral load they usually also present with also kalazar just because of issue of immunity...okay..
Que: .how prepared do you feel to handle the provision of VL services within this facility?
RES:…..No…… I have been handling it since 2018 and I..I.. very much comfortable in handling cases and I think at the moment there is no case of VL that that..has challenge me on handling it except I have not heard a case of PKDL...aaa post kalazar double leishmaniasis that's may be a place where can challenge me because I have not handle one since I came...okay .mmmh
Que: .are you concerned about work demands that may come with managing VL cases in this facility?
RES:.Yes I am concerned......
Que:..on willingness to perform VL screening at part of your work routine.?
RES:….ofcourse we have been doing that....yes..
Que: .on willingness to perform VL diagnosis as part of your work routine??..
RES”…diagnosing VL…..thats also a part of clinician it actually to ensure that we arrive with correct diagnosis so if you a get patients with fevers you have to rule out with malaria and other...aaa...issues then you also go ahead and check for VL if you are highly suspecting VL depending on the signs and symptoms of the patients..
Que: .willingness to perform VL treatment as part of work routine??
RES.yes... you manage after aaa.. diagnosing then you prescribe… you ensure the patients have received the drugs then you also do a follow up...okay....mmmh
Que:….also on stock management?
RES.yes Iam very much concerned because Iam the..the kalazar focal person in the facility so have to check the stock out…stock in and out..eeh
Que:...what about on data reporting..?
RES:….data reporting that is done hundred percent because we have those case management booklets..ehh..in every..eh...unit..eh..so the HRIOs usually passes there every morning to pick any new cases...eh.. that has been reported in reporting tools...so to enter into those DHIS... okay
Que: .has managing VL cases in your facility in any way affected your work schedule or your wellbeing???
RES:...No..yeh...it hasn't..yeh.okay......

Que:.what about on challenges??

RES...yesss....we have had some challenges of stock outs..stock out..yes...stock out.. at times then also have some challenges some patients are willing to be treated as an outpatient and then the place where they reside may be there is no that trained staff who can be able to initiate those medications for VL ….so we also have  challenges to do with the staff knowledge about case management..eeh diagnosing and treatment...so we need may be to have some more trainings ..to...to our staffs..because in recent past we have been having staff turn overs so if you get new staff again we do some wage it is also comes had on our side..
Que: .have you ever received any specific training or skills development related to the provision of VL services?
RES..Yes...I had training once….that was on 2018 on VL case management....okay..
Que: .Do you think that bringing visceral leishmaniasis services to this clinic has in any way affected other services at the facility?.
RES.Not really...mmh.. in fact it has helped because in most cases some patients were having VL but it were being overlooked but currently patients with VL are managed well and..aaaa....after receiving the correct dose and number of days they are discharged with good health...
Que: .what does the community say about VL and what is the impact of such perceptions on care seeking?
RES:.aahh initially.. initially..the community did not have that enough knowledge about  VL...they used to call it 'etid' yeh..so they usually performed some aaa …surgical traditional procedures at home so most patient's infact come with those all therapeutic marks at their abdomen so they though may be the spleens has enlarge so by cutting those...aaaa..abdomen you would be  reducing the infections so would be making the infections to come out... but currently with the awareness the health seeking behavior with the kalazar issues or VL is actually on top..the patients are coming as I said patients are coming as early as one month seeking for… even to be tested for kalazar if they feel the spleen is enlarged they also relate it to VL..yeah currently..mmmh..but initially it wasn't the case...mmh..okay
Que:...so if we were to roll out VL diagnosis care and management programs to other health facilities what areas would you recommend we improve?
RES:..so..for us or you...to roll out VL management in other areas especially in remote areas first of all we need to sensitized the community...that's the first thing.. sensitization...yes the community should be sensitized about the disease and to improve on aaaa...health seeking behavior about the same then also conduct a training atleast  one week training for all health workers who will be managing those patients and also ensure if there is enough stock in the the the county so that we are able to stock those facilities with the VL commodities...mmh..yeh... because aah..from the past we have been having challenges of shortages even the RK39 has been having issues… shortage and also kalazar drugs we have been having run out stock at point of time.
Que:...whom do you think should be trained at the community level to improve health seeking behavior for VL patients???
RES.CHVs and also health care workers at that communities..mmh..

okay thank for your participation.

Thank you.
