# Supplementary material for: Barriers and facilitators of care among visceral leishmaniasis patients following the implementation of a decentralized model in Turkana County, Kenya
Source: PLOS Glob Public Health. 2025 Mar 31;5(3):e0004161. doi: 10.1371/journal.pgph.0004161 (PMC11957299; doi:10.1371/journal.pgph.0004161)
Supplement: S1 Data — This file includes the following transcripts: •VL Patient In-depth Interview Transcripts: Verbatim transcripts of interviews conducted with VL patients, capturing their insights and lived experiences. •Healthcare Worker Key Informant Interview (KII) Transcripts: Transcripts from key informant interviews with healthcare workers, detailing their perspectives on decentralized care models for VL. (ZIP) [file pgph.0004161.s003.zip › HCW and IDI transcripts/healthcare workers/Res 014_FACILITY 1.docx]

VL DECENTRALIZED STUDY

HEALTH CARE WORKER

**INTERVIEW**

Que: What do you understand abou Kalazar?

Res:,,,, Kalazar,,,Kalazar is a disease caused "byyyyy" Sandflies.

Que:"mmmh " "cleaning noises" How does it transmit?

Res: through bites.

Que: Which kind of bite? Is it sandfly bite or..

Res: Sandflies bites.

Que:Which category of individual is most at Risk of getting Kalazar?

Res: People who are,,,,,Herdsmen. People with.....

Que: People with?

Res: People live in forest, who take care of Cows and the Animals

Que:"yeah "

Res:"mmmh "

Que: What made them affected by Kalazar?

Res: Because in the,,, Where is the anthill, in the morning the sandfly comes out and the move to the forest, and when man is found in the forest, He or She bitten by that sandfly.

Que: "eeeh" what are the symptoms that the patients with VL present to the facility?

Res:,,,, "nurses walking noise" Patients may have wastage,,,, paleness of the eyes and the nails,palms… there is hotness of the body, causing fever which is not subsiding after treatment. Some of them have been treated of Malaria at Health centres. "Patient walk in" "kidogo mama utazaidiwa na huyo",,,,,,,,,,When you pipette there with spinomegalin naed hepatomegally,

Int: Abdominal swelling.

Que: On average how long do VL patients in this area took before seeking for treatment?

Res: "door bang" They take long, almost….More than one month. Some of them have been treated gone home, gone to the medicine they said medicine man, They treat with herbal medicine they tattoo there stomach and giving herbs products. They take even a month, some comes after one month, some 2 months, after they have been tattooed, they have lost a lot of blood. That when person see, He supposed to seek treatment at Hospital is when they come.

Que: So it is that stomach tattooing that made them delay of seeking treatment at Hospital or is there anything else that made them not to seek treatment at Facility earlier?

Res: They believe that it is a curse. Most of them used to be treated by those medicine men.

Que:How do you handle patients once they present to the facility with the indicated symptoms.

Res: ,,,,,,"patients noise" We normally take basics "eeeh" investigations like Malaria, all conditions like Malaria…like wastage some of them come with coughing...we take chest X-ray to rule out TB, we also take Kalazar test, after they confirmed, Is when we do harmogram, If the HB is low, you know we cannot start drugs when the HB is below 7.5 "child crying " we have to transfuse first then we start drugs.

Que: "mmmh " and how do you offer treatment for VL within the facility?

Res: We normally order from the pharmacy, when the patients is confirmed Kalazar positive.

Que: yes

Res: We normally order drugs when the HB's is above 7.5, The CO or MO writes a form. There is form for specific for Kalazar patients, We fill that form, we take Age, Height, After that is when we go with the file to collect drugs from the pharmacy.

Que: oh Okay

Res: After the patient have been confirmed with Liver function and the Kidney function, there is a person who is in charge of Kalazar "they are the once who come to the ward confirmed if the patient has No any other problem like kidney disease, Hepatitis B,TB . We have specific drugs according to the condition of the patient. Those with Kidney disease, Hepatitis B and TB we give antisom and those lactating mothers. But those without those conditions we give paramycin endosperm.

Que: How do you conduct VL treatment and data reports here?

Res: …We normally have the records for the,, for Kalazar patients, when we are given those drugs, they are only specific for one patient, If your have 2 patients you calculate the dosage for 17 days, like SSG paranomycin.. you give for 17 days ….For ambisom you have those for early dose we give for 10 days while those with low dosage, For 6 days. Every patient should have full dose before we start.

Que: Do you usually follow up the after treatment?

Res: yes, we normally refer them to the nearest Health centre. Because most of them come from far… and why do they referred is because maybe the patient is anaemic they cannot transfused, they have referred to us. after transfusion we start drugs, we check after 3 days HB then you continue with the treatment for 17 days. Others for 10 days for ambisom then we recheck the HB …haemoglobin level if it is low we don't discharge, if it is high we give him atenix then we refer them to the nearest hospital in case of anything. But they have to come back after one month if they are healthy.

Que: "oh" okay, Then is there any drug toxicities cases?

Res: Yes, We have some patients who normally react with the drugs, then we stop ..we have to stop and then check what is wrong.

Que: What made them react with drugs?

Res: "mmmh ?"

Que: What made them react with drugs?

Res: We don't know. because they are not exposed to those drugs. We know those drugs are toxicities.

Que: Has any member of the community succumbed to the disease? In terms of mortality

Res: There are some, ….some normally succumbed when maybe we don't have blood, like Maybe you don't have blood that matches with the patient, they normally die. And those who come late ….like those with other conditions like TB, Hepatitis B,. We normally give them drugs but they don't ….(INAUDIBLE)

Que: What part of VL diagnosis, treatment is most challenging?

Res: What part?

Que: I mean what of VL or Kalazar, diagnosis, treatment is most challenging?

Res: Part of Kalazar?

Que:Yes

Res: We have only one type

Que: Which type?

Res: Visceral leishmaniasis

Que: That one, which part of it is most challenging in treatment and also diagnosis?

Res: …Most of them come late, We get them late, they start late treatment, when they are already wasted and cannot withstand the drugs, Some of them if we don't ..they don’t diagnose earlier with other conditions, they normally die because of anaemia, maybe because of TB.

Que: Oh okay.

Res: Some of them nose bleeding, they complicate …they start nose bleeding, diarrhoea….

Que: What part of VL diagnosis, care and treatment is most enjoyable for you?

Res: We enjoy?

Que: Yes

Res: When the patient is improved. Some of them even improve very well after giving only for 5 days, you see a patients have improved even when he has any other …any other condition.

Que: Compared to Malaria, how would you rate VL burden in the facility,county level

Res: Rate how?

Que: Comparing Malaria and VL, explain how will you rate.

Res: VL is more complicated than Malaria,

Que: So VL is more complicated?

Res: Sometimes you can start treatment the patients complicates then you stop,… you do not continue maybe it is anaemia setting in or the blood has gone low to 5grams..per day. You have to stop the treatment and then you treat that anaemia, then start again. But Malaria if you give the correct drugs at the required time, the patients will improve faster,,,,,, "mmmh " Now Kalazar patient stay longer in the Hospital maybe you don't have blood, the patient has come . The patient will not get the treatment and that is the longer period maybe 1 week, 2 weeks,,,,,"mmm" then after transfusion is when you start 17 days again….it takes time

Que:Okay

Res:"Mmmh"

Que: How does VL relate with HIV? Does they relate?

Res:,,,,,, mmmh if the patients is wasted most of people may think, those who Don't understand Kalazar well but for us we have dealt with this patients for long time so we know, we have to check some parameters so as to diagnosed Kalazar.

Que: So there is no relationship between HIV and Kalazar?

Res: Not related.

Que: okay. How prepared do you feel to handle the provision of VL services within this facility?

Res: How do?

Que: How prepared do you feel to handle the provision of VL services within this facility?

Res: We are ready to because, we have drugs available, our lab is able to do Kalazar test and test all parameters that are related to Kalazar… like liver function and kidney functions test. ….Our staffs are dedicated on how to administer the drugs,… our nutritionist are available. They normally come in the morning to look for patients, take report and provide them in the required nutrition supplement.

Que: Are you concerned about work demands that may come with managing VL cases in your facility? I mean in terms of willingness of VL screening and diagnosis

Res: Yes we are willing, Most of the patients suffer because they don't know what is happening unless they are screened earlier, you cannot be able to manage them. If they come late we will lose them.

Que: Has managing VL cases in this facility in any way affected your work schedule?

Res: No

Que: Even your wellbeing?

Res: It normally affects us when,,,,maybe the patients dies, you know if the patients die and maybe there is No blood, you feel it. As a human being you don't want to lose them, we feel that we have lost and we could have helped, but it is not our mistake.

Que: is there any challenges you faced while managing VL?

Res: challenges, we have challenges…..Some of our patients, you know it is their first time maybe they are believing it is a cursed. They are Some patients who come here, they don't want drugs. There is a lady here 16years old girl, she was crying "I don't want this drug, I don't want this,,,because she was not used to, she has not been treated before and she is coming. Some of them even abscond. They don’t want to be treated or maybe he sees another patient die, she runs away without treatment drugs. How to follow them you don't know maybe she as gone home or she as not gone home. Maybe the number you are given in the,,, as the next of kin. If you call nobody takes. You know that is a challenge to us. The patients as already received two do ses of ambisom treatment and she runs away…if you call. …If you call that number nobody responded

Que: Have you received any specific training or skill development related to the provision of VL services?

Res: Have not received gone for any training for Kalazar

Que: Even the colleagues?

Res: Maybe,,,, Only JL is the one comes here and tell us on new treatment on new management but we have not been taken to any,,,, maybe "chini ya maji" through back door.

Que: Did he share his experience with you?

Res: Sharing?

Que: I mean did JL share his experience withh you?

Res: He normally shares like the dosage of ambisom you cannot manage alone. You to at least, like me now I have served for almost 3 years. I'm able to calculate ambisom, but we have some staffs,,, we need to teach them and even when we are defeated with the calculation, we normally call JL or W to help us in calculations

Que: Have you received more resources eg. Personnel/equipment to help you manage VL cases following decentralisation of VL care in the county

Res: Resources?

Que: "yeah"

Res:Like what? Medicine

Que: "yeah" Medicine.

Res: We Normally,,,Sometimes we run short of drugs like the paranomycin. We are given the one for 2 units when it falls down, you are calculated maybe 13 exactly. When one falls down or 2 that is a lost. We have to go again to search for another one, that is the problem we are having.

Que: Do you think that bringing VL services to this clinic has in any way affected other services at the facility?

Res: No,,….

Que: What does the community say about VL?

Res: They believe it is a curse, they have been bewitched, some of them even say discharge us so that we can… can go home and do "kienyieji" Local treatment. If you explained to them that you are starting today drugs then you go for 17 days after that we check HB, they say that is alot of time. Better we discharge him and go home but there is … when we call for those counsellors to come and help us.

Que: What are the impact of such perception on care seeking?

Res: Impact?

Que:"mmmmh "

Res: Maybe they don't information on such diseases, mobilisation has not been done well maybe "Nurse talking " ….Inform in the villages we need more information to reach to those area.

Que: Okay, If were to roll out Diagnosis, care and management programs to other health facilities, what areas would you recommend we improve?

Res: Area?

Que: Areas that we should improve in VL?

Res: VL,,,, Diagnosis, in diagnosis

Que: Diagnosis?

Res: "mmh" we were told there is kit for testing Kalazar, it requires 6 patients. Is that okay?

Que: "mmmh"

Res: Now If we have 1 Kalazar patient or 2, we wait for more to come? We need the test to be improved so that if the patient, ….the first patient comes is tested …another one comes we tested, so if you wait for those 6 "laughter "it makes our diagnosis to delay that is what we are requesting because it was in August we had that challenge for testing patients they said until on Friday when the patients will be 6 to be tested.

Que: Whom do you think should be trained at the community level to improve health seeking behaviour for VL patients?

Res: "mmmh " the community health workers and the nurses.

Que: Why them?

Res: Because testing should be done at least house to house. And the Education should start from homes. Because if you wait for patients to come to the facility, the message won't reach the homes, some of them maybe they are in the house,,they are very sick and they are not coming out to the hospital so…so we need to empower those community health workers so that they can do at least house to house and check and tell patients if you see this and this take the patients to the hospital to be investigated maybe it is this or Malaria or Kalazar. So that patients can come early and our mortality rate may reduce.

Que:Okay.

Res: "yeah "

Que: I think We have come to an end of our interview do you have any question for me?

Res: We need training at least for our staffs, some,,,,sometimes you are,,,maybe

you are very busy, time of the training or dedicating another person to,,, on Kalazar, you Don't have time, so it is you alone who can give drugs because the other doesn't know, and if you go away, that drug won't be administered well as supposed to be. So we need more training so that our nurses can know, can get the information well. You know even us as nurses we are the ones who deal with patients, maybe the patient have come on Friday. On Friday the M.O's only work, come for few hours and goes away, you are the one who supposed to know what is going on with the patients, you can also diagnose. Even Nurse we normally said….that this patient you treated for malaria…. what is wrong with this patients we treated for Malaria but this patients is not improving,, what is wrong, Maybe we have missed up something. We normally even diagnose and go for testing, they get the patients is positive. It is better train us in that they can also make a diagnosis where the other person as not made it you can do it to make sure the patient get the right treatment and improve.

Que: Noted and Thanks for participating.

Time: 28 minutes and 14 seconds.
