# Supplementary material for: Barriers and facilitators of care among visceral leishmaniasis patients following the implementation of a decentralized model in Turkana County, Kenya
Source: PLOS Glob Public Health. 2025 Mar 31;5(3):e0004161. doi: 10.1371/journal.pgph.0004161 (PMC11957299; doi:10.1371/journal.pgph.0004161)
Supplement: S1 Data — This file includes the following transcripts: •VL Patient In-depth Interview Transcripts: Verbatim transcripts of interviews conducted with VL patients, capturing their insights and lived experiences. •Healthcare Worker Key Informant Interview (KII) Transcripts: Transcripts from key informant interviews with healthcare workers, detailing their perspectives on decentralized care models for VL. (ZIP) [file pgph.0004161.s003.zip › HCW and IDI transcripts/healthcare workers/Res 015_FACILITY 1.docx]

VL DECENTRALISED STUDY

HEALTHCARE WORKER KEY INFORMANT INTERVIEW

Que: Okay…What causes Kala-Zar?

Ans: Kala-zar is caused by the sandfly that contains leshmania donovani cause it always bites the host and cause kala-azar

Que: How is VL transmitted from one person to another?

Ans: Visceral leishmaniasis is transmitted from one person to the other …..whereby the sandfly bites the infected person and again goes and bites an uninfected person so the other person tends to get infected with VL

Que: Which category of individuals is most at risk of VL and why?

Ans: I can say children, the elderly, and the immunosuppressed

Que: What are the symptoms that patients with VL Present to the facility with?

Ans: They usually…the sort of symptoms that are more specific that the children….that the persons with Klazar present with are distended abdomen, fever, history of non-intended weightloss and anaemia

Que: On average how long do VL patients in this area take before seeking treatment after developing symptoms?

Ans: I think they take aaaa…longer time before they seek treatment because most of them live in a distance place whereby they don’t tend to get to the facility as early as possible

Que

Ans: Okay…we first manage the symptoms..if its anaemia we correct anaemia…we transfuse we give hermatinnics ..If its distended abdomen we make sure that the patient is comfortable …if its…aaaahh ….fever we manage fever then we start the investigation if its positive we start on the management

Que

Ans: mmmhh….I think we always give aahh paramomycin for I think 17 days and we have aaahh …Liposomal amphotericin B..we can also give that..amphotericin

Que: Questions are now on how you conduct VL treatment?

Res: I think eehh…it’s just the same thing I have said you give aaahh…the IV medication, the anti kala-azar for 17 days

Que: How do you conduct follow-up of VL patients after treatment?

Ans: there also is a followed up by the anti kala-azar team and the community health workers I think

Que: What of the major drug toxicities…question on major drug toxicities

Res: I think nephrotoxicity and alsoooooo……..nephrotoxicity is the only one and then while managing you can also have electrolyte imbalance which is usually often…you will always get them

Que: how do you conduct VL stock management.

Ans: Stock management is done by the anti-kalazar team and the pharmtecs I think…the pharmacists

Que: What of data reporting?

Res: mmmmmh…the anti kala-azar team I think they do that and the casualty also

Que: Has any member of the community succumbed to the disease?

Res: Our community has medical practitioners is aaaahh……the person staying in the hospital are part of our community so I think patients are part of our community…so ..yeah we have lost lives

Que: What part of VL Diagnosis, treatment is most challenging for you?

Ans: The microscopic part and the PCR part is not always clear

Que: What part of VL diagnosis, care and treatment is most enjoyable for you?

Ans: mmhhhhh the rapid diagnostic tests

Que: Can you tell me about HIV and VL? The relationship between HIV and VL

Ans: there is some sort of immune-pathological pathway that enhances the replication of both the VL and the HIV viruses so there is a relationship between HIV and VL

Que: Compared to malaria, how do you rate VL burden in the county?

Ans: Malaria is rampant you cannot compare it with visceral leishmaniasis….we still have a burden with malaria

Que: How prepared do you feel to handle the provision of VL services within this facility?

Ans: Averagely prepared

Que: Are you concerned about work demands that may come with managing VL cases in your facility?

Ans: yes…VL diagnosis is not in my work length so I will leave it to the technicians

Que: Has managing VL cases in your facility in any way affected your work schedule or your wellbeing

Res: Not at all because it’s part of my work to see all patients in my ward

Que: Have received any specific training or skill development related to the provision of VL services?

Ans: Not yet so far

Que: Have you received more resources e.g., personnel/equipment to help you manage VL cases following decentralization of VL care in the County?

Ans: Yes we have the anti-kala zar team ….they are always here…we consult them…they teach us on what to do and they explain some sort of things….when things are not clear

Que: Do you think that bringing Visceral leishmaniasis services to this clinic has in any way affected other services at the facility?

Ans: Not at all

Que: What does the community say about VL and what is the impact of such perceptions on care seeking?

Ans: I think the community does not know..look at kala-azar as…like other communicable diseases cause they tend to see that kala-azar maybe as at some point it affects some people with coincidentally maybe they affect the immunosuppressed so they tend to think like kala-azar is for the HIV positive patients, the malnourished or some sought of thing…they don’t understand that it’s just a communicable disease

Que: If we were to roll out VL Diagnosis, care and management programs to other health facilities, what areas would you recommend we improve.

Ans: mmmmhhhh…. I think we should improve on training of the human resource also resources like medication, diagnostic kits yeah…. and education to the community at large

Que: Whom do you think should be trained at the community level to improve health-seeking behaviour for VL patients?

The leaders of the family, the controller of the homes and the households which are the mothers, I think when you educate a woman you just give sort of impact in decision making fast. Mothers first, CHVs, village elders, the gate openers in the community.

I am done unless you have any questions for me.

Thank you I don’t have any question.

Thank you.

Interview time: 09.07
