# Supplementary material for: Barriers and facilitators of care among visceral leishmaniasis patients following the implementation of a decentralized model in Turkana County, Kenya
Source: PLOS Glob Public Health. 2025 Mar 31;5(3):e0004161. doi: 10.1371/journal.pgph.0004161 (PMC11957299; doi:10.1371/journal.pgph.0004161)
Supplement: S1 Data — This file includes the following transcripts: •VL Patient In-depth Interview Transcripts: Verbatim transcripts of interviews conducted with VL patients, capturing their insights and lived experiences. •Healthcare Worker Key Informant Interview (KII) Transcripts: Transcripts from key informant interviews with healthcare workers, detailing their perspectives on decentralized care models for VL. (ZIP) [file pgph.0004161.s003.zip › HCW and IDI transcripts/healthcare workers/Res 10_ FACILITY 4.docx]

VL DECENTRALISED STUDY

HEALTH CARE WORKER

**INTERVIEW**

Que: What do you understand about VL/Kalazar in the area?,,,, I mean what are the causes, symptoms. Just explain how do you understand more about Kalazar.

Res: According to me and the client that usually see coming to this facility, one you must identify this client by the look of his eyes. You might get the eyes are pale that show he is lacking ,,,,he is lacking some,,, maybe we say that is anaemic. Another you might get a Child with a very big stomach.

Que:Yes

Res: If you take that Child to the Laboratory maybe you can get Child He is having Kalazar.

Que:So those are the symptoms?

Res:"yeah"

Que: What are the causes of Kalazar?

Res:I think one it might be lack of iron …so food in general.

Que: How does it transmit from one person to the other?

Res: Transmit from one person to the other one,,,,,,It think through cough, can it be through cough...or sharing

Que: yes

Res: because you might get one family might be having 2 children having Kalazar. But if you really try to follow

Que: yes

Res: now the cause of transmission, me I believe because of lack of food because of ….Malnutrition

Que: yes, You mean that the is cause?

Res: yeah"

Que: So lack of food is the main cause of disease transmission

Res: "yeah"

Que: And Which category of individual is most at Risk of VL?

Res: Majority they are Under 5 years.

Que: Why under 5 years?

Res: Because their immunity….nutrition is low. ….also they cannot struggle to go and look for food. At least this elders they can have bite from different places, especially this one under 5 years, maybe because of immune system or their immune system are not very strong.

Que:Which area are mostly burdened by this…prone to this disease?

Res: Areas?

Que: yes

Res:Mostly they are here Turkana West and then Nadapal. Most cases are from Nadapal.

Que:"mmmh"

Res:"mmmh"

Que: Why does it affect Nadapal people mostly?

Res: Nadapal now, you see Nadapal is the border between Kenya and Sudan

Que:"eeh"

Res: I don't know what is the problem there I cannot answer that one "laughter "

Que: It seems the disease came from neighbouring country or they transmitted from themselves?

Res: I think maybe they transmitted themselves from neighbouring country, because majority come from Nadapal are…the mostly affected

Que: Okay

Res: "mmmh"

Que: What are the symptoms that patients with VL present to the facility?

Res: Symptoms?

Que: "yeah"

Res: Maybe you will get a person with fever

Que:"mmmh "

Res: Red eyes

Que:"eeeh"

Res: Then Complain of stomach pain

Que: yes "birds sounds "

Res: Maybe pale the pla….,,, is very pale

Que: In terms of colour or what ?

Res: Colour.

Que: Skin changes?

Res: Skin changed.

Que:Okay,,,,,,,,,,,, On average

Res:"mmmh "

Que: How long do VL patients in this area take before seeking treatment?

Res: So it depends, you see like Nadapal some of this clients because of lack of security

Que:"mmmh "

Res: It forcing them to look for GSU vehicle at least they see the GSU vehicles coming this side of Lokichoggio it is when so that they can use that vehicles to this sides

Que: "eeh"

Res: Because of security so it gave somebody can stay at home for those 5 Months not being treated, 2 months 3 Months like that.

Que: So it is only insecurity that causes them not to get to facility at the right time or what?

Res: One it is insecurity

Que:"mmh"

Res: Two, it might be,,,,, there is also alot of ignorance from parents

Que:"mmmh "

Res: Because the child is complaining of being sick but they are not serious of

taking care of the child and 3. It might be availability for transport. Actually he might get the facility is very far, because for example here Lopiding it serves upto Nanaam. You get a person from Nanaam coming to this place it is also a far distance

Que: Okay. And how do you handle the patients once they present to the facility with the indicated symptoms?

Res: Immediately when the,,, reach to the facility is being seeing by the clinician, after presenting all those signs, they will sent to the Laboratory for the test

Que:,,,,,,,,"bird noise" Okay, What kind of treatment do you offer for VL within the facility?

Res: The facility have treatment

Que: mmmh "

Res: SSG, stibo and we have paranomycin so when you used stibo or SSG

Que:"eeh"

Res: That treatment usually goes for 30 days if there is No paranomiycin. But if you have Paranomycin you shorten that period of treatment for 17 days.We have paranomycin and SSG. We have another new one that have come ambisom.

Que:"eeh"

Res:"mmmh "

Que:Okay, Do you usually follow up VL patients after treatment?

Res: We usually follow, maybe they will tell after one month they come back for review

Que:Okay

Res:"mmmh "

Que: Every month?

Res: Every month.

Que: Okay, Is.....is there any toxicities of drugs cases that you have heard from the patients?

Res:No. I think all of our patients were being treated and they do well. Not received any complain about drugs

Que: So drugs are good they don't affect them?

Res: yes.

Que: That is good.

Res: "mmmh".

Que: How do you conduct the stock management in the facility?

Res: Stock management usually control through bill card and also by the number of clients that we usually receive per month, that one call tell us how much drugs we can order next month.

Que: Mmh"

Res:"yeah"

Que: How about data reporting, how do you conduct it?

Res: Data reporting now, I cannot answer that one because that one can be answered by HR

Que: "mmmh"

Res: "yeah"

Que: Has any member of the community succumbed to the disease?

Res: Within the community?

Que: yeah within the community

Res: We have.

Que: Why do they succumbed?

Res: There is an area here

Que: yes

Res: I Don't know that name,,,,,,there is an area down here, I don't remember the name of that place.

Que:"mmmh "

Res: "mmh" actually that person didn't reach the facility

Que:"mmmh "

Res: So the reason he didn't reach the facility at the right time?

Res: He never reach the facility at the right time, He reach at the facility when he is too late

Que: Okay, What part of VL diagnosis, treatment is most challenging for you?

Res: can you repeat it again.

Que: What part Of VL diagnosis, treatment is most challenging for you? Most challenging part when undertaking VL management.

Res: There is no place that is very challenging …., as long we have the drugs available

Que: Yes

Red: I think everything is easy once you have been diagnosed, you will be treated.

Que: Okay, Is there any part of VL diagnosis, care and treatment is most enjoyable while working?,,,,,What is enjoying part of it?

Res: Enjoy part of it,,,,,,,, part of the treatment. Maybe to be enjoying if you treat your patient and your patient heal, that you can enjoy but you can treat a person and the person pass away you won't enjoy.

Que: "Oh" okay.

Res:"mmmh "

Que: Compared to Malaria, how would you rate VL burden in the facility?

Res: Malaria?

Que: yes

Res: Malaria like right now I can say this is the season of Malaria in Turkana West right now,,,,,,

Que: So Malaria is the toughest one then VL?

Res: Malaria is the toughest one than Kalazar because Kalazar cannot take you very first like Malaria.

Que: Okay, so Malaria is not curable or what?

Res: Malaria is curable.

Que: And why is it that burden than Kalazar?

Res: You Malaria

Que:"mmmh "

Res: Once you have been diagnosed Malaria because parents from this place once their child have just collapse down, they know that is Malaria, they rush to Hospital and get treated for …….for Kalazar it usually takes time to show the signs and symptoms

Que:"eeh"

Res: so Once the child complaining, they just buy Panadols from the Bush their or from chemistry, flagyl take this one take this one "cough" until the situation gets worse and worse and worse

Que: "Mmmh" it is okay. Is there any relationship between Kalazar and HIV?,,,,,,,How does it relate, do they relate?

Res:,,,, I cannot say but I have tried to see about 3 client from HIV and Kalazar and I was also supposed to learn to know how is it related, HIV and Kalazar but so far I'm still being there.

Que: How prepared do you feel to handle the provision of VL services within this facility?

Res: professional?

Que: I mean what has made provision of VL care easier? ,,,you as the health worker what part of VL made your work more easier?

Res: If you want to feel, maybe so long you have all what you required in the facility you will feel. Like reagent in the Laboratory, somebody comes you sent to the Laboratory, get the reagent I think that is good you feel good. In terms of treatment you have stock that will be required for that person

Que: oh Okay, are you concerned about work demands that may come with

managing VL cases in your facility?,,,,,,,,, Screening part, diagnosis part of work routine are you concern of any?

Res: We usually concerned with diagnosis, especially those client of Kalazar and maybe 2 those I saw with sick lungs

Que: Has managing VL cases in your facility in any way affected your work schedule or your wellbeing?

Res: I think our work is being scheduled

Que: Does it affect you due to more workload?

Res: Workload because there is alot of shortage of staff I think that is more workload.

Que: Okay, Does it affected you? And what challenges do you feel.

Res: It affects because of over strain

Que: "mmmh "

Res: There is place where you're supposed to work like about 2 but you work one person, you rush there, You rush there..

Que: And have you received any specific training or skill development related to the provision of VL services?

Res: No.

Que: Even your colleagues and friends?

Res: Maybe my colleague.

Que: Do they share there experience with you?

Res: The problem is if somebody goes for training coming there he keep quite, but none of our colleagues have shared with us about the management of this Kalazar whe she or he goes for a training. Their is only one training for ambisom that managing of Kalazar, my Colleagues went, He came back and He told us one day that we will have training about ambisom but upto date He is on his leave maybe until he comes back

Res: Oh okay, Have you received more resources to help you manage VL cases following decentralisation of VL care in the county?

Res: We have received resources it was because of the county could not be moving any far, we do received resources

Que: You get resources from the county or also from other donors?

Res: county and good partners we have a partner here called NSSF he used to support us on management of Kalazar so much, right now NSSF have moved on to Sudan

Que:"mmmh " Okay. Do you think that bringing VL services to this facility has in any way affected other services?

Res: Bringing?

Que: Do you think that bringing VL services to this clinic has in any was affected other services at the facility?

Res: It will not affect.

Que: Okay.

Res:"mmmh "

Que: What does the community say about the VL ?

Res: I think the community is too happy and they appreciate for the service or the management they usually receive when they arrived at the facility.

Que: Okay.,,,,, If we were to roll out VL diagnosis, care and management programs to other health facilities, what areas would you recommend we improve?

Res: the area ah...we suppose to improve maybe having trainings for staffs

Que:"eeh"

Res: We ensure enough stocks for management of this Kalazar and Malaria

Que:"mmmh "

Res:"yeah"

Que: Whom do you think should be trained at the community level to improve health seeking behaviour for VL patients?

Res: I think from the community we need to start with the CHV's then you come to the clinicians, Nurses and somebody who is in the pharmacy

Que: Okay.

Que: I think we come to the end of our interview "mmmh" is there any question or any issue you would like to share with me?

Res: ,,,,, me I don't have any question, but I appreciate you for coming to talk to us about management of Kalazar or interview about Kalazar maybe what only like you to say, wish you to support us at any given time that we raise an alarm of certain drug, kindly be supportive, like today we are not at stock out, we have any drug, we have all drugs of Kalazar at any case even if it is a Child any age, we can manage right now,

we are stock with Kalazar drugs right now

Time: 19min22sec.
