# Supplementary material for: Barriers and facilitators of care among visceral leishmaniasis patients following the implementation of a decentralized model in Turkana County, Kenya
Source: PLOS Glob Public Health. 2025 Mar 31;5(3):e0004161. doi: 10.1371/journal.pgph.0004161 (PMC11957299; doi:10.1371/journal.pgph.0004161)
Supplement: S1 Data — This file includes the following transcripts: •VL Patient In-depth Interview Transcripts: Verbatim transcripts of interviews conducted with VL patients, capturing their insights and lived experiences. •Healthcare Worker Key Informant Interview (KII) Transcripts: Transcripts from key informant interviews with healthcare workers, detailing their perspectives on decentralized care models for VL. (ZIP) [file pgph.0004161.s003.zip › HCW and IDI transcripts/healthcare workers/Res 16_FACILITY 1.docx]

VL DECENTRALIZED STUDY

HEALTHCARE WORKER INTERVIEW

**INTERVIEW**
QUE 1: I want to ask you on your knowledge of VL
a)What causes Kala Azar?
RES:Kala azar is caused by a bite of a sandfly that harbors the certain agent of kala Azar.

b)How is VL transmitted from one person to the other?
RES:VL actually is not transmitted from one person to the other but it's through the bite of a sandfly.
(Paper rustles)
c) Which category of individuals is most at risk of VL?
RES:''aaah' we have majorly men because of their lifestyle and their level of exposure,through cattle herding that really exposes them to the bite of a sandfly compared to women that are always found at home where you are not able to be exposed to the bite through aah' that are residing within aah' termites….anthills

Que: Which area do you think is most prone to Kalaazar?
Res: Within Turkana, Kala Azar so far is in Loima sub county,Turkana West,aah' and some parts of Turkana North and Kibish sub county.But majorly Turkana West and Loima.

d)What are the symptoms that patients with VL present to the facility with?
RES: One of the major symptom is fever with more than 14 days ,then we have got aaahhh spinomegali sometimes we also have the epatomegali.And we also have headache,we have a wastage,and sometimes also others have nosebleeding because of oxystasis…..Sometimes also they could be liver denopathy……

e)On average how long do VL patients in this area take before seeking treatment after developing symptoms?
RES:On average aah'maybe a month or two because aah' the health seeking behavior is different from one area to another others will want to wait and see whether they can have some cultural remedies to their situation when the remedies did not work that's when they will seek medical attention in the nearby facilities.
So the issue of distance too it is another factor that aah' that determine how fast they seek for medical attention.

Que: what about the cost of treatment?
The cost of treatment for kala Azar is free however now the issue of distance from where people are seeking treatment is another challenge.So the transport,I mean  the kilometers take how much they move from one locality to another where they are going to get the diagnosis and ofcourse the treatment so the cost is based on where they are coming from but not treatment, treatment is free.

f)How do you handle patients once they present to the facility with the indicated symptoms?
RES: aah'ideally they come through the outpatient department ,they go through triaging and after triaging they are able to meet the clinical officer,when now they present thee,,I mean how they feel like they present to that to the clinical officer who does his or her part and then now they are sent to the lab for testing.After testing the results are sent back to the same clinical officer for review and whether the patient is really kala Azar patient or not.If not a kala Azar patient, maybe now they will have to look at variatial diagnosis ,aah' if the case is not a kala Azar case.

g)What treatment do you offer for Kala Azar patient within this facility.
RES:So in this facility,we are using the national algorithm ,the treatment that we have is ,we have one that goes for 28 days that is the Sodium stibo Gluconate or we call them antimonials,then the second option is a the use of sodium stibo Gluconate plus Paramomycin which goes for 17 days.And now the third option,is those patient that have got aah' HIV co infection or are under severe malnutrition.So those ones are normally are Initiated on what we call aah' ambysom treatment for a period of 6 days.And that is done until now the in patient facility where there is no inpatient they are admitted and began on aaah'on the that, and above all ,for all these treatment we also,we also need to check on haemoglobin level.So the haemoglobin level is within the normal we initiate,if it's not within the normal if it's below,the normal ranges of the patient you first have to manage to come to a normal level before now we initiate the patient on treatment.

*How do you currently follow up on kala Azar patients after treatment?
Res: Follow up is then I believe after six months of treatment.There is always a challenge because sometimes aah' locating these patients is another issue after treatment.We only get to know them when they come back presenting same or other symptoms but mainly because of the ease we take in getting to know they are kala Azar patients,we know have to ask whether they have completed their treatment or not.But mostly we,in other cases or locality,we tell them not to ,for mostly those who are coming from aah'the pastrolists areas they dont have to go and herd their cattles,they have to , after the treatment just stay at home not to  lived back to go herd their cattle because of the risk of exposure to a sandfly bite.

*How do you describe on major drug toxicities?
RES:yeah all, actually kala Azar drugs especially the antimonials,the Sodium stibo Gluconate have very high toxicities.They are highly toxic.They are really toxic so especially sodium stibo Gluconate is very toxic,is very toxic aah' compared to the other one,compared to ambysom."

h)How do you conduct VL stock management?
RES: VL stock management Is aah',as a whole there is a ,there are many levels begining from the NTD coordinator,we normally have stock cards,we only have stock cards that whenever we receive the VL commodity we normally have a stock card for each commodity Beginning from the testing kits and also the drugs.And at the end of every month ,there is a report that is normally done especially for the lab , for the testing kits and also for  the pharmacist ,the pharmacy department they have their own stock cards for all the drugs that are available for treatment and these reports are normally done at the end of the month.They are sent to the NTD coordinator to aggregate and send to the national party for VL.
"Patient  voices and CPU noises"..

I) How do you currently conduct VL data reporting
RES: Already answered in response (h)

j) Has any member of the community succumbed to the disease?
RES : yeah we've had some case aarh' and these are majorly due to aarh' deaths secondly to anaemia.
I cannot get the number exactly but I know of some cases that have succumbed due to anaemia.The number of cases are  normally found aarh' based on the data that we normally send at the end of the month to the NTD coordinator that shares the same same report with the VL national platform for decision making.

k) What part of VL diagnosis,treatment is most challenging For you.
RES: Aaah'..the most challenging for me,for me there is no part that is challenging but there are other colleagues that work in other labs that are,that do not do the DAT testing aah'it's challenging because if you are not exposed to doing it each day.. each other day,in sometimes you might mess but all important is the part of parasitological diagnostic of kala Azar especially for relapse cases that is one part that as a county that we have not been trained as a medical lab oversight ,we have not been trained on parasitological diagnosis of kala Azar relapse cases.So that is the only deficiency currently and training on parasitological diagnosis of kala Azar relapse cases.

l)What part of VL diagnosis,care and treatment is most enjoyable to you?
RES: aarh' ..for me the most enjoyable part is after the patient have done with the treatment and did not present again with the same same signs and  symptoms they came with the first day to our facility,that is the part that is enjoyable to me and many of our health care workers to see that the patient that we ,that came in has recovered and have been cured of visceral Leismaniasis.And also to say that a  ,there is a constant supply of aah' the medication,the treatment for patients that they do not miss any ,they did not miss any medication.

m) Compared to Malaria how would you rate the VL burden in the county?
RES:... mmmh'..VL burden is high,and am saying is high because of the death that is normally associated with VL compared to malaria it's,there is readily available treatment of three to five days compared to Kala Azar that is ,that has a treatment of 28 days or 17 days.And the cure rate is not compared to malaria.So the burden that is normally associated with the death that come due to anaemia in Kala Azar makes me to say that the burden of kala Azar is higher compared to malaria because eeh' Kala Azar is not associated with short rains , it's always there.It is not like malaria that is normally ,that we see after,shortly after the short rains.So kala Azar is always there with our patients there level of exposure and localities.

n) Can you tell me on the relationship between HIV and Kala Azar?
RES: aarh'... basically there is no relationship because somebody who is HIV positive can either have kala Azar or not have kala Azar but the relationship is when you have, the chances of death normally increases when somebody has both VL in conjunction with kala I mean aah'HIV.Yeah.

QUE 2
a)How prepared do you feel to handle the provision of VL services within this facility?
RES:In this facility,I can say that we are prepared because we've enough human resource but if we on the flip side if we compare to other facilities that have insufficient or inadequate number of health care workers, becomes a challenge because we need more human resources to handle kala Azar patients together with other patients that we normally receive rather than that we normally receive but with LCRH as a case we have enough human resource to handle the issues of Kala Azar.

b) What are your concerns about work demands that may come with managing VL cases in your facility?
* How willing are you to perform VL screening as part of your work routine?
RES:For LCRH aah'..we do not have some funds because we,as I said earlier we have enough human resource that if mobilized well within by the management of the facility,we can be able to handle aah'..kala Azar patients.

*How willing are you to perform VL diagnosis as part of your work routine?
RES: aaah'. ..am willing ,am ready to perform because am trained,I don't have a reason to say am not ready to perform.

*How willing are you to perform VL treatment as part of your work routine?
*RES:I am not willing to do treatment because it's not within my area of expertise.My area of expertise is only the diagnostic area.There  other health care workers that are  trained and within their area of jurisdiction and are able to carry out treatment.

*How willing are you to perform VL stock management as part of your work routine?
RES:For my case aah'..am able to do stock management regards only to the testing kits.All the items that are concerned with diagnosis is one area that I am willing to do ,to manage well as I receive.

*How do you do data reporting on VL?
RES:yes ,am obligated to do reporting on VL commodities that are concerned with diagnosis every month.

c)Has managing VL cases in your facility In any way affected your work schedule or your well-being?
RES: No . Management of VL patient has not affected our way of work because like I said we have enough human resource that have been mobilized to take care of VL cases... aah'..the only challenge we face with regards aah' VL patients Is  aah' when we run of the Rk 39 commodities and now have to do DAT and not everyone within the facility has been trained on DAT a number of us have been trained and we have done, although we've done mentorship and OJTs , sometimes people would want to have a real training to be able to now to do the DAT.But when we have enough Rk 39,then we are not chal,we normally have no any  challenges except when we have faced with the fact that we have to do DAT.

d)Have you received any specific training or skill development related to provision of VL services?
RES:Yes,I have received training on a kala Azar as a whole including 'aah' including the DAT training.
The training was conducted by W.H.O between 2018 and 2019 and another was done on 2023 by FIND.
The training has helped me because am now able to carry out DAT testing when we do not have the Rk 39 or when we need to confirm when somebody is negative on Rk 39 but still has symptoms of kala Azar,now we are forced to confirm using DAT.

e)Have you received more resources e.g,personel/ equipment to help you manage VL cases following decentralization of VL care in the county?
RES:'Eeh' we have received some items especially the pipettes,we have the multi-channeled pipette,that we normally use when we are doing DAT testing.We have not received any personel.We normally rely on personel from the county government.There is no personel from FIND or any other partner.

f)Do you think that bringing visceral Leishmaniasis services to this clinic has in any way affected other services at the facility?
RES:No it has not affected other services in the facility.

QUE 3:What does the community say about VL and what is the impact of such perceptions on care seeking?
RES:"aah'..that VL is a terrible disease,a killer disease but also they appreciate the fact that aah' currently there are medication that there,VL is able to be diagnosed and treatment in their facilities.The only concern is a the period of treatment and the aspect of nutrition especially for those that are normally , especially for children that are malnourished.They ask if their is any provision either by the county government by the program to assist and to address the issue of nutrition when they are taking this medication because of the long period of medication.

QUE 4:If we were to roll out VL diagnosis,care and management programs to other health facilities,what areas would you recommend we improve?
RES: aah'if it's possible,if it's visible aah' the other facilities that do not have enough human resource to handle both VL patients, treatment and the normal other patients that normally come to the facility.So if they could address the issue of human resource, aah' even with some facilities there is aspect of infrastructure especially the labs .if we assist it will be good.Maybe the training ,the  training especially the DAT ,DAT training aah' and parasitological diagnosis training for kala Azar relapse cases are very very keyto the healthcare workers instead of going to Kacheliba or Uganda to seek for diagnostic services for relapse cases and also for the treatment.


QUE 5:Whom do you think should be trained at the community level to Improve health seeking behaviour for VL patients?
RES: I believe if we can train the community health promoters they will be better placed because of their localities to educate the community on the risk of exposures and actually on kala Azar as an entity especially to reduce the risk of exposure to the sandfly bites that are normally aah' more to the communities where young  children or even adults are herding their cattles and they do not know that ,where the vectors are.
Aah' even health care workers can be trained cos they can have ,cos they still interact with the patient and the community.They can still offer same same education.

*Do you have any other question?
RES: Mine is not a question actually,it's a concern as a county we need to do a training especially for medical lab officers on the parasitological diagnosis of kala Azar relapse cases.
