# Supplementary material for: Barriers and facilitators of care among visceral leishmaniasis patients following the implementation of a decentralized model in Turkana County, Kenya
Source: PLOS Glob Public Health. 2025 Mar 31;5(3):e0004161. doi: 10.1371/journal.pgph.0004161 (PMC11957299; doi:10.1371/journal.pgph.0004161)
Supplement: S1 Data — This file includes the following transcripts: •VL Patient In-depth Interview Transcripts: Verbatim transcripts of interviews conducted with VL patients, capturing their insights and lived experiences. •Healthcare Worker Key Informant Interview (KII) Transcripts: Transcripts from key informant interviews with healthcare workers, detailing their perspectives on decentralized care models for VL. (ZIP) [file pgph.0004161.s003.zip › HCW and IDI transcripts/patient interviews/Res 001_FACILITY 1.docx]

VL DECENTRALISED STUDY

VL PATIENT/CAREGIVER INDEPTH INTERVIEW

**INTERVIEW**
QUE 1:How many days have been admitted in this facility?
RES: I came here on date xxx this month.

Que: how many weeks now?

Res: Three Weeks now.
(background noise)

QUE 2:Tell me about the condition for which you are suffering from?
RES: I am suffering from Kala Azar

QUE 3:What do you think causes the disease you are suffering from?
RES: I don't know what causes it. I got surprised when they said I have in the hospital.

Que: what do people say brings this disease

Res:I Don't know maybe if I can ask other people.
(background noise)

QUE 4: Briefly describe some of the symptoms you experience?
RES:I was sick like that, sleeping alot and they brought me to the hospital..So they brought me to the hospital and when I came to the hospital,they tested me and said I don't have blood.When they say I lost blood,they said another disease you are sick is Kala Azar mmmmh,,,,.Just like that…..So they said that there is it's medicine.mmmmh

QUE 5:From where did you learn about the condition you are suffering from?
RES:I heard it from this hospital.

QUE 6:Is there any member of your household or community member you are aware of that has suffered a similar disease?
RES: No nobody was sick .

Que:Have you ever heard someone who was sick in the village?

Res.I hear sometimes of someone sick of Kala Azar there.I also heard of one villager who was sick.

Que:Did he got treated?

Res:yes he got treated.

Que: Did he get healed?
Res: He got healed...mmmh..

QUE 7:Do you think think condition is a big problem within the village you come from?
RES:Yes a big problem...it's a problem in the place I come from…….It affects people.

QUE 8: Compared to malaria and other conditions, how would you describe Kala Azar burden in your area?
RES:..burden.wooi....this disease is very heavy...
(background noise)

QUE 9:Whom do you think is most at risk of getting Kalazar?
RES: Anyone gets Kala Azar...

Que: which areas are most prone to Kalazar?(phone conversation in background)

Res: in Loima..I don't know .. It's when you have studied it you have the knowledge.

Que: what are the factors that contribute to kalazar infection?
Res: It is the person who has studied about this disease is the who knows..will find it comes in this way and that.

QUE 10:Tell us more about the disease and how you think it is spread?
RES: I Don't know how it's spread...how should I know..

Background noises)

QUE 11:What do you think you can do to protect yourself and your family from the disease?
RES:I don't know as I said mmmh ..the government should know where it comes from and stop it."eeeh...

QUE 12: Briefly tell me how the disease is diagnosed?
RES:It's the blood..mmmh.

QUE 13: Briefly tell me how the disease is treated.
RES:They give me injections

Que:How many injections are they?

Res:they said Nine...  17 injections. "(Other patients conversing)"...

QUE 14:When did you first become aware that you were ill?
RES: ... waaai....when something takes you down ,you become weak.When you wake up in the morning and find the body sick.

QUE 15:What are some of the symptoms you experienced?
RES:...I don't even know.

Que:What did you experience in your body?

Res:what did i saw..I just saw that am sick and came to hospital as usual... nothing else have seen coming.

QUE 16:What symptom made you feel the most need to visit the health facility?
Res:I just saw sickness.. nothing else.

QUE 17: For how long did you have the symptoms before visiting the facility?
RES: four months.

QUE 18:What made you wait for those 120 days before seeking treatment?
RES: hospital money...and they are still owing me.

QUE 19:Did you seek any alternative source of treatment before coming to the facility?
RES:No..no I was just there at home.

QUE 20:What are the challenges you experience as a Kala Azar patient?
RES: in terms of hunger ..in other ways too.

Que:Any other challenge?

RES:Scarcity of food to eat..to reduce and subside. To give strength when taking the drugs.

QUE 21:What factors motivated you to seek help outside your household for your illness?                     
RES: I came on foot to the hospital

Que: Where did you come from?

Res:I came from Loima.

Que:Why did you choose this hospital?
Res:I inquired of the treatment here.

QUE 22:What measures helped you during the process of care seeking?
RES:.. nothing else helped me,I just walk here like that...there is nothing completely.

Que:What helped you here?

Res:what helped me is just the drugs I take...there is nothing that helped.

QUE 23:Among your household who decides on whether to seek or not seek care when a person gets sick?
RES:nobody decides,when they see someone is sick,and you know in the hospital is where you find help ,they take you.

Que:Who decides whether to go to the hospital or remain?

Res:why should you remain..to do what at home?.
Everybody can say you go to the hospital.Neighbours too,any family member.

Que:Who exactly in your household?

Res:(chuckles)"write  anything if it's me or my father.

QUE 24:Were you aware you could get diagnosis and treatment for kala Azar in this facility before you fell ill?
RES: No, I never knew ,I just came to the hospital to drink medicines when I became sick..eeeh' the hospital...the disease..has it's drugs.
I just came like that to the hospital,so they diagnosed of kala Azar.

QUE 25:Where do community members seek help for Kalaazar?
RES:There is nowhere they look for... They just try their luck.They treat their usual disease.They treat with traditional trees.
They talk that it's a bad disease..their is nothing else ..

QUE 26: Please tell me of your experience on the health care you are receiving?
RES:...you asked about that just now..I got help..help aaah'... help.

Que:For how long did you wait for the results and started treatment?
Res:   ''eeh...." from early morning to evening ..I have even got anything..I didn't even eat that day upto evening.

Que:How are you getting treatment?

 Res: ..drugs..there is the one that is injected and their is the one that given through water.They give through a line on the hand.

Que:What are your concerns on the care you are receiving?
 Res:  ""Eeh it's okay.

Que:Are there any oral?

Res: There is no oral..but there is a oral one ,

Que:How are the orals?

Res:it's not sour

Que: How's their side effects?

Res:.When you drink you become weak and sleepy.It doesn't want you walking.

Que:Do you think the medication is going to heal you?
 Res: I don't know if it's going to help me..either it will..but upto when you will see you are healed.

Que:What are your concerns on follow up?
 Res: I want like to be followed at home to test me.

QUE 27:What kind of support are you receiving from family and friends to help you cope with the long hospital stay and Kala Azar treatment?
RES: Nothing..there is nothing am getting.No one has ever come ..'

que:It's just you alone?

Res:...mmmh' when you feel like buying oranges but you are just alone.
No...which money again..I don't have anything.When I see you people I say you have come to help.

QUE 28:How much does it cost you as a Kala Azar patient,in terms of personal expenses?
RES:There is nothing I have given out.

Que:Is the treatment for free?

Res:They write them on the book.No."mmmh" it's payment.They are writing the money down and when I am discharged

Que:How much have you paid so far?

Res:have not paid anything.

QUE 29:In considering the steps you took what do you think you would do differently now if you could start from the beginning?
RES:'like what' problems or...I will go back home ...I would just go back home and stay..or does it this disease stay at home that you will get.

QUE 30:What changes or interventions would you suggest to improve Kala Azar care and access to Kala Azar care?
RES:"do how'..I don't know that...",(conversing with another patient "...)

QUE 31:If any of your friends or relatives developed Kala Azar, What would you recommend to them in terms of treatment?
RES:I will tell him to go to the hospital.

QUE 32:Are you aware of any past interventions for Kalazar in the county?
RES: like I told you earlier,i heard from the people and also one of the villagers was sick.

QUE 33: Kindly give more information about the barriers to access of VL diagnosis care and treatment?
RES:The government makes it hard.To follow up on people

Que:Any other addition?

Res:That is the only ones I Know.

Que:How does the government makes it hard?

Res:It makes a bit hard In  terms of bringing money and also helping people.And testing this disease that it is in Turkana so that it spreads the information that is in a place called this and that and also this...and bring that drugs. If not now..

QUE 34: Please tell me what type of people have the greatest challenge accessing VL treatment and why?
RES:The Turkana will not get .

Que:In terms of people who gets it hard to access VL services?

Res:The Turkana who else?..

Que:Why the Turkana?
Res:Because of illiteracy.They don't know where the drugs are.
(""Inpatient noises")

QUE 35:What are the measures you feel should be put in place to address the barriers and improve access to VL services?
RES:They should build hospitals..they should build hospitals, bring that drugs if you want to bring.

QUE 36:What can you tell me about the risk of developing VL once a person leaves Turkana county and if you are aware of any available resources outside Turkana county?
RES:You can find also treatment from outside.''eeh ..like now Uganda.Ye, they say there is it's hospital there that is helping other people from Loima."  Yawns")

QUE 37:What do community say about the condition you are suffering from?
RES: They say it's a bad disease.

QUE 38:What is the impact of community perceptions on VL care and diagnosis?
RES:They don't spoil.When they hear there is a treatment somewhere that's where everyone goes.They don't talk to you tha,t this and that.

QUE 39:What can be done at the community level to reduce stigma?
RES: There's no way have seen them say is bad.They say that because it's a disease that does not get to you easily.Its a disease..it's a disease that is not easy to find.Like TB and another one called...

QUE 40:What is the best way to involve the community in strategies to combat and control VL?
RES: they should bring..they should find how they should plan that you should get .

Que:Plan in which way?

Res:In terms of hospital,they should bring doctors there.

Any other question?

Res: I don't have a question but I would like the government to build the hospitals and tell people that there is a certain hospital this and that,for this disease so that all people will go there.The sickness can come anytime either at night even if you train at daytime,when you train let's say at daytime you will not know if you will be sick at night or he/she will wake up okay.

*Tell me another barrier to accessing VL diagnosis and care.

Res:Money is the biggest barrier to accessing this treatment.And because this disease is costly the Turkana people will not be able to afford it.We want this treatment to be free of charge.As Turkana we say bring many drugs because many Turkana people are dead and others are continuing loosing their lives. That's why I say that.
