# Supplementary material for: Barriers and facilitators of care among visceral leishmaniasis patients following the implementation of a decentralized model in Turkana County, Kenya
Source: PLOS Glob Public Health. 2025 Mar 31;5(3):e0004161. doi: 10.1371/journal.pgph.0004161 (PMC11957299; doi:10.1371/journal.pgph.0004161)
Supplement: S1 Data — This file includes the following transcripts: •VL Patient In-depth Interview Transcripts: Verbatim transcripts of interviews conducted with VL patients, capturing their insights and lived experiences. •Healthcare Worker Key Informant Interview (KII) Transcripts: Transcripts from key informant interviews with healthcare workers, detailing their perspectives on decentralized care models for VL. (ZIP) [file pgph.0004161.s003.zip › HCW and IDI transcripts/patient interviews/Res 0012_FACILITY 4.docx]

VL DECENTRALISED STUDY

VL PATIENT/CAREGIVER INDEPTH INTERVIEW

PATIENT INTERVIEW

QUE 1: How many days has your child been admitted in the hospital?
RES: It is now 4days in the hospital.

QUE 2: What kind of condition is your child sick?
RES: Kalaazar

QUE 3: What causes Kalaazar?
RES: Maybe it's dirty water, you know in reserve areas there is no clean water .People consume dirty water.I think that was the causes it.

QUE 4: What are the symptoms you saw in your child’s body?
RES: Fever at night ,,mostly at 7pm his body weakens and get cool, even now his veins are not visible and yesterday he didn't receive his injection as the doctors looked for his veins but no vein seen.

QUE: What else did you see on his body?

RES: He is also loosing weight and his body going to be destroyed.His belly also protruded and it seems his blood is becoming low.Yellow eyes and cracked lips and that in Turkana we say maybe that's what this disease does to the body.He his not feeling well.

QUE5:Where did you get the information that your son is sick of Kalaazar?
RES:(("birds chirping in the background") The doctors are the ones who told me after being tested.

QUE6:Have you ever heard of Kalaazar disease before?And if yes was he/she treated?
RES: My sister was sick of kalaazar on Jan and feb.She had a protruding abdomen.They tried treating her the cultural way,cutting her body saying that it could be Turkana cultural disease .Every afternoon she becomes more serious with alot of fever all over her body but she didn't get healed.But later she was taken to the hospital and given medication.She got healed of kalazaar.

QUE: Is there anything you want to add?

RES: When she takes a little food even water the stomach protrudes .She vomits even water.

QUE 7:Do you think this disease is a challenge in the community?
RES:This disease is a big challenge for us living in rural areas because we have knowhere to get help.Upto when you come to the hospital is where you find help.When you come to the hospital is when you get tested what you hail from and they help but there in the community they say many things,,"ooh slaughter a goat for him ,get some herbal medicines.What I have known is that this disease cannot be treated in other way but come to the hospital ,,get tested and be given medicines.

QUE8: What's your comparison of Malaria and Kalaazar on burden of each?
RES: It's this disease that's heavy"mmmh""(birds chirping in the background)

QUE9:Who do you think is at most at risk of contacting Kalaazar and why?
RES: I can see everyone can get this disease.I can't say there is a specific person who can get Kalaazar, because it's a disease sent by the devil"aah "or it's for anyone.

QUE: Which places here are prone to this disease?

RES: Maybe this side of Sudan is where it's prone.

QUE: Where else is prone to the disease?

RES: Many people in Turkana also get Kalaazar and it's always mistaken for the liver disease and that's why they make some cuts in patients body when you have the symptoms.

QUE; Which category of individual does it affect?
RES: The most affected are the children.

QUE: At which period of time does it affect mostly?

RES:I don't know the environmental factors that are associated by Kalaazar but I think it's during the rainy season."mmh" where there are stagnant water or brown dirty water in the rivers.

QUE10:How is Kalaazar spread?
RES: (phone ringing and people chatting) I don't how it's spread because I don't think if it's a disease you can contract from the tree .This disease comes in form of malaria, the abdomen becomes big (someone on phone)
It's just like that when this disease strikes it makes a person not able to eat the available meals like maize .You can seen someone's belly become big and it's also judged as maybe a stomach upset .The skin becomes scaly and when you bring a person to the hospital,he is tested and they know what you are suffering from. The disease is most at Turkana.

QUE 11: What do you think you can do to protect yourself and child from getting Kalaazar?
RES: I don't know how to protect myself from getting Kalaazar (hospital trollies being moved)

QUE: “mmmmm”

RES: You need to do is to drink your medicines and the other that has not contacted Kalaazar you will not be able to know what to do to protect you from getting it.

QUE: 12 :How Is this disease diagnosed?
RES:"In the hospital or at rural areas?

QUE:In hospital

RES: They take blood  sample for diagnosis.They test for Kalaazar if it's there or not.

QUE 13: Briefly tell me how the disease is treated?
RES:They insert a line in the hand and administer one drug,and one injections on the thigh.

QUE 14:When did you first become aware that your child is ill?
RES:He was sick not so long .

QUE: For how many week approximately?

RES: But being diagnosed of Kalazaar it's now one week."two weeks ...no a week now with him in the hospital.

QUE15: What symptoms did you experience in your child body before coming to the hospital?
RES:"paper flown" hotness of the body, protruding abdomen,I saw and knew it's not a familiar disease and that's why I brought him to the hospital.its the hotness of blood and big belly.

QUE: What else did you see

RES:.How his body it is now and the his skin became scally like burnt skin.""mmh"

QUE16:What symptom made you feel the most need to take your child to the health facility?
RES:It's protruding abdomen that he exhibited.

QUE17:For how long did your child experience the symptoms before coming to the hospital?
RES:it's three months before coming to the hospital.

QUE18:What made you wait for 90 days before seeking treatment for your child?
RES:He was in rural area .There they thought it's a small issue .They were taking him to dispensaries in the rural part. He was always given malaria tablets. He was far from me.

QUE19:Did you seek any alternative source of treatment before coming to this hospital? How did you manage to control the symptoms?
RES: He started from komen health center but bought medicines from chemists . They said it was malaria. No one tested blood to know if it's Kalaazar. They took blood sample for malaria only.

QUE: Was he treated malaria successfully?

RES: He was treated malaria but what remained was a protruding abdomen.

QUE20:,,,,,,,,,,,,,,,”mmmm”What challenges do you experience as a mother to a Kalaazar child?
RES:Stress on what good food he will eat, I don't sleep at night and crying all the time.Also thinking on what to give him to eat that will bring back his blood.

QUE; Is there any other challenges?

RES: The other challenges maybe when He is treated and not recovering.,,,,,,,,,,,,,,”mmm”

QUE 21:What made you seek treatment outside your household? What made you choose this facility?
RES: Because I saw the previous hospital didn't have the power to diagnose and treat the disease inside my son's body.That is why I chose to bring my son to be tested to know what he is suffering from.He got diagnosed here of Kalaazar.,,,,,,,,,,,”mmmmmmm”

QUE 22:What measures helped you during care seeking for your child?
RES:His blood became low and they were told to pay for money and also look for some people to donate blood for my son's blood transfusion.So I started begging for help so that my son could get blood.Thats why I came here.The low blood count and how I saw him almost dying.I decided to bring him to a big hospital so that he could get help

QUE: What helped you while seeking treatment for your child?

RES:.There was nothing else I did,only medication helped me.

QUE 23:Among your household who decides on whether to seek care or not when a person gets sick?
RES:It's supposed that if someone becomes sick He is supposed to be taken to the hospital to get help.""birds chirping"

QUE24:Were you aware you could get diagnosis and treatment for Kala Azar in this facility before?
RES:I never knew I just came unknowingly.

QUE: Did you heard from someone?

RES: I heard that it's medication is here.I heard from someone who was sick in our family that where I knew there is medication.

QUE25: ,,,,,,,,,,,,,,,,,,,,Where do your community members seek help for Kalaazar?
RES:They come to the hospital.

QUE: What do they say about the Hospital?

RES: They talk of the goodness of the doctors and the hospital……”mmmm”

QUE26: Please tell me on your experience on the treatment and care you are receiving.
RES:I slept in the hospital for 3days when my son was still under blood transfusion……”mmmmm” and the next day he got discharged…….”mmmm” the line was put on his hands.They took blood sample that day.
They administration of medicines is okay, there are no oral drugs given and I believe he will get healed.
"Like what now""mmmh" I can say if the government can help in support.Help like that is what I need .If my son gets healed it's good right?If the government is good also it can provide with aid to help them.

QUE27: What kind of support are you getting from friends and family that makes you cope with the long hospital treatment?
RES:There is no support.

QUE28:How much does it cost as a parent of a kala Azar patient interns of personal expenses?
RES:I have used alot of money

QUE: How much did you spent?

RES: I have sold three goats because there was no cash.It is almost ksh 8000.I used to pay for blood transfusion, testing for kala azar and for transportation through motorcycles.Its just that and some was used to buy some small things for him to eat.I didn't not pay for doctors fee and also foods.All in that amount……”Birds charting”

QUE29:In considering,the steps you took,what do you think you would do differently now if you could start from the beginning?
RES: "If I would start from the beginning''...'in care seeking or' they used to treat culturally those were the ways they were doing back home.They gave him animal blood to drink also his body was cut,just turkana things.There were no other ways I did to him,I just used herbal medicine to treat him.'mmmh'what I would have done is bringing him to the hospital early he could have been well now.

QUE30:What changes /interventions would you suggest to improve Kala Azar care and access to Kala Azar?On diagnosis and also care and treatment.
RES: Diagnose the blood so that to help in medication.Any aid from the government will help them.
I want good medicine to be found so he would be healed.

QUE 31:If any of your friends or relatives develop Kala Azar ,what would you recommend to them in terms of treatment?
RES:I would tell them to go to the hospital so that they get treatment.

QUE32:Are you aware of any past interventions for VL in the county?
RES:I will not know because I am an illiterate person..'mmh'

QUE33:Kindly give me more information on the barriers to access of VL diagnosis care and treatment?
RES: People are getting a lot of problems.

QUE: What kind of problems?

RES: Some people find them having nothing, taking drugs like that with no food

QUE: Why is it treatment also a challenge?.

RES: It's a challenge. People are suffering alot.
'hee'no 'That is right' when they diagnose someone gets medication.
The long distance is bringing this disease and there is anthills near our homes.
Lack of money is also a challenge for testing blood so that to be given drugs .

QUE 34: Please tell me what type of people have the greatest challenge accessing VL treatment and why.
RES:The illiterate people.

QUE: Which category of individual mostly?

RES: Children 5 years and the elderly of 50-90 years.

QUE: Why them?

RES: Children because of lack of money and the adult because they don't have strength.

QUE35:What are the measures you feel should be put in place to address this challenges and improve access to VL services
RES:They should help with nets….”mmmm” and also monetary…”mmmm” aid should be brought.The government and companies should come near the illiterate people too so that they give us medication near.

QUE: What else?

RES: We need doctors to our near facilities to give us drugs near instead of getting problems travelling to far places like here and that's money too……..”mmmm”

QUE36:What can you tell me on the risk of developing VL once a person leaves Turkana county?
RES:Someone can contact the disease from mosquitoes and there are those flies in bushy places that's how a person can get this disease.

QUE37:What do community members say about kala Azar and those sick of Kala Azar?
RES:They are saying in the badness of the disease.

QUE: How does it bad?

RES: It is bad if you haven't gotten treatment.Its also deadly.

QUE38:What is the impact of community perceptions on VL care and diagnosis?
RES:They encourage you go to the hospital and get treated.'birds chirping'they help by telling someone to look for medication.

QUE39:What can be done at the community level to reduce stigma ?
RES:The government should bring aid like nets distribution .That's what I see it will help people instead of being sidelined.And other thing is financial aid to people,,,,,,,,,,,,,,,,”mmmm”

QUE40:What is the best way to involve the community in strategies to combat and control VL

 RES:The doctors and the government should come near people,so that there will be Great help to all people.Other support by the government.

QUE: What else do you think they can do?
RES:Training should also be conducted.And financial aid too should be given by the government.

We can have to the end of the interview, do you have any question for?

RES: Have not seen any meeting like this since I came here, How does this come and how does it help us?

QUE: I started to inform you that we come here because we want to bring this Kalazar treatment to the near health centre, we were here to see how we can improve and provide to the facilities with no VL services in terms of diagnosis, treatment and management in Turkana.

RES: Okay.
