# Supplementary material for: Barriers and facilitators of care among visceral leishmaniasis patients following the implementation of a decentralized model in Turkana County, Kenya
Source: PLOS Glob Public Health. 2025 Mar 31;5(3):e0004161. doi: 10.1371/journal.pgph.0004161 (PMC11957299; doi:10.1371/journal.pgph.0004161)
Supplement: S1 Data — This file includes the following transcripts: •VL Patient In-depth Interview Transcripts: Verbatim transcripts of interviews conducted with VL patients, capturing their insights and lived experiences. •Healthcare Worker Key Informant Interview (KII) Transcripts: Transcripts from key informant interviews with healthcare workers, detailing their perspectives on decentralized care models for VL. (ZIP) [file pgph.0004161.s003.zip › HCW and IDI transcripts/patient interviews/Res 002_FACILITY 1.docx]

VL DECENTRALISED STUDY

VL PATIENT/CAREGIVER INDEPTH INTERVIEW

**Interview**

Que:,,,,, Want to ask you?

Res: "Mmmmh"

Que: Which Day and date did you brought your child to this facility?

Res: I brought him on Wednesday

Que:"mmmmh"

Res: Of past 3weeks now.

Que: On Wednesday?

Res: "Yah" the 1st Wednesday, last week then this week.

Que:"mmmh"

Res:"mmmmh" on Wednesday, Wednesday I come (coughs) here early Morning (coughs) then evening I get into the ward.

Que:,,,,, So you have taken 3 weeks and half?

Res: "eeeh"

Que: It is good. Want to ask you the 2nd question "(coughs)" what did the doctor say your child is suffering from? Here at the Hospital.

Res: He said " He is suffering from "etid" also known as Kalazar.

Que: He is suffering from "Etid?"

Res: "eeeh" it's called Kalazar in English.

Que: "eeeh" it's called Kalazar.

Res: we said it in Turkana "Etid"

Que:"mmmmh "

Res:"mmmmh "

Que:,,,,,,,,,,, as you see?

Res: "mmmmh "

Que:What did you see (coughs) on this child body and said He is suffering from Kalazar?

Res: Stomach get full

Ques: Mmh

Res: The Stomach get full...

Que:"mmmmh"

Res:,,, The Stomach get full, when the stomach get full..?

Que:"mmmmh "

Res: It is seen. And when you touch the person,

Que:"mmmh"

Res: you could identify this is Kalazar

Que:"mmmh"

Res: "mmmh"

Que:,,, A part from his stomach getting full what else did you see?

Res: When it started..?

Que: "mmmh"

Res: At first, it started as a Malaria.

Que:"mmmmh "

Res: Malaria,Malaria as you take to Hospital, yet its sucking inside. The tablets he is taking?.

Que: mmmh"

Res: It's not of that disease.

Que:mmmh"

Res: so it can't be effective with those tablet rather than it worsen and can be identify that this is Kalazar and that's not the end is Malaria when it reaches at evening...

Que: mmmh"

Res: It makes your body hot, this other time you're good, you play good and when it reaches at the evening makes your body hot or makes your body hot at Morning.

Que:mmmmh "

Res: eeeh"

Que:"ooh"

Res:"mmmh" (yawns)

Que:,,,,As you can see

Res:mmmmh "

Que: what could you think that..

Res:mmmh"

Que: Bring this kind of disease?

Res: I think what have seen..

Que:"mmmh"

Res: It is brought by dirty water that we usually drink from the River that brings this disease.,,,,,,,,, Dirty water that we share with everything………….. pokots drinks, Us and also the river water is dirty,,……….

Que: oooh"

Res: mmmh"

Que:,,,,, and where did you know that, people are suffering from this disease. As anybody tells you about this disease called Kalazar.

Res: I learn from myself, was suffering from the disease.

Que: you are also affected by Kalazar?

Res: I was suffering from that disease but no body takes me to Hospital. I was hearing daily people going to Kachiliba seeking for treatment and get back home successfully. In fact there was a boy who goes there, right now he is health guy.

Que: Where is Kachiliba?

Res: Kachiliba is located at West Pokot.

Que: Is there treatment..?

Res: aah?

Que: Is there good treatment given to people there?

Res: Kalazar medicine that is there.

Que: mmmmh"

Res:mmmmh"

Que: Is it herbal medicine, or this other modern medicine?

Res: It is a medicine like the one we are taking here.

Que: That of Hospital?

Res: Hospital one.

Que: Are they going to Hospital or they meet an individual there?

Res: Me have not gone there, but the people they go there

Que: mmmh"

Res: They went with illness and come back well.

Que:"mmmh"

Res: It good.

Que: yah, actually it's good (coughs). And when you're suffering from kalazar what did you see on your body? Are those symptoms same with that of your child or yours are different?

Res: I Was sick when I was still young like this boy

Que:mmmmh "

Res: So nothing was know more about it.

Que: So nothing you can say about it?

Res: nothing I can I say.

Que: And what when you have been told about Kalazar by other people.

Res: They told me that

Que:mmmh"

Res: I was suffering from the disease while at the remote area, where there is no Hospitals like this one

Que: mmmh "

Res: I treat there and the person I was with, fail to understand that am suffering from Kalazar

Que: Mmmm"

Res: She treated like that till when my Dad came. He was at the big homestead, He came and carries me to Herbalist who Identify that this Kalazar.

Que:mmmmh"

Res: That is how people narrated about my illness.

Que: Do those people say there was No dirty water those days?

Res: Where we live?

Que: Yes?

Res: We were drinking this dirty water from the Wells

Que: Those of Wells?

Res: Mmmh"

Que: So then, you see that dirty water causes it?

Res: "yeah" I think it is dirty water and also we take meat.

Que: mmmh"

Res: Does it also transmit through meat?

Que: Not sure, maybe all those dirty stuffs your have been taking may cause the disease.

Res: Mmmh"

Que:,,,,, so it's dirty water, fresh milk.

Res: Milk,meat and those dirty water from Wells that people usually bath on it and use it for drinking at the same time.

Que: "oh",,,,, and those days you are sick, did sick treatment at West Pokot or only

other people...?

Res: Other people, I never went there.

Que: Other people you're listening that they seek treatment there?

Res: "eeh" that when they go there, they were injected and get back successfully.

Que: Does He/she goes for an injection for a day and get back or what?

Res: They go and take Days that are subjected to the treatment. If it is 22 days?

Que: mmmh"

Res: Or 30 days

Que: mmmh"

Res: If your disease seen its chronic you will be subjected for 30days and if it is light you will given 22 days, and if it's at early stages you will take 17 days

Que:"eeeh"

Res: Depending on stage on which your stage reached.

Que: It is right.

Res: yes

Que: And does day come out successfully with no other infections?

Res: yes they come out healthy, with no any disease.

Que:So the disease didn't get back to them after some days?

Res: They are just good, some are married

Que: "mmmm"

Res: Unless they are affected by other diseases

Que: "oh" it is okay

Res: "mmmmh" you can't see that disease again.

Que: As you can see this disease, does it affect everybody at your village? Or some of the people.

Res: They were many affected with the disease.

Que: So it affect many people?

Res: It affects it is only me that come this side, other people seek treatment at Kachiliba.

Que: Mmmh"

Res: Mmmh"

Que: They went that side of Pokot to seek for treatment?

Res: They went to seek medication that Pokot side "(phone rings)"

Que:"wuueh!!"

Res: mmmh"

Que: As you compared this disease with Malaria which one is a burden?

Res: Kalazar is a bad disease, Malaria is better because you can take some Panadols and feel better, this one is worst.

Que:"mmmmh " ,,,,,,,"(Child cries)" As you can see at that village of yours that you said people are suffering, which category of individual is most of getting Kalazar?

Res: Children's

Que: Small Children?

Res: Those Small children.

Que: What made them get affected easily?....what is making them to be affected???..

Res:That's what I don't know

Que:mmmmh "

Res: Is it water made them sick, this dirty things that are everywhere that brings this disease. All those things are there

Que: They are there.

Res: So nothing can makes you identify the cause"(child talking in the background)"

Que: When do people or children get ill in large number?

Res: That disease starts on August 2022 during election period

Que: mmmmh "

Res: At this place of ours, it started on August last year.

Que: Was there any Rain?

Res: It was raining.

Que: The place was prone of raining?

Res: It is raining even right now

Que: oh" So you said how the disease transmitted?

Res: The people live at different places, Some live a distance like that of Kanamkemer from the Referral Hospital. The disease started at the people living that far

Que: mmmmh "

Res: When the drought come, the people come together and that's when the disease spread.

Que: So it is like those other people from outside the village that brings the disease?

Res: They are the one brought the disease, and transmitted to other ordinary people.

Que: Are those people far from the village?

Res: They were far, and they were secured at one….at one area.

Que: Mmmh"

Res: They were far away, we listened them at far... Someone was sick from Kalazar,

someone is sick from Kalazar and when they get into our village that when most of people from the village suffered from the disease.

Que:,,,,,Where do this people come from?

Res: They came from Lokwar

Que: Lokwar?

Res: mmmmh"nakuse…

Que: When they came from Lokwar a place that's prone of Kalazar. What did you do to prevent from people spreading the disease.

Res: They were sick one by one. Somebody's son is suffering from Kalazar, So then when they came to our village. It is drought that forces them to come here so then it difficult to isolate them. All of us we are surrounded by drought, we end up being together, we die all here. So then when they came in, many cases of Kalazar disease identified.

Que: The disease get worst?

Res: The children get sick, not this not that one not the other one. And it started as Malaria, it can't start as a Kalazar.

Que: When you identify that the disease is on your village?

Res:mmmmh "

Que: What did you do to prevent the disease from spreading?

Res: Nothing you can to prevent like this migration that found us here, what will you prevent?

Que: To prevent is even how you cover yourself well..

Res: Maybe I improved my house structure and fence and secure your my people there "laughter "to prevent them from walking any howly and collecting dirt on compound.

Que: you secure your people on compound?

Res: yes, what else I could do?

Que: So you mean you will restrict them from walking?

Res: yes, the like of children you restrict them from playing near dirty water.

Que:oh"

Res: Mmmh "

Que: And when you bring child here for treatment or also how you had from the people, how the disease is treated?

Res: They take Blood transfusion, and taken to Laboratory for screening. And the Laboratory Identify you child is suffering this disease.

Que: So it's blood that is being taken?

Res: yes, It is blood being transfused

Que: .mmmh" and what kind of medicine did your son given?

Res: There are two type of the Medicine.

Que: mmmmh "

Res: There's is a maroon bottle and other small bottle

Que: 2 Medicine?

Res: only 2, one injected at buttocks and the other one at the arm.

Que: It is right.

Res:mmmmh "

Que: When injected there is no other tablets?

Res: No tablets.

Que: ,,,,,,,When did you first become aware that your son is ill?

Res: On August that is started.

Que: mmmh"

Res: It started as Malaria, when taken to Hospital, it get a month feeling well. Then it started again.

Que: When treated, get well then after someday it get back?

Res: It lack the right medicine for disease thus frequent illness.

Que:mmmmh "

Res: If it could be a thing that get the right medication I think it could have been treated well long.

Que: ,,,,What are some of the symptoms you experienced before coming to the facility?

Res: You see the body of the person, not Normal as usual " (Child talking in background), Fever, When it get evening the body of the child becomes warm, When it reaches morning, the son get well as usual. When it get evening every the body get warm...and we believe that Kalazar that made the body get warmer every evening.

Que: That is what they believe at your place?

Res: Mmmh"

Que: Kalazar usually comes at evening?

Res: mmmh"

Que: It is only fever, is there any other symptoms?

Res: No Other

Que: What symptoms made you feel the most need to visit the health facility?

Res: Lack of blood on his body and pale eyes

Que: For how long did you have the symptoms before visiting the facility?

Res: One month

Que: What made you wait before seeking for treatment?

Res: I was thinking its a Malaria, was taking to nearby dispensary Katilu and Lokichar dispensary..

Que: Did you seek alternative source of treatment before coming to the facility?

Res:I didn't seek.

Que: You never used herbal medicine?

Res: Have never used it before.

Que: You didn't cut the stomach like other Turkana traditions.

Res: I cutted the stomach "(Child talking in background) "

Que:What are the challenges you experienced as Kalazar patient?

Res:No challenges only that, the activities he was doing home get lost.

Que:What factors motivated you to seek help outside of your household for illness?

Res: I tried other facility but no changes and I was advice by in-law to seek further medication for a Child at this Hospital.

Que: Among your household, who decides on whether to seek or not to seek care when persons gets sick?

Res:At our Home we were 3.

Que: 3

Res: The first born died and the other went down counties. So I'm the only guy remain home

Que: you alone?

Res: Me, Dad and Father.

Que: mmmmh"

Res: So whichever it's happening, He is the one to decide.

Que: What about on the community who decides?

Res: A man is the one to decide the plans of his homestead……….

Que:Were you aware you could get diagnosis and treatment for Kalazar in this facility before you fell ill?

Res: I was aware.

Que: Where did you get the information?

Res: By people who treated there from Kalazar,…… also I was informed by in-laws that treatment is there.

Que: Where do your community members seek help for the condition your son is suffering from?

Res: They seek medication at Kachiliba in West Pokot.

Que: What made them seek medication there

Res: Because they got free medication there.

Que: Please tell me How your experience on the health care you are receiving.

Res:Since I came…… I started at the gate and pick card, I proceed to Laboratory and they transfused the child blood, after that they informed me that the child is suffering from Kalazar.

Que:"mmmmh "

Res: And there a lot of Malaria…pneumonia on his body, He started taking Malaria treatment. The Doctor also said that The blood level is low…they look for blood but they didn’t find until I donate blood for my child……..

Que: What kind of support are you receiving family and friend?

Res:No support….

Que:you only???...

Res:its only me…

Que: How Much does it cost you in your child treatment. In terms of personal expenses.

Res: Have used Kshs 4,000.

Que: only Four thousand…for treatment and medication…

Res: I used Kshs 4,000 for medication. And Kshs 3,000 for transport…..

Que: That four thousand is only for medication or what?

Res: It includes Medication charges and food. "(Phone rings)"

Que: In considering, the steps you took. What do you think you would do differently now if you could start from the beginning?

Res: If the child seek right medication early am sure He could be recovering much better now.

Que: What intervention would suggest to improve VL care and access to VL care?"(phone ringing) "

Res:It's good hygiene and Water

Que: Water to do what?

Res: Helping people with clean borehole water.

Que: What else did you see we have to improve?

Res: Mainly water.

Que: why water?

Res: Because we are using dirty water that everything uses, Some throw dead people there….and some people says the disease is transmitted through a sandflies from anthill.

Que: Which kind of sandflies?

Res: The green one I don’t know how it's being called.

Que: It brings diseases just like that?

Res:It comes and sting on your body and that's how it transmitted.

Que:That's how some people believes Kalazar come from, but yours you're sure it transmitted through water?

Res: Mine is dirty water that we drink causes it…so you cannot I dentify such water..if they are good or not…because all sort of things may be found in those water…

Que: If any of your friend or relative develop VL. What would you recommend to them in terms of treatment?

Res: I will advise him to seek medication at this facility

Que: mmmmh "

Res: If you go there you will find the right medication and you will get better….(background noise).

Que: Kindly give more information about the barriers to access VL diagnosis, care and treatment.

Res:Mostly transport

Que: What else? Is it only transport.

Res: it is only transport…so it is the only thing if you can’nt have it then…for example if want to go to Nairobi you must have transport to board a flight..so if you don’t have it you cant go anywhere….

Que: Tell me what kind of people have the greatest challenge of accessing VL treatment?

Res: It a challenge to all for even an adult.

Que:mmmh "

Res: When Old age person get sick frequently. It's high risk for him to get the disease…even the small children…so that’s where it emerge…

Que: What measures should be put in place to address the barriers and improve access to VL services

Res: The Government should provide Kalazar treatment to nearby facilities for emergencies because it can affect someone but no nearby treatment so he/she will lack away to that treatment so my suggest to bring those services to people and also a full access laboratory facility…so for my way I seek the medication like katilu facility there were no proper diagnostic tool for treatment of kalazar…

Que: What can you tell me about the risk of developing VL once a person leaves Turkana County and if you are aware of any available resources outside Turkana for VL care

Res: Not aware..but for in our side here Turkana there is that disease a lot that you can find many people are affected…mostly in our side of loima,,..cause I see it’s the water that causes such disease….

Que: What do community members say about the condition you are suffering from?

Res: they see the disease is dangerous it even kills if not treated..so even one person died after long stay without getting any treatment..

Que: What is the impact of community perceptions on VL care and diagnosis?

Res: they see as normal disease because they are now have knowledge that it can be healed….

Que: What can be done at the community level to reduce stigma?

Res: so to tell the community that this disease is just the way person can get so no way to separate people and also you as person can get the disease…

Que: What is the best way to involve the community in strategies to combat and control VL

Res: by organizing cinemas…. Awareness and educating the community on ways and management of the disease..

Time: 36min and 8 sec interview.
