# Supplementary material for: Barriers and facilitators of care among visceral leishmaniasis patients following the implementation of a decentralized model in Turkana County, Kenya
Source: PLOS Glob Public Health. 2025 Mar 31;5(3):e0004161. doi: 10.1371/journal.pgph.0004161 (PMC11957299; doi:10.1371/journal.pgph.0004161)
Supplement: S1 Data — This file includes the following transcripts: •VL Patient In-depth Interview Transcripts: Verbatim transcripts of interviews conducted with VL patients, capturing their insights and lived experiences. •Healthcare Worker Key Informant Interview (KII) Transcripts: Transcripts from key informant interviews with healthcare workers, detailing their perspectives on decentralized care models for VL. (ZIP) [file pgph.0004161.s003.zip › HCW and IDI transcripts/patient interviews/Res 003_FACILITY 1.docx]

VL DECENTRALISATION STUDY

FACILITY 1

VL PATIENT/CAREGIVER INDEPTH INTERVIEW

**INTERVIEW**

Q1.`ayah’you start now (people shouting)so raised you voice I want to ask you a question how many days have your child admitted to this facility.

RES: I have stayed one week and this is the second week now………(church service voices)

Que: which date did you come….

Res: I came here on xx…..xx this month….yeah.i came here to the hospital, I took the child to lab. The blood were taken for test and after the test I was taken again to Emergency department where they return back everything then I was directed to the ward and that where I was told that the child is suffering from kalazar…okey….yeah.

Q2.What do you think is the cause of this disease?

RES:I came here and the child was diagnosed at the emergency and was suffering from kala-azar

Q3.What do you think causes the disease the child is suffering from.

RES: sources of water from river banks and water from wells and laggas….when they drink such water it creates a wound at the abdomen that causes enlargement of spleen.(hata akikula kijiko mbili anashiba)mmmh…..(people speaking)……mmmmh……(motor vehicle sounds)

Q4.describe for me symptoms the child started showing before coming to this facility?

RES: there were no symptoms showing because I thought was other disease until the child started feeling fever,body weakness,substantial weight loss and reaches place where the child didn’t even walk.eeeh what other…the child body changes the colour until I notice it is different disease…..even I used some water at some point to reduce the fever………….lets continue

Q5.Where did you learn from home about the condition the child is suffering from?

RES: The child started showing body weakness at home even eating the food didn’t had appetite without knowing the condition the child is suffering from because he was receiving medication at the nearest facility,I thought it was other condition until develops stomach and loosing appetite,sometimes the boy started crying.

Q6.Is there any member of your household you aware of that has suffered a similar disease?

RES :I can’t know because there some people in the community who have the symptoms of enlargement of the stomach but we didn’t know was such condition because no body give awareness about the disease or the condition that there is kalazar….mmh…(vehicles sounds)…………..mmmh….(little noises from people)…..(phone rung)….aahye…(children crying)………..

Que: Those people you saw in your family with the same symptoms like this of kalazar, did they sick any treatment in the hospital or not

RES: They were going to the nearest facility to seek medication and treatment..we have nariokotome facility that is supported by catholic that gives out treatment to people….

QUE: mmmh…ayah let me ask you another question.

Q7.Do you think this condition is a problem within the village you came from?

RES:Yes is a serious condition in my village because people neglect that requires people to understand that there are condition like this and that and decide to perform traditional method of massaging the stomach……(phone rIngs in vibration)…let me speak to this person at the phone..

Q8.let me ask you compared to malaria and other conditions like the one the child is suffering how would you describe the burden in your area?

RES: This disease is powerful because it kills and finishes someone blood level making it dropping down and someone feels unwell making the body feels fever of high rate also losing the weight……(sounds)……………….(papers blowing)

Q9.let me ask you also there at home whom do you think is most at risk of getting kalazar specifically on people above age and children?

RES:the person the disease can easily affects is small child depending on blood level and immunity weakness….(noises from people)…mmh…even now this boy his body has just started walking at the moment because last time all the strength of the body went out of the child.

Question on areas where is more prevalent?

RES: as for me I have heard an insect called sandflies from the anthills that spread the disease, it seems this flies also contributes in getting this disease….maybe doctors knows more about this.

Q10.tell me on how this disease you think it spread there at home like from one person to other?

RES: for me I cant know how it spread because the doctors are the only one knows how it spread and giving people knowledge on how it spread because in reserve areas people live together a times they fetch water together and eat together so I think that’s where I can say it spreads through blood…..(people mummering and sounds everywhere)

Q11. What do you think you can do to protect for you also and child from the disease?

RES: I say through knowledge and awareness that there is a disease call kalazar that if you see someone doesn’t know the condition is to tell him or her that to visit the hospital earlier before the condition persist to be diagnosed so it is only giving knowledge about the condition mostly on reserve areas like water pans the animals also drink the same water…….(noises from people)

Q12.briefly tell me how the disease was diagnosed when you came?

RES: they started by giving the child a drug like Panadol and injection before starting the kalazar one and the fever goes down but the his pressure goes down at night hours but for now his body has change it is not like the other days..mmh..so he feels well now…mmh…………….(phone message sound)

Q13.as for you briefly tell me how the disease is treated since the day you arrived here?

RES: when I arrived here the doctors are busy treating the disease and no way are letting at times also they give medication good way and also giving the direction on foods to give like milk,chocolates supplements and he has an injection once per day……..(sounds)…..

Q14.tell me when did the child become ill there at home?

RES: I see the health of child has change so I thought it is like everyday but the time has passed without knowing the condition the child is suffering and it started this year February 2023 cause I dentified the condition of the child is worsening for sometimes the child is diarrhoering and lacking appetite….(sounds from nearby people)….mmh..

Q15.tell me symptoms the child experienced before coming to the facility?

RES:I noticed some changes from the child like lacking of appetite and enlargement of the spleen and fever and weakness of the body…..(mmmh)

Q16.what symptoms made you feel the most need to visit the facility with child?

RES:I saw the child conditions is different like showing fever,diarhoea,body weakness and loss of appetite.

Q17.for how long did you notice the symptoms before visiting to this facility?

RES:I notice the symptoms many months ago,I told you I didn’t notice where is the problem at the body of the child,it takes many months I thought is malaria like other condition like for instance this one need the blood to be tasted to I dentify the disease the person has….mmh….(sounds of metal)…aah…mmh…(different kind of sounds from people and other objects)……mhh

Q18.so what made you wait for those months before seeking for treatment for the child?

RES:I would say I take the child to the health facility to seek drugs or tablets for treatment until when the boy got very worse that I decided to take this facility because I understand different diseases differs a lot…mmh..

Q19.so did you seek an alternative source of treatment before coming to this facility?

RES:I decided to seek treatment to one of the facility in lokitaung where the nurses diagnosed the symptoms and said it is kalazar and directed me to seek for care to lodwar referral hospital…and this disease if you cannot hurry it is dangerous…………(sounds)..ayh

Q20.what are challenges you experienced with the child as kalazar patients?

RES:I have experienced many challenges concerning this condition because there is no moving outside of the hospital and also no going back home and doctors advices not to walk on sunny day because it causes nose bleeding…mmmh…and I have seen many things about this disease..mmh.because you may also lose job that I did at home.

Q21.there is also another question asking on what factors motivated you to seek help outside of your household?

RES:I saw the child is going on a different direction of death because I see the child is worsening and also lack of money because our area is reserve you only rely on livestock to get money and for me I don’t have those livestock to sold to get money for transport…(children crying and playing sounds)I chose lodwar because I see the benefits of the facility……..(sounds)…`let me take this call’………………………….

Q22.what measures if any helped you during your process of seeking care.

RES:I have look the child getting good medication and food supplements that helps him a lot in gaining energy and care because the doctors are checking him daily.in some case bills have gone high but the boy is in good condition than the last place…..(children playing)…

Q23.among you household who decides on whether to seek care when the person gets sick?

RES :we decide with my wife and agreed that I would take the child to the hospital and told her to remain for me to take the child to the facility……mmmh.

Q24.were you aware you could get diagnosis and treatment for kalazar in this facility before the child get sick?

RES:yes iam aware and believe that I would get good medication and treatment at this facility because of trained health personnel and good treatment of kalazar condition.(Sounds of papers).

Q25.at households where do your community members seek helps for the condition of kalazar?

RES:for those at home or community it requires to get someone who would gave them knowledge on where to get good treatment so if you would decide to seek medication,for me also if I would get the opportunity of getting outside here to my place I would advice them to seek medication at this facility because I have known that there is good services at the facility.

Q26.Please tell me of your experience on health care you are receiving when you got to the hospital,what test were conducted on your child??

RES:at first the child was donated blood for test at the lab and waited for the results until it come with kalazar positive and the doctors directed me for stomach scanning to I dentify the problem on the boy stomach and since they start treatment….(sounds of people speaking)…..

Q27.what kind of..aah.. support are you receiving from family or relative member with the long hospital stay and kalazar treatment?

RES:no one,(metals sounds) it is only me and on the other way my wife also struggles on other hand to support me and the child for other food to support the child it is only my wife….

Q28.How much bill do you cost of stay reached and also the means of transportation ?

RES:eeh it is a lot of bill you cant even say because I have bill of drugs,bed days also and it is what make people to surrender to seek for medication or treatment.like for me if I want to depart it requires nearest to ksh.2000 to reach to my home nariakotome.

Q29.in considering the steps you took what do you think you would do differently now if you could start from the beginning?

RES:at this time the child is receiving treatment but in some time,lacking of money to buy fruits and also some meat for giving out strength to this boy cannot be found so it is challenging……mmh..people lack ways to access treatment….

Q30.what changes would you suggest to improve on VL care and access to VL care?

RES:for me i proposed the treatment to continue to seek ways on educating people about this disease..mmh(peoples sounds).to show challenges people face outside there.i suggest places like Turkana North to be installed good treatment commodities.

Q31.if any of your friends developed VL what would you recommend to them in terms of treatment?

RES:I would recommend them to seek medication ealier to this facility because it offers services to the people…..

Q32.are you aware of any past interventions for VL in this county of ours?

RES:Yes I am aware now because it a government it self it can hire health staffs to help people on other side of the county…….(sounds from people)

Q33.Kindly give more information about the barriers to access of VL diagnosis care and treatment?

RES: The most challenging one is the long distances when a person decide to seek medication to this facility he|she considers the cost and distances and bills for medication that’s what cause some people to leave people die. For me I saw the distances is long enough to seek medication because of poor background.

Q34.(Sounds from the children)please tell me what type of people have the greatest challenge accessing VL treatment?

RES:For me the disease is everywhere so it can get anyone including the small children and old people because it can easily get anyone because it is dangerous and kills.

Q35.What measures you feel should be put in place to address the barriers and improves access to VL services?

RES: For me to take treatment and medical services to the facility to nearest facility in regions at home(children crying)……….and also health staff to educate people about this disease.

Q36.(Sounds from people) now what can you tell me about the risk of developing VL once leaves turkana county and if you are aware of any available resources outsides turkana for VL care?

RES: when a person leaves outside the county probably in cases where people go outside to search for pastures so they may counter sandflies from anthills.

Q37.What do community say about the condition the child is suffering?

RES:to tell them dangers of the disease and ways of medication and also they say is dangerous and kills a lot.

Q38.what is the impact of community perceptions on VL care and diagnosis?

RES:just to tell them on good and dangers of the disease to understand.

Q39.what(children coughing) can be done at the community level to reduce stigma?

RES:this is from your side to take responsibilities to educate community because for us we stay together we don’t have incidences of separating anyone at community because is suffering from the disease and also creating awareness among the community on sensitization of the disease.

Q40.what is the best way to involve the community in strategies to combat and control VL?

RES:I may say we need to treat water that are drawn from the river or wells and cleaning the environment around the household for children to play to combat also on ways of getting the infections of the disease.

Que: so we have come to end of our interview session and thank you for your participation. Any question?

RES:for me iam suggesting for further clarification to help other people at the community.
