# Supplementary material for: Barriers and facilitators of care among visceral leishmaniasis patients following the implementation of a decentralized model in Turkana County, Kenya
Source: PLOS Glob Public Health. 2025 Mar 31;5(3):e0004161. doi: 10.1371/journal.pgph.0004161 (PMC11957299; doi:10.1371/journal.pgph.0004161)
Supplement: S1 Data — This file includes the following transcripts: •VL Patient In-depth Interview Transcripts: Verbatim transcripts of interviews conducted with VL patients, capturing their insights and lived experiences. •Healthcare Worker Key Informant Interview (KII) Transcripts: Transcripts from key informant interviews with healthcare workers, detailing their perspectives on decentralized care models for VL. (ZIP) [file pgph.0004161.s003.zip › HCW and IDI transcripts/patient interviews/Res 004_FACILITY 1.docx]

VL DECENTRALISED STUDY

PATIENT/CAREGIVER IN DEPTH INTERVIEW

**Interview**
QUE 1:How many days has your child been admitted at this facility?
RES:He has been admitted for 14days.

QUE 2:Tell me about the condition for which your child is suffering from?
RES:Kala Azar.

QUE 3:What do you think causes the disease your child is suffering from?
RES:I dont know something just caught him and made his body hot ,they said it is kala Azar.When the sun is down he becomes ill, the stomach start making some sounds

Que:The stomach does what..

Res: The stomach protrude.

Que:Did the Doctor tell you about the disease?

Res: The doctor have not tell hand anything rather than the child is suffering from Kalazar.

Que:What else do you think causes the disease?

Res:I said maybe it is food we eat or a fly from somewhere that causes it . Because there is nothing we know.In water that we all drink from the tap.I don't really know where it comes from,I just saw it. In the case where they have not tested to know where it comes from.

Que: Have you had about this disease before?

Res: I heard it from the other pastrolist, They treat it by cutting the protruding part of the stomach.

QUE 4:Briefly describe some of the symptoms experienced by a person with the Kala Azar
Res:He had fever,,his abdomen was protruding ,He lost weight,and his eyes became white.He lost blood think it took all the blood in the veins.

QUE 5:From Where did you learn about the condition your child is suffering from?
RES:I just  hear of people suffering from kala Azar.They are sick kala Azar.I heard of the rural people when they become sick,and they say the others are cut and at the past they said there is a karamojong,a pokot man called Aluru who ferries people to Matan and if there's a person who wants and knows on the town doings he goes there and  us we find in Kenya..."Phone ringing"...

QUE 6: there any other member of your household or community member you are aware of that has suffered a similar disease?
RES:Yes.I heard of some people sick ,There was a boy ..."phone ringing"..my father Ekeno son.

Que: You heard from your family members?

Res: Yes, In my family ,my father's home.He was treated the cultural way that you are cut.This disease went as far as making the boys stomach big and when it was about to kill him ,the blood came through his nose.He later died,he was always taken to the hospital and given drugs.They saw his belly and the x-ray was even done.

Que: So he is died already?

Res: He died, He was taken to Kakuma mission Hospital……..
     
QUE 7:,,,,,,,,,,,,,,,,Do you think the condition is a problem within the village you come from?
RES:It is a big problem.It is a bad problem.

QUE 8:Compared to malaria and other conditions how would you describe VL burden in your area?
RES:This disease is dangerous .”church service music”

QUE 9:Whom do you think is most at risk of getting kala Azar?
RES:Maybe the hospital can get,the doctor is the one who can see.

Que: I mean which category of individual is most at risk of getting this disease?

Res:Anybody can contract either old women or men anybody Kalaazar.

Que: Which areas are prone to this this disease?

Res:This disease is most found in the desert and rural areas.I don't know if it's in the water.In places like Kokorio and Moru a rengan.Moru Apolon is where I come from that is Loima.The place that is called Lokipoto.The JIE place.The days maybe like which date.The rainy season,it starts like malaria like it is the cold.In rural areas we say we don't really know but maybe it comes because of flies or the rainy water we take in the bush.Nobody knows.

QUE 10:Tell me more about the disease and how you think it is spread?
RES:I don't know this disease when it comes,it appears like malaria and it makes one I'll,become hot, hotness, so that's how it starts,I don't know let me say certain food , certain type of water brings.He becomes hot and we treat as malaria.I think when it's still small is when it brings hotness to the body,and making him sick mostly in the afternoon.And when it becomes big like my hand is when he loses weight and makes his belly big ,so we don't understand where it comes from.And to us we eat alot Herbal trees I don't know which one that treats that disease,how it comes ,is it water or what.
At it's start,it starts as a malaria UpTo when it gives birth to  it.

QUE 11:What do you think you can do to protect yourself and your child from the disease?
RES:I don't know what to do.That is why I just came to the hospital so that it is eliminated by the drug.I wish I knew that what type will heal ,i would have something to say.But now I am here for the medication."Ward noices"..."Music from around"...

QUE 12: Briefly tell me how the disease is diagnosed?
RES:They did an x-ray on him when we came here.

QUE 13: Briefly tell me how the disease is treated?
RES: He is given the injections on his hand and thighs.

QUE 14:When did you first become aware that your child is ill?
RES: I found out he is sick on august when his lost blood and his body and Belly protruded.

QUE 15:What are some of the symptoms you experienced on your son's body before coming to the facility?
RES:I saw death there of that disease showing on my son, i saw death coming.I saw on his body going to die and the belly protrude,and the body was about to finish.

QUE 16:What symptom made you feel the most need to visit the health facility?
RES:I just decided to bring him to the hospital to get life.And when I saw the first boy who was also sick was just like this UpTo when he nosebleed ans same happened to this one ,he nosebled and I decided to take a motorbike to here.

QUE 17: For how long did you have the symptoms before visiting the facility?
RES:I didn't bring him to the hospital immediately. I stayed home for three months.

QUE 18:What made you wait for those 90 days before seeking treatment?
RES:I saw that it was like the traditional disease,head disease ,the disease brought by winds  and measles.The disease became more and shows itself as this that is when I decided to come here with no knowledge that it was this.

QUE 19:Did you seek any alternative source of treatment before coming to the facility?How did you deal with the symptoms
RES:There is a hospital in Lokipoto that is even without doctors.'yeah' they did traditional way of healing.They said  you should slaughter a goat where that let's treat and bring back his heart.But then I just saw myself that it is not a traditional disease and it is that disease I saw from that person this and that is this.That is when I woke up to here.

QUE 20:What are the challenges you experience as a Kala Azar parent?
RES:The problem of that disease only.

Que: Is there any toxicities of drugs of this Kala Azar disease?

Res:There are no problems with this drug.It will bring back life to my son.He became worse.My heart is the one that is hurt .I even decided to boycott meals but this is now the times I get something to eat when he started getting the drug.But at first because it was a deadly disease and when you came and get to where their is life.Then the heart comes to normal at least you can have some water, there is no stress.

QUE 21:What factors motivated you to seeking help outside of your household for your illness?
RES:I saw the person become dead.I now decided to come and look for the medicine in a hospital like this.Some people directed to the other I regected them because I lack my son treatment on those facilities.Somebody came and said to me that ...it healed...it healed a person recently in this place.His relative.Even neighbors .That when...when I came and reached here somebody cheated that it is in a place called junction and I also went there thinking that ... because of the urgency on how he was dying.And when I went there that one told me that he send...that he has medication for people who...for the 2year old only..I don't have for 3 and half years.And there it was said that I should come to Lodwar.

QUE 22:What measures helped you during the process of care seeking?
RES: When I came from my I sold my  livestock to get transport so as to look for the medication in whichever place it was available.That is what helped me to get this medicine.

QUE 23:Among your household who decides on whether to seek or not seek care when a person gets sick?
RES: The husband decides on who take the patient to the hospital and who to remain at home……”church music”

QUE 24: Were you aware you could get diagnosis and treatment for kala Azar in this facility before you fell ill?
RES: Somebody told me and upon witnessing the person who was brought here being healed.He allerted me that life was here.

QUE 25: Where do your community members seek help for the condition your child is suffering from?
RES:They got ferried to  Uganda ..they are ferried by another person called Aluru. He piles them UpTo ten in number and take them In pokot Land a place called Matan.A hospital in Uganda.He takes them there...treats them and then get them back.He is always in those places.I saw that anything about Uganda...I am  going to  die in my land.
Que: How does the community say about the didease?

Res: They say that the disease is bad unless the sick person seek medication to survive.But if you just stay and say you should dig trees,and slaughter goats , someone dies and you remain with no goats.

QUE 26: Please tell me of your experience on the health care you are receiving?
RES:...in this hospital blood was taken.
Que: For how long did you wait for the results?

Res: ...when we came and they said waiting for blood. We slept and in the morning He was given drugs...mmmh..he came at night slept and then in morning given..They Gave it early.
They gave and after that it has made him to diarrhea blood.
They bring that drug ...when the doctor brings it is given through a drip,it is brought in a bottle and like water being injected to him Up To when it's over.
They also bring orals the yellow and red yellow...It's that sweet.
Sometimes back he was given through injection and it's given through the hand line .I also sometimes touch it to see how far it has reached..I once heard someone say if it breaks and blood comes out he will be okay.
That medication is good ..there is no it's bad side.
I am hopeful that my son will be healed.

QUE 27:What kind of support are you receiving from family and friends to help you cope with the long hospital stay and Kala Azar treatment?
RES: I come from far,when I come...I just call people to tell them..but only the father is the one who struggles.There is nothing am getting. “church service on going”

QUE 28:How much does it cost you as a parent to a kala Azar patient ,in terms of personal expenses?
RES: I used ksh.5000 only for transport.I came from my place and paid ksh 2000 to Kakuma and when I took another from Kakuma to the place called junction, I paid again ksh 2000.And took a car that to this place for ksh 1000.

QUE 29:In considering, the steps you took,what do you think you would do differently now if you could start from the beginning?
RES:The goodness that I see on his body is when my heart is fine and doesn't even think about that money .The good now,my heart is now okay.I can drink water,even eat,when i found that medicine.

QUE: What could you did differently before seeking treatment?

RES:There was nothing I would have done if I delayed to bring him here he  would have be dead now…………

QUE 30:What changes or interventions would you suggest to improve VL care and access to VL care ?
RES:.. What should I say in regards to the disease..it is just that They should find where this disease is coming from and close where it is coming from...what is caused..

QUE: Is there anything else you suggest to be improved?
RES: In terms of the accessibility of drug..they should add and  treatment accessible in Turkana.If the drug is not there this disease will finish people.

 QUE; How would you say about treatment part?

RES: The doctor should know how to treat and identify the disease even by seeing at it…….

QUE 31:If any of your friends or relatives developed Kala Azar what would you recommend to them in terms of treatment?
RES:I would tell them there is survival in this place because...There is survival in Lodwar because I was also in Lodwar .For the fact that I was told by someone that when I woke up to this place…….”mmmm”

QUE 32:Are you aware if any past interventions for VL in the county? RES:.

RES:Yes,I heard about people being sick of liver disease.The kala Azar is bad and even people talk about it when someone is sick prompting the sick to reject it.
It's bad to the person who gets it , it's a bad and deadly and when he/she goes to look for medication to whichever place he finds it....took it all if it's the medicine can remove it.
It's dangerous you wish for the drug to be available.

QUE 33: Kindly give me more information about the barriers to access of Kala Azar diagnosis care and treatment?
RES: it's problem is when on diagnosis..and also on diagnosis if it shows it's good.
The problem is when you've not taken him to the hospital to be seen and be tested,and if you just stay with him like that,it will just be felt it is kala Azar but not real.
Money scarcity...money can not be found for testing and when there is no Money ....
In regards to treatment...When you have money too, your treatment will continue knowing that you have money.

QUE 34: Please tell me what type of people have the greatest challenge accessing VL treatment and why?
RES:This disease Is much...it has caught the Amiis children,the son to Namuya.
.... children.. children are the ones affected,,the small children.

QUE: Why does it affects children mostly?
RES: The small children because when that person came...the first ones that were healed,by Aluru came and tested them and said it's kala Azar and ferry....when they only remain with big bellies.
Even the adults get sick but the children are many that this disease is affecting.
I don't know why it's the small children affected, now see the ones admitted in this ward are small children there is no adult.We don't know where they get it from ...the adults or From the small childrens food……”church service music” ….”mmmm”

QUE 35:What are the measures you feel should be put in place to address the barriers and improve access to VL services?-policy makers,healthcare authorities,NGOs etc.
RES:The government should find the medicine for it,and make it accessible....Bring them to the hospital.
The way to test ...the testing...the problem will be money.
When they say the drugs money...that will be hard.

QUE 36:What can you tell me about the risk of developing kala Azar once a person leaves Turkana county and if you are aware if any available resources outside Turkana for VL care.
RES:I don't where someone can get kala Azar...he /she will get because that disease is rampant outside there.he/she will get because kala Azar..that drug attacks many children and in anything we don't know where it comes from.
I have never heard on those small hospitals....oooh' people are sick in the rural areas there...I don't know in any other places about kala Azar.But even those patients admitted here are from other places so I said that this disease is also out there...someone has come from Katilu,another from Kibish and the other from Turkana West Naturturio...I said to myself that this disease is everywhere not only my place.

QUE 37:What do community members say about the condition your son is suffering from?
RES:They say he is sick of kala Azar ...'look for treatment'.
When sick...when sick like the way he has been,when the other person from another home sees ,he says its a wind disease but the aim is to eat meat, slaughter the goat.Then the other say it's measles.But when I realized,I saw someone die because of kala Azar,I talked to myself that this is the same disease by similarity but not tested..then I compared that it is that disease.That is when I came to junction and it become kala Azar the way my mind told me…..”mmmm”
That was what they said that it was Kala Azar.

QUE 38:What is the impact of the community perceptions on VL care and diagnosis?
RES:They make you delay .The people in the village tell you to every day stay behind...we should cut .. ooh' we should dig for some herbs making your mind relax and when your mind open the sick is down.

QUE 39:What can be done at the community level to reduce stigma?
RES:'what are we going to down Turkana... The medicine should just have to be find so that it will help People.

QUE 40:What is the best way to involve the community in strategies to combat and control VL?
RES: They should be visited by others...those in that village, talk to them it is this way,there is treatment for Kala Azar and this and that.
They should be visited by those who have known about that and others.

QUE: How should we alert them?
RES: They should let them know on the disease that has come in terms of testing,in terms of medication and that there is this type of disease it comes in this way.


Do you have any other question for us?
RES:''hahhaahha' I don't have anything to ask you,...it's just that about the drug ...am hopeful that this disease to help me ..I don't have anywhere else to go.
