# Supplementary material for: Barriers and facilitators of care among visceral leishmaniasis patients following the implementation of a decentralized model in Turkana County, Kenya
Source: PLOS Glob Public Health. 2025 Mar 31;5(3):e0004161. doi: 10.1371/journal.pgph.0004161 (PMC11957299; doi:10.1371/journal.pgph.0004161)
Supplement: S1 Data — This file includes the following transcripts: •VL Patient In-depth Interview Transcripts: Verbatim transcripts of interviews conducted with VL patients, capturing their insights and lived experiences. •Healthcare Worker Key Informant Interview (KII) Transcripts: Transcripts from key informant interviews with healthcare workers, detailing their perspectives on decentralized care models for VL. (ZIP) [file pgph.0004161.s003.zip › HCW and IDI transcripts/patient interviews/Res 005_FACILITY 1.docx]

VL DECENTRALISED STUDY

VL PATIENT/CAREGIVER INDEPTH INTERVIEW

**INTERVIEW**

Q1 How many days have you/your child been admitted at this facility?

RESPONSE: I have only slept in ward for three days. (Cough from the person who was passing)

Q2 Tell me about the condition for which you/your child are/is suffering from?

RESPONSE: We saw an Increase in the stomach size, loss of appetite and sleeping too much.

Q3 What do you think causes the disease you are suffering from?

RESPONSE: It is caused when eating fatty food, that’s our believe..mmmh eeh.

Q4 Briefly describe some of the symptoms experienced by a person with the condition/kalazar

RESPONSE: Loss of weight, eyes became yellow and the stomach increase in size. (Noise from nurses)

Q5 From where did you learn about the condition you/ your child is/are suffering from

RESPONSE: I knew about this condition from hospital and again from friends.

Q6 Is there any other member of your household or community member you are aware of that has suffered a similar disease?

RESPONSE: yes, tuberculosis, (TB)

Que: What symptoms did they have

-The kgs were down

-cough

- breathing difficulties eeeh.

Que: Did they seek treatment- from where and after how long?

YES, he seek medication from the hospital after two weeks.

Que: Was the condition treated successfully?

Res: Condition was treated successfully.

Q7 Do you think this condition is a problem within the village you come from?

RESPONSE: NO, because it is curable when action is taken immediately and is not transmitted from one person to another.

Q8 Compared to malaria and other conditions, how would you describe VL burden in your area?

RESPONSE: VL has got more burden than malaria because it kills shortly if not treated immediately.

Q9 Whom do you think is most at risk of getting kalazar?

RESPONSE: Young children because their immune system is too weak, this disease is common in reserve areas especially during rainy season....mmmh.

Q10 Tell us more about the disease and how you think it is spread?

RESPONSE: The disease is curable if taken to hospital in time and is not spread from one person to another even if you sit near to the victim.

Q11 What do you think you can do to protect yourself and your child from the disease?

RESPONSE: What I can do is to report to the facility immediately I see the symptoms from the body and again to avoid fatty foods ....mmmmmh..

Q12 Briefly tell me how the disease is diagnosed

REPONSE: The blood is removed from the body and taken to the laboratory for test and that is where they say this is the disease is in your body.When you go to the hospital,the doctor takes your blood and take it for testing and then he returns and say you are sick if Kala Azar...eeeh...

Q13 Briefly tell me how the disease is treated

RESPONSE: They inject you ....eeeh ...and some drugs if it's there.

Q14 When did you first become aware that you were ill?

RESPONSE: I started loosing appetite which was not normal at me, being sleepy most of the time, even during day time.I saw my eyes turn and my stomach became big....mmmh....

Q15 What are some of the symptoms you experienced before coming to the facility?

RESPONSE: I experienced loss of weight, stomach increased in size and breathing difficulties.

Q16 What symptom made you feel the most need to visit the health facility?

RESPONSE: Loss of weight.

Q17 For how long did you have the symptoms before visiting the facility?

RESPONSE: I have been with the symptoms for two weeks before I visit the facility.

Q18 What made you wait for (indicate number of days in 17 above) before seeking for treatment?

RESPONSE: The distance from my home to the facility was far, transport to go to the facility was also a problem and since you don't have a car, this force me to track from home to the facility which takes me three days, a distance of 120 kil0meters.If you start your journey you take two weeks on the way.

Q19 Did you seek an alternative source of treatment before coming to the facility

RESPONSE: YES, I used traditional medicine before coming to this facility and there was no any changes in my body ....mmmh... but instead the symptoms persist. I was told later that facility is the best place to treat the disease and that’s why I’m here right now."When you come from Lorengkipi on a car you can stay many hours on the road and sometimes you find there no cars ...and the disease keeps eating you up...mmmh..and that's why you can even get sick now and the car to transport you is not there,you just die eeeh..and now in my area there is only one car by the person called igoko and if that decides to stop at Losenyanait it's finished because our area is beyond that.And if it teaches at Lorengkipi it's just by luck.The hospital at Lorengkipi is there but ..the drugs are not there.Now this child that is where she started medication.We said maybe ,maybe but we saw that the medicines were not helping...so we waited for Ingoko's car when it arrived ...eeeeh ... it's like that in my area....eeeh.

Q20 What are the challenges you experience as a Kala azar patient?

RESPONSE: Due to loss of appetite, his body became weak because I was not eating, he also became anemic because my nose was bleeding every time and this makes me to go to hospital to seek for medication.

Q21 What factors motivated you to seek help outside of your household for your illness?

RESPONSE: The condition of my body became worst and this actually makes me to come to this facility. I did not want to die, yes ..

I choose to come to hospital because I knew that this is the best place where my case will be settled and I believed that when I reached to the facility, I will bounce back strong due to medication.

Q22 What measures if any helped you during your process of seeking care

RESPONSE: I decided not to give up in life, ...mmmmh..I have got hope that I will recover and that actually gives me confidence of seeking medication.

Q23 Among your household, who decides on whether to seek or not seek care when a person gets sick?

RESPONSE:... what?.… Husband is the one to decide if the victim will be taken for medication or not, he is the head of the family so his suggestion will be respected and followed.(ward Noises) In the process of looking for transport to reach to the facility, husband again is the one to decide if the goat will be sold in order for them to get the transport for going to hospital, if he says no, it remains that way. (Phone rings)A woman is not allowed to do that...yes...

Q24 Were you aware you could get diagnosis and treatment for Kala Azar in this facility before you fell ill?

RESPONSE: YES, I knew..I got this information from my neighbor whose child happen to be a victim of this disease a year back. My friends were the ones who told me about it, they advise and directed me to this facility where I believe I will be healed and I got medication..(, phone ringing).

Q25 Where do your community members seek help for the condition you are suffering from?

RESPONSE: There's no nowhere they just go the hospital to seek treatment.( Conversation within the ward)They believe when they go to the hospital you get healed.They say that if you go to the hospital,you get injections,given drugs and you get near to being healed.There will be difference between being at home and hospital...mmmmmh.....

Q26 Please tell me of your experience on the healthcare you are receiving

RESPONSE: My blood was removed and taken to the laboratory for screening and that’s where they came with the results that I’m suffering from this disease. I waited for the results for one hour.

I feel the treatment is well because since I started receiving this medication, my body have improved have gain weight, nose have stopped bleeding also. I have not experienced any negative effect from this medication. The medication is good. The treatment I receive from this facility is are injections and oral medication.

YES, I believe and trust on the treatment I’m taking. I believe that this treatment will heal me.

Q27 What kind of support are you receiving from family and friends to help you cope with the long hospital stay and kalazar treatment?

RESPONSE: I get money from my friends and family members, anytime they want to visit me, they all contribute some amount for me, then they give it to me when they come to hospital.

Q28 How much does it cost you as a Kalazar patient, in terms of personal expenses? (An estimate is ok). Probe on What are the expenses for/what did you spend on e.g transport to hospital, meals, medication, doctor/nursing fees etc

RESPONSE: Sometimes it cost me 2000-3000, I used the money for buying some fruits like: oranges, avocado and Apple, I use the remaining ones to pay the hospital bills thisdisease doesn't want something sour.... yes...you eat this to get strength.

Q29 In considering, the steps you took, what do you think you would do differently now if you could start from the beginning?

RESPONSE: What I will do is avoid fatty foods ,and keep it away from my body... because it's fatty foods that brings this disease to my body...mmmmh...

Q30 What changes/interventions would you suggest to improve VL care and access to VL Care?

-to protect people from getting VL

RESPONSE: There's is follow up for VL patients by the doctors and more machines for screening to be distributed to all facilities especially facilities that are located in area outside the town. bring drugs near us to prevent us from getting this disease.They treat the patients of kala Azar faster.mmmh.

Q31 If any of your friends or relatives developed VL, what would you recommend to them in terms of treatment?

RESPONSE: I will tell them to stop using traditional medicine but instead to go hospital to seek medication, telling them advantages of visiting the facility when you are sick,and the doctors have to bring drugs near us to prevent us from getting this disease.They treat the patients of kala Azar faster.

Q32 Are you aware of any past interventions for VL in the county?

RESPONSE: YES.

Q33 Kindly give more information about the barriers to access of VL diagnosis care and treatment

RESPONSE: The distance is far away from where I live, lack of transport of hiring the motorbike since the facility is much far from where I live. This make the medication to delay,

Q34 Please tell me what type of people have the greatest challenge accessing VL Treatment and why?- age, gender, underlying conditions etc

RESPONSE: Children of age 5-12 years because they normally have weak immune system .They don't have strong immune..

Q35 What are the measures you feel should be put in place to address the barriers and improve access to VL services?-policy makers, healthcare authorities, NGOs etc.

RESPONSE: It's just telling NGOs that they should help us by bringing Screening machines should be distributed to all facilities in order to identify the disease easily when the person become ill, more nurses should be trained and employed in order for them to fight the disease. (Unnecessary music)

Q36 What can you tell me about the risk of developing VL once a person leaves Turkana County and if you are aware of any available resources outside Turkana for VL Car

RESPONSE: Before travelling to maybe pokot or uganda, you yourself should know ,the doctor will not know.The doctor is not able to know where you are going...nobody will give you injection for going to another place.You should first confirm if VL cases is available in that place you would like to visit. You should protect yourself first before travelling.

Q37 What do community members say about the condition you are suffering from?

RESPONSE: They say that the condition is a dangerous disease and the people nearby are the ones who can diagnose it so that others will not get it.They say that the curable if it is treated in good time, others fear sitting near the patient VL, they say that if you sit near the victim, you will also be affected.(conversing among themselves)....hahahaha...

They also believe that if conditions will not be treated in good time, you may end up loosing life

Q38 What is the impact of community perceptions on VL care and diagnosis?

RESPONSE: They all believe that once the disease is screened out, and medication is taken seriously, so that the affected person will not die.

Q39 What can be done at the community level to reduce stigma?

RESPONSE: encouraging all the community members to love other, both victims and none victims and to be the brother’s keeper and helping each other when he/she is in need and not laughing to the patients of VL.Laughing on the sick can give someone stress and commit suicide....mmmmh...

Q40 What is the best way to involve the community in strategies to combat and control VL

RESPONSE: Educating the community members on the bad side of the disease and giving them good steps of handling the disease.They should guide them and train ... mmmmh....

I don't have any other questions.
