# Supplementary material for: Barriers and facilitators of care among visceral leishmaniasis patients following the implementation of a decentralized model in Turkana County, Kenya
Source: PLOS Glob Public Health. 2025 Mar 31;5(3):e0004161. doi: 10.1371/journal.pgph.0004161 (PMC11957299; doi:10.1371/journal.pgph.0004161)
Supplement: S1 Data — This file includes the following transcripts: •VL Patient In-depth Interview Transcripts: Verbatim transcripts of interviews conducted with VL patients, capturing their insights and lived experiences. •Healthcare Worker Key Informant Interview (KII) Transcripts: Transcripts from key informant interviews with healthcare workers, detailing their perspectives on decentralized care models for VL. (ZIP) [file pgph.0004161.s003.zip › HCW and IDI transcripts/patient interviews/Res 006_FACILITY 2.docx]

VL DECENTRALISED STUDY

VL PATIENT/CAREGIVER INDEPTH INTERVIEW

**INTERVIEW**

Q1: How many days has this child been in admission at this facility?

RESPONSE: He has never slept in the ward, he usually goes to pick drugs and come back home since he is still strong.

QUE:So he usually comes to the hospital and got injections?

RES:Yes ,he usually goes for injections then back and continue with other dugs which he was given.

Q2 Tell me about the condition for which you/your child are/is suffering from?

RESPONSE: He has fever all the time....all time and the second thing,he has less blood in the body,and the third thing he is nosebleeding.They nosebleed in the morning and at night...they nosebleed every time.The other thing he doesn't eat ,he just gets fever and can't eat.

QUE:He doesn't eat?

Res: yes doesn't eat ,he rejects food.And the belly is big ...that's what I said is this pregnancy or what ....(laughs)....About that belly,fever , Nosebleeding, doesn't eat became a problem in his body....mmmh....(greets the other patient).

Q3: What do you think causes the disease you are suffering from?

RESPONSE:what causes this disease mainly ,in turkana mostly people are sick .They say it's something from the anthills ,they found by doctors that is called sandly that bites people and we said it might be true because mostly who get Kalaazar are young children who looks after animals. Maybe it's that sandfly that brings that thing.

QUE:A sandfly

Res:yes a sandfly...eeh..from the anthills.

Q4: Briefly describe some of the symptoms experienced by a person with the condition/kalazar

RESPONSE: Loss of weight, dizziness, stomach increased in size and nose bleeding (noise from neighbors)

Q5: From where did you learn about the condition your child is suffering from?

RESPONSE: I heard from other people of the neighbors who were once sick kala Azar,we saw a child's stomach protruding ,and looks like he has never eaten or even eaten food because he looked malnourished ....they said it is kala Azar and when they touch the right hand side of the stomach they find protruded....you look at him having fevers..they said when he becomes sickly at evenings,he becomes hot,either in the morning...they said when he had fevers like that , that's what shows he has kala Azar.

QUE:And Nosebleeding?

Res: and nosebleeding too and the big belly.And when I found he has these symptoms that's when I knew it's Kala Azar.... that other children from the area were suffering from in the village....mmmh.

QUE:on the symptoms of this disease where did know about them?

RES:I got the information from the villages we stay in...in the village when you hear that someone's child is sick,either they say it's malaria,or that say whether is sick of kalaazar.They just know the reason why the child is sick of kala Azar because there's fever...eeeh ..his belly is big,...eeeh...and the nosebleeding and nosebleeding is the first thing and fever.

Que: so nosebleeding is the first sign that shows?

Res:...eeeeh... nosebleeding....and fever that comes and goes.

Q6: Is there any other member of your household or community member you are aware of that has suffered a similar disease?

RESPONSE: yes, (tuberculosis) the disease has the same symptoms with kala-Azar , patient of tuberculosis always cough blood while of kala Azar is nose bleeding.Its just cut like Kala Azar...And each of them is detected by touching the right side of the belly.

QUE: does they show the symptoms like that of Kala Azar?

Res: yes,it shows like kala Azar because it gives you fever, protruding abdomen...and sleeping with no appetite.

Que:what similar symptoms do they have?

Res:TB patients lose weight too and they all cough ....they got malnourished like those of Kala Azar.

They also bleed ..for Kala Azar they nosebleed but for TB they cough blood...

The persistent cought that doesn't go fast...they cough everytime.

Herbal medicine was the first dose they started but there was no positive response seen and then was taken to that Namorputh hospital patient to the hospital where he was treated and became healed.

QUE:For how many days? RES:They drink for alot of days...they said it's for six months .That's the time they treat kala....That TB.(Wind was blowing papers)...six months drinking untill when you are healed.(conversation within the ward).

Q7: Do you think this condition is a problem within the village you come from?

RESPONSE: yes it is a problem, not even the area but is a problem to all country, it could be better if we can find a way of removing this disease from the country, it kills in a short time when you try herbal medication and especially young children who looks after animals and this sand fly bite them when they go to rest under the hunt hill while waiting for their livestock(chats form the ward)..

Q8: Compared to malaria and other conditions, how would you describe VL burden in your area?

RESPONSE: kala Azar is worst compared to malaria because when you apply herbal medication to a patient of malaria, he\she will vomit and there is higher chances of him to be heal but when you apply herbal medication to a patient of kala Azar, the medication will heal the patient but instead it kills, kala Azar is the one that has brought a lot of burden in the area and even the country.

QUE: why do you think kala Azar is ban than malaria?

Res:This disease is bad because if nosebleeding ..you loose blood ...when the child starts nosebleeding...malaria does not make someone nosebleed. This disease is bad because when it takes away appetite for food,and when someone does not eat and is nosebleeding that person is going to die.(children playing)...when it gets to someone the person looks like HIV /Aids patient.The villagers will even think it's HIV...mmmh.

Q9: Whom do you think is most at risk of getting kalazar?

RESPONSE: people who look after livestock, this because when his/her livestock are grazing, most of the time they normally sit under the ant hills where this infected insect lives.Especially small boys and girls.Even this sick one has left livestock without a herder....mmmh .

Que:What times do a person gets this disease first?

Res:..when they find his sick or... it's on the herding side that's where someone can get this disease... sitting on the anthills..the dead anthills..that has ceased growth..there are those that are long.When the herder is looking after his livestocks decides to sleep under the anthill to rest.It bites there and that's where kala Azar comes...it was just resting or maybe playing...in the dead anthills that's where that disease comes from.

VL is found in desert areas and at most cases when there is a lot of rain in the area,this flies always lives in dead ant hills that are short in size

Q10: Tell us more about the disease and how you think it is spread?

RESPONSE: This disease is not spread from one person to another. When the infected person bites a person, he/she will stay with it until it is treated....mmmh..if it bites you you are the only one who will be sick. You can even share food with the infected person and you will not get it, you will not get the disease even by sitting near the infected person....mmmh..

Q11: What do you think you can do to protect yourself and your child from the disease?

RESPONSE: Staying away from ant hills and preventing young children from playing in the area where ant hills are many, burning all the ant hills around the area if it starts from growing around the home.In this rainy season people survive on some flieas find in the anthills ,so it's hard to protect because that is also food.I don't know to do if reaches that point...maybe the government should know.

Que;So that's the way you can protect yourself and your child?

Res...yes ..

Q12: Briefly tell me how the disease is diagnosed

RESONSE: Before the disease is disease is taken to hospital, people at home first tries herbal medication and when that medication fails to heal the patient, that’s when they decide to take it to the hospital, in hospital, the blood is removed from the body and taken to the laboratory for screening,they screen for several diseases until they find the exact disease.They take blood that's when they will if it's kala Azar.

Q13: Briefly tell me how the disease is treated

RESPONSE: Injections and the oral medicines, they first test for malaria before the suspected one, they also test for HB in order to know if you have enough blood.If you have less blood they will not start treatment on you...if you have malaria you will not start VL medication , malaria is first treated before VL medication is applied.When you finish the malaria treatment in three days... malaria is treated in three days.When you start VL treatment ..it is said that you will get seventeen injections. The one is given through the line and the other through injections.And again there are the oral drugs for blood and as they inject the two injections.And sometimes they also test your cough to know whether you have or not.If you have the cough you get treated.

QUE: so you said there are injections?

Res:yes, there are injections and oral drugs for blood and if he has malaria, that's when you are given for malaria and fever for the fever to go down....mmmh

Q14: When did you first become aware that your child was ill?

RESPONSE: The body started gaining weight and also nose stops bleeding, I experienced gaining of appetite. That’s when I came to knew that I am healed

Q15 : What are some of the symptoms you experienced before coming to the facility?

RESPONSE: stomach increased in size, fever, loss of appetite, nose bleeding and being sleepy at most time. ( car crossed the road) you see that this person is going to die.And on nosebleeding, you Saw that this person might ne sick of kala azar.Thats why he is nosebleeding and in Turkana you just touch the abdomen when they see it protruding.

QUE:Is it when you were still at home?

Res yes,I was not yet gone to the hospital....yeeeh...that's what we see...fever, nosebleeding And seasonal hotness of the body.And also not eating.

Q16: What symptom made you feel the most need to visit the health facility?

RESPONSE:Non-stop nose bleeding because I feared being anaemic. We all know that blood plays a big role in our bodies. ( young child playing)

Q17: For how long did you have the symptoms before visiting the facility?

RESPONSE: It reaches two months just seeing him experiencing the symptoms.The fever was non-stop.It took two months when cutting traditionally failed.

Q18: What made you wait for (indicate number of days in 17 above) before seeking for treatment?

RESPONSE:They said that Kala Azar is cut and when it's cut and blood poured ,it subsides that's why I waited .(greets a friend).When cutting and all that failed is when I came to the hospital.The facility is far from home, lack of transport to reach to reach to the facility. Livestock is being sold in to get money for motorbikes that's is the only source of transport here to NAMORPUTH.

QUE:Is there any other hospital around treating kala Azar?

Res:yes there are hospitals but they don't have treatment for kala Azar.Namorputh is the only hospital treating Kala Azar.The Lobei facility is there but no drugs... Lorengkipi and Lokwatubwa doesn't have the drugs.All these are the small hospitals same to Urum and Lokiriama but kala Azar treatment is only Namorputh.. That's where everyone from this area comes here....mmmmh.

Q19: Did you seek an alternative source of treatment before coming to the facility? Or how did you deal with the symptoms before visiting the facility

RESPONSE: Yes, at home we tried doing some cuts on him as you can see as people believed it heals the disease.

Que: What else?

Res:I also tried herbal medication which did not even heal him but instead it was leading my child to death.And when I heard someone brought his child to the hospital and got healed that's when I decided to bring mine too.

Que:Were there any changes you saw on your child body after using traditional interventions?

Res no ,it he became worse and weak.

Que?So that's when he got worse?

Res:yes.

Q20: What are the challenges your child experiences as a Kala azar patient?

RESPONSE: Loss of weight and swelling of the stomach and loosing of blood.At the period of treatment.. the injections are very painful.

Also before to the hospital,the feaces of the camel is put in fire and removed when it is too hot, it administered to the body until the blood comes out, this brings more pain in the body. Many cuts done using the knives ...that's the problem faced by kala Azar patient.Injections are painful that makes someone to stagger when walking..the pain of the seventeen injections.

Q 21: What factors motivated you to seek help outside of your household for your illness?

RESPONSE: I was motivated when I saw the child of my neighbor who became heal after being sick for long period of time.

Que:So that's what made you wait?

Res:That actually convinced me also to take my child to the hospital to seek medication.

Q22 What measures if any helped you during your process of seeking care?

RESPONSE: Livestock is what helped me most while seeking the treatment, I sold some of them in order for me to get money to take my child to the hospital since it is a far distance that needs transportation.The only livestock left after the drought.

Q23 Among your household, who decides on whether to seek or not seek care when a person gets sick?

RESPONSE: Mother because she is the one who is always around the child, like now the mother was the one who told me to wake up and take the child to the hospital.Also the father at most time is out of home looking for animals he isn't with the kids always.He doesn't know the is any problem at home because he comes home at night.The wife's is the decision maker.In terms of cash he is the provider and a woman has nothing to do with that.

Q24 Were you aware you could get diagnosis and treatment for Kala Azar in this facility before you fell ill

RESPONSE: Yes, I got this information from my neighbor, he told me that hospital is I can be assisted.Ans I believed that if I come to the hospital my child will get treated.

Q25 Where do your community members seek help for the condition you are suffering from?

RESPONSE: They seek treatment in PAG Namurputh heath center where they believe that when they visit the facility, they get treated.That's is the only place we take kala Azar patients.

Q26 Please tell me of your experience on the healthcare you are receiving

RESPONSE: The child is being treated well, I don’t see any challenges in terms of the treatment my child is undergoing.He is eating well.

First thing they is they interview you that way you are doing to me.Then you said that he has been sick for two months now and still not cured.You say the symptoms of fever,big belly and they say that those features are those of kala Azar.Then they take blood for testing for kala Azar.

Que :For long did you wait for the results?

Res:There are many things in the hospital.You come in the morning do this and that .. the results doesn't take too long despite the number of patients in the hospital but the test takes half of an hour (30 minutes).

Que:Do you the treatment the treatment your son is receiving will heal him?

Res:Yes,I believe in the medication my child is receiving, I have trust that he get heal the way am seeing now.He is okay..eats well...and plays with others ... and also his belly has subside.His nose has stopped nosebleeding.

Q27 What kind of support are you receiving from family and friends to help you cope with the long hospital stay and kalazar treatment?

RESPONSE: Yes, anytime they visit me in the facility, they normally leave me with some cash.They usually sell their goats in order for them to get money for me to buy some foods and also the blood drug.

Q28 How much does it cost you as a Kalazar patient, in terms of personal expenses? (An estimate is ok). Probe on What are the expenses for/what did you spend on e.g transport to hospital, meals, medication, doctor/nursing fees etc

RESPONSE: Ten thousand Kenya shillings, which I used to by some food stuffs to the child, this money is used to buy drugs also.It can even reach fifteen thousand.

Q29 In considering, the steps you took, what do you think you would do differently now if you could start from the beginning?

RESPONSE: the first step is to take the child to the facility whenever he gets ill and also to avoid giving herbal medication to the child.Those things were so painful to the child.

Q30 What changes/interventions would you suggest to improve VL care and access to VL Care

RESPONSE: Educating people on how to handle the VL in the community that this disease is this and that.And the other thing ...I think that's all... training is the only think we should protect this disease.

Q31 If any of your friends or relatives developed VL, what would you recommend to them in terms of treatment?

RESPONSE:The first thing I will tell to the person who has aquired this disease is to go to the hospital and seek medication and to avoid herbal medication.I once cut mine ,and used herbal medication but I was hurting him,what I would tell them is to go the hospital get tested and then treated.Anybody in my home,village or area... because everyone that has come to the hospital became healed.(aeroplane noises)

Q32 Are you aware of any past interventions for VL in the county?

RESPONSE: Yes, there is a time the facility receives the screening machines from the county and it helps people from the community

Q33 Kindly give more information about the barriers to access of VL diagnosis care and treatment (probe on the barrier that causes greatest problem)

RESPONSE: More nurses should be brought to in facilities to avoid patients staying in hospital for along time waiting for one nurse who is also occupied in other sector, health facilities are far away from where the people are living.

Lack of transportation to the hospital

Q34 Please tell me what type of people have the greatest challenge accessing VL Treatment and why?- age, gender, underlying conditions etc

RESPONSE: Young children of age 10-15 years because they are the ones who like playing in ant hills, this happens mostly when they are looking for their animals

Q35 What are the measures you feel should be put in place to address the barriers and improve access to VL services?-policy makers, healthcare authorities, NGOs etc.

RESPONSE: The first they should bring More screening machines to the facility, more facilities should built in arid areas to avoid death, medicine should be taken to arid areas.This treatment should be brought to Naturturio because people in that area do not know anything to do with drugs.So they still cut people.I wish they bring doctors,drugs so that they should uplift in effort to Kala Azar... because sometimes when you fine the doctor not there,you are told ,he will be back after two weeks,and those weeks aa you are waiting for the doctor to come ...a person looses blood and dies...or maybe you try bringing him to the hospital and he succumbs on your hands.

Q36 What can you tell me about the risk of developing VL once a person leaves Turkana County and if you are aware of any available resources outside Turkana for VL Care

RESPONSE: Educating them.

Q37 What do community members say about the condition you are suffering from?

RESPONSE: They say that the condition is curable ones it is taken to the facility immediately after showing the symptoms.

Que:what are people saying about that condition?

Res:They inquire if he/she has gone for treatment once they start showing the Kala Azar signs.

Q38 What is the impact of community perceptions on VL care and diagnosis

RESPONSE: They encourage you to go for treatment in the hospital.

Q39 What can be done at the community level to reduce stigma?

RESPONSE: Giving them health education through CHEWs and telling them not to laugh at those who are affected with the disease because it is not their fault to have the disease.So that they know when someone gets sick is not death.. someone can get heal and it's not transmittable.its eeh..I think it's just giving them training.The government should train people on this disease.

Q40 What is the best way to involve the community in strategies to combat and control VL

RESPONSE: it's goodness of keeping us in the know how on kala Azar,we are the ones whose people become heal and that we bring them to the hospital.Thats it's goodness when we talk of this things, should go to the community..by telling them when someone becomes heal they should bring to the hospital. The community should be included so that they should know the good and bad part of staying at home and coming to the hospital.

.
