# Supplementary material for: Barriers and facilitators of care among visceral leishmaniasis patients following the implementation of a decentralized model in Turkana County, Kenya
Source: PLOS Glob Public Health. 2025 Mar 31;5(3):e0004161. doi: 10.1371/journal.pgph.0004161 (PMC11957299; doi:10.1371/journal.pgph.0004161)
Supplement: S1 Data — This file includes the following transcripts: •VL Patient In-depth Interview Transcripts: Verbatim transcripts of interviews conducted with VL patients, capturing their insights and lived experiences. •Healthcare Worker Key Informant Interview (KII) Transcripts: Transcripts from key informant interviews with healthcare workers, detailing their perspectives on decentralized care models for VL. (ZIP) [file pgph.0004161.s003.zip › HCW and IDI transcripts/patient interviews/Res 007_FACILITY 2.docx]

VL DECENTRALIZED STUDY

VL PATIENT/CAREGIVER INDEPTH INTERVIEW

**Interview**

Q1.So let start now by asking many days your child been admitted at

this facility?

RES:for me it is like a year…mmmh..because she was living in reserve areas.

Q2ayaah...tell me about the condition the child is suffering from?

RES.I didn’t know until she was admitted to the facility and diagnosed because we thought it was malaria until the results shows it is kalazar at the facility.

Q3.for your thinking what causes the disease the child is suffering from?

RES. I’m not able to know the exact cause of this disease because there is so many ways of getting this disease..you can get this disease in many ways….mmmh…I’m not able to identify the exact way of getting…mmh.

Que.so have you heard anywhere to find this disease or somewhere or something that cause the disease.

RES. No,….mmmh…it is just in the hospital that I heard that this disease is caused by the sand flies that lives in the ant hills…mmmh,..sawasawa (child coughing)

Q4.When the child was admitted to this facility what symptoms did you notice in her body?

RES.It was this disease, what did you see in his body that made you to know that the child is suffering from kalazar….he was brought,.when he was brough fom reserve, he taken to a dispensary called Lokwatubwa and he was diagnised and that when I was told that it is kalazar he is suffering from…..kalazar was the first suspect even before a test was done.

Que: What was the child saying about his body or what was the condition that the child saw from his body that tells him that he is sick of kalazar? (sound from the sheep)

RES: She child did not say anything about his condition but the disease was already known…mmmh. Did he complain about the fever……No but headache but not most of the time but at large he complains on nose bleeding, she is bleeding too much….mmmh…. even in this process of medication. Bleeding….eeeh…. is the worst thing in this child…..What of his body, did you see any changes/…..Yes…the changes I saw from her body is that the body dried it was only bones that were appearing.

dQ5. (sounds from goats) Haya, if I may ask you from where did you knew that the child is sick kalazar? Where did you knew?

RES: I knew it in a dispensary called Lokwatubwa…….in a hospital called lokwatubo mmh….lowatubwa….mmmh….sreening machine is the one that bring it clear…hehe….viewed this disease…eeeh….some part of the stomach started swelling,…loss of blood in the body…..Which side is LOokwatubwa?.....it is beside that mountain……is there any hospital?..yes it is…eeeh.

Q6.(sound from blowing wind and goat) in your household or community has any member have suffered with a similar disease?

RES.no one has suffered in this community but people who lived far near the mountains and those who look after livestock are the ones who mostly get the disease……

Q7. Leets go a head, do you think this disease is a problem within the village you come from?

RES: Yes because if not treated, ……mmmh….one may end up loosing a lot of blood, stomach swelling and loosing weight…it is even heard to treat in that stage…mmmh…eeeh..the only thing infront is being dead.

Q8.lets continue, for your look how would you compare malaria and kalazar burden in your area?

RES. Kalazar is a big and dangerous disease compared to malaria because malaria is curable and even Christians may decide to pray to a person who sick of malaria and he may even end up becoming heal without any medication but in kalazar you will not heal.kalazar kills

Q9. Let’s go a head for your look whom do you think is most at risk of getting this disease kalazar? ......

RES. I’m not able to know the exact people that are at risk of this disease (sounds from goats) because all people move a round the area, they eat everything any howly….mmmh…they all go to the bush to look vegetables that usually grow there, they cook it and eat, even rain will find all of them outside their area and shower them with water, I’m not able to know the exact person this disease is waiting…oooh…..which time is someone risk of getting this disease? It is just the way I told you earlier, I’m not able to know...this symptoms always appear in the evening.

Q10. Let’s go a head, tell me more about the disease and how you think it is spread or the way the other person can get?

RES. When they share water……mmmh….when they share food mmmh not knowing it will spread to other person…..eeeh. So that when he is heal, the other one will start being sick.

Q11.What do you think you can do to protect yourself and your child from the disease?

RES.(other person speaking near the recorder) what I would say I have forget what to say but I prefer breaking of the anthills to flush a way the sandflies and also treating water for drink.

Q12.briefly tell me how the disease is diagnosed?

RES. the girl started by weakening the body and nose bleeding that’s why she was brought in to the hospital and she was diagnosed with the symptoms.

Q13.briefly tell me how the disease is treated?

RES. Mmh..at first facility in Lokwatuba the child was diagnosed with blood tested and also weight and found that she has loose weight until she even not have the energy to walk for long when she was referred to this facility for further medication when she is now getting treatment at namoruputh health centre………let continue,…mmmh

Q14.at first you told me the child become aware of the ill at what period?

RES.I said almost a whole year the girl got sick and sometimes she feels fever and taken to the facility thinking it is malaria until she suffers a lot..symptoms of the child fluctuates, during morning hours the child looks better, during evening the child seems to be sick. (wueeh)….

Q15.what are the symptoms that made you feel the child experienced before coming to the facility?

RES. we saw the situation is worsening she was feeling like nose bleeding that’s where the community understand that she is suffering kalazar (children voices)…it goes by time of the day..

Q16.What symptoms made you feel most to visit this Namoruputh PAG health center?

RES. The girl started weakening the body and thorough nose bleeding and also feeling headache…mh..sometimes she even not stand or walking.

Q17.For how long did the child have those symptoms before visiting the facility?

RES. the symptoms persisted almost a year until the girl suffered a lot that’s where body changes.

Q18.I need to ask this question what made the people to wait for long before seeking for treatment for the child?

RES. I’m saying it is like rudeness people at the reserve areas neglected the child who don’t have the mind of caring for the child, if it was here she has already get the care early before….our people who stays at reserve areas are still arrogant because they like neglecting things, they still think that traditional medication is good and that’s why this disease is large there, for me who is living in urban area nowdays, I know and understand that hospital is the best place to take any sick person. Even when you decide to tell them the truth they will not hear you, they are still rude.

Q19.from there where the child was staying did you seek any other alternative way of treatment before coming to this facility?

RES. No, the girl was only treated with traditional medicines with minds of simple conditions until the girl condition persisted day by day but the child continued becoming ill and the medication only help for some time not healing.

Q20.what challenges did the child experienced as kalazar patient there at home?

RES.for this girl it came in result when she was looking for domestic livestock there in the reserve areas when sometimes they play near the anthills and they cant know what will bite them.so they play because of childness and don’t have the place or resources for treating this disease…..(eeh sounds of silent)……

Q21.what factors motivated you to seek help from the village the girl stayed to this health facility?

RES.it is the health care worker in the facility..eeh.. identified the condition and went to notify the parents of the child that the disease has persisted a lot.so the community health worker at the village recommended the girl to seek treatment to namoruputh PAG health centre for further medication.

Q22.what measures helped you during the process of seeking care?

RES. The help of community health volunteer for administering the treatment to the girl to seek care at namoruputh PAG health centre because people at the reserve neglect the disease.

Q23.Among your household, who decides on whether to seek care when aperson gets sick?...mmh

Here at home…..?

RES. Here at home it is the responsibility of everyone to seek care when a person get sick because they know where to seek treatment but there in reserve areas until the nurse decides to seek treatment for them to help them and care for them they don’t have knowledge concerning the condition…mmh..

Q24.were you aware that the child could get diagnosis and treatment for kalazar in this facility of namoruputh?

RES.I didn’t know but when the community health volunteer in the village came with the child with that condition to namoruputh PAG health centre.

Q25.Where do your community members seek help for the condition the child is suffering?

RES. they seek to the Namoruputh facility because they understand that government is keen on delivering those services to the people but only for those in reserve areas they believe in cultural beliefs to treat the condition.

Q26.please tell me of your experience on health care you are receiving diagnoses to treatment….RES for me I see the treatment is good because the doctors give good medicines to people and for the girl is continuing receiving good care but I prefer also for them to take those medicines to people in reserve areas.

Q27.Iam also asking you what kind of support did you received from family members with long stay with the child when receiving treatment?

RES.(child crying)no one, it is the community health and her mother that brought her to this facility from the village.its only the support of the community health volunteer and mother remain home but when she referred here I took the responsibility.

Q28.there at home what expenses did you spend or personal expenses?

RES. (Interruption from the child) at the village the community health volunteer took the responsibility of expense to referring her to this facility with support from her mother,mmmh it is only the admission fee for the card was paid and the blood test expense and the doctors identified the cause of the condition.

Q29.considering the step you took what would you do differently now if you could start from the beginning?

RES. I would say that it is different because after she was referred here I took her to this hospital for treatment because this disease is a problem.

Q30.what changes or interventions would you suggest to improve kalazar care and access to?

RES. Kalazar medicine to be brought in large amount and people affecte with the disease to be helped in order for them to reach their basic need.

Que: Lets continue on your side on treatment how do you see it?..

RES.it is good process because I see on this girl when she takes the treatment the body remains good up to where you see performing domestic work here at home like fetching water and cooking food…she has even gained appetite. I don’t see any problem in this medication because since she started taking it, the response is good.

Q31.if any of relative or friend developed this disease what would you recommend to them in terms of treatment?

RES: I would advice him/her to seek treatment to facility…or to the health personnel for good medication…..

Q32.are you aware of any interventions in this county of ours about this disease?

RES…

Q. have you heard any?....

RES (insect interruptions) No i have’not heard anything concerning it.

Q33.kindly give me more information about the barriers to access of kalazar diagnosis care and treatment?....

RES. for me i say it is money for treatment when someone finds it is too much expensive and thus makes them to leave and assume the disease….okay…

Q34.please tell me what type of people have the greatest challenge accessing kalazar treatment even at the hospital,tell on age,gender….

RES. mostly it affects the people who lived at reserve areas because they lived in places where the anthills ….eeh..(person coughing)..mmh..are found and the small children who look after livestock are ones who are really affected by the disease for example like the you saw here earlier because they drink contaminated water and also food…let continues

Q35.what are measures that you feel should be put in place to address the barriers and improve access to kalazar services??

RES. I prefer more kalazar medicine to be brought in the facility, medicine that will not last in a short period….when it is about to be finished, it is the right for the hospital to order for other stock, nurses in charge also should be humble to the patients, talking to them politely.

Q36.what can you tell me about the risk of developing kalazar once a person leaves Turkana county and if you are aware of any available resources outside Turkana for kalazar care..

RES i would say when they go far like to the mountains they may encounter a sandflies that bites them any case they think it is normal until develops symptoms like of the disease and other case if they get sick they may end up dying at those places because they have no access to treatment services but when they arrived back home they may get treatment.

Q37.what do community members say about the condition the girl is suffering?

RES. Community members are praying God to give donors of this medicine strength and energy so that they can continue supporting their patients with drugs. And also people who lived near the hunt hills to destroy all the hunt hills surrounding them and buy medicine of destroying those flies.

Q38.what is the impact of community perceptions on kalazar care and diagnosis?

RES. To prevent people staying near the hunt hills and again it is not necessary that it is a must you visit hunt hill that you got this disease, flies from hunt hill can also come out and find you at home.

Q39.what can be done at the community level to reduce stigma and separation?

RES. in this community there is no such incidences because they understand the disease cannot be transmitted from one person to another unless you being biten by sandflies it is not like other conditions that recently kills people but you can share food and water with the person with kalazar without those perceptions,..

Q40.What is the best way to involve the community in strategies to combat and control kalazar??

RES.for me I prefer breakages of anthills..mmh..eehm..in the household and also inventing the best medicines that may prevent those anthills sandflies to come out to bite people and also treatment of the the designated anthillls to be invented to flush out those anthills mostly are found near our houses.okay I want to thank you for your participation and had you views from the beginning up to now for your benefits and the community,,….thank you.
