# Supplementary material for: Barriers and facilitators of care among visceral leishmaniasis patients following the implementation of a decentralized model in Turkana County, Kenya
Source: PLOS Glob Public Health. 2025 Mar 31;5(3):e0004161. doi: 10.1371/journal.pgph.0004161 (PMC11957299; doi:10.1371/journal.pgph.0004161)
Supplement: S1 Data — This file includes the following transcripts: •VL Patient In-depth Interview Transcripts: Verbatim transcripts of interviews conducted with VL patients, capturing their insights and lived experiences. •Healthcare Worker Key Informant Interview (KII) Transcripts: Transcripts from key informant interviews with healthcare workers, detailing their perspectives on decentralized care models for VL. (ZIP) [file pgph.0004161.s003.zip › HCW and IDI transcripts/patient interviews/Res 008_FACILITY 2.docx]

VL DECENTRALIZED STUDY

VL PATIENT/CAREGIVER INDEPTH INTERVIEW

**INTERVIEW**

Que: How many days have you admitted at this facility?

Res: 8 Days

Que: Tell more about the condition you are suffering from?

Res: I told you I came here as Malaria

Que:mmmmh "

Res: Feeling headache, I was given white tablets and yellow one. The Doctor also told am suffering from Kalazar.

Que: Mmm" Kalazar also is inside?

Res: yes it's inside with other infections, They transfused my Blood and sent me home for somedays and come for results.

Que:mmmh"

Res: I came back and I was being injected that is when I see changes on my body….i am still under medication.

Que: Did they confirmed that you are suffering from Kalazar?

Res:mmmm" They confirmed it's Kalazar.

Que: What do you think causes the disease you are suffering from?

Res:We men… we always create trouble cause even if you know there is Cows for witchcraft there, You take his or her cow to benefit your stomach.

Que:mmmh"

Res: So then, the owner of cow remain there cursing. It believe I'm cursed cause of it.

Que: "Mmmmh "

Res: And some people said, it comes from anthill

Que;mmmh"

Res: The red flies that found on anthill when bite it transmit Kalazar. And when treated at Hospital it’s a red flies bite, if not treated it is a curse.

Que: mmmh" Are there many red flies and anthill at your place?

Res: They are many, there this one of anthill that bite people, the other one is that….. that usually bite elephants of which when it bites a person he dies instantly. It is grayish in colour.

Que: Symptoms experienced.

Res: Stomach protrude, at left side while getting pain there.

Que: Is it Stomach alone you experienced?

Res: Headache every morning and every evening..so sometimes when the headache disappears you even start to eat food..

Que: From where did you learn the condition you are suffering from?

Res: I learnt it from reserve areas, even before coming to the facility here, there was

Kalazar. They used hot cow dung and burn the swelling part of the stomach.

Que: mmh"

Res: When they introduced medicine, we believe that medicine is better than using local Resources.

Que: is using hot cow dung effective?

Res: It is not effective, it create tattoos on body, some treated but good number of people lost there lives.

Que: Is there any other member of your household or community member, you are aware that has suffered similar disease?

Res: A number of community are suffering from the disease…… some seek medication at Namudat Hospital in Uganda.

Que: How long did they take while at the treatment?

Res: One Month and some days.

Que: was the treatment successful?

Res: They came Healthy like you.

Que: Do you think this condition is a problem within the village you came from?

Res: It is not a Problem

Que:mmmmh "

Res:mmmm" It was a problem when I did not seek for medication and now that am under treatment it is not a problem.

Que: What about for other people, is it a problem in the community?

Res: It is only a problem to a person who didn't seek for medication as I informed you earlier.

Que:mmmmh"

Res: mmmh" and for the person who usually take medicine for some weeks, he will get back home successfully.

Que: Compared to Malaria and other condition. How would you describe VL burden in your area?

Res: Malaria can be treated even with Herbal medicine, Kalazar is untreatable with local medicines until you seek treatment at the hospital…because it kills……

Que: Whom do you think is most at risk of getting Kalazar?

Res: Those living at reserve areas.

Que: Which category of individual?

Res: Mostly Children.

Que: Why does it affect children Mostly?

Res: Children because they take everything, They put more fats on body and also they take fresh milk direct from the animals before boiling.

Que: Tell us more about the disease and How you think it is spread?

Res: It comes to people without them knowing…….They kill some people and some treated with herbal medicine.

Que: How do you get this disease?

Res: I transmitted it through fresh milk that we drink and the fat part of the meat. But mostly the fresh milk.

Que: How the disease is diagnosed?

Res:The doctor touch my stomach

Que:"mmm"

Res:"eeeh" (He touches me.)

Que: With Hand?

Res: "eeeh" He tell me to lie down on bed and He touches the stomach, He then transfused my Blood.

Que:"mmmmh "

Res: He takes that Blood to Lodwar Referral Hospital to identify which kind of disease I'm infected with.

Que:"mmh"

Res: After some days I come back to the facility and they informed me I'm affected with Kalazar.

Que: "mmmh "

Res:He touches and tells you to step on X-ray machine.

Que: How the disease is treated?

Res: The Doctor informed you it comes like Malaria, He gave me tablets to relief headache for 3 Days.

Que: "mmmmh "

Res: When done with tablets I was injected and they write days for injections.

Que:"mmmmh " So you are still counting days for you injection?

Res: "mmmh" I'm still counting my Days for injection.

Que:"ooh"

Res:"mmmmh "

Que: When did you first become aware that you were ill?

Res: You will identify when you walk morning and find your body is dull.

Que: "Mmh"

Res: Not only dullness, I also felt headache, cough. I started treated it with herbal treatment.

Que: "mmmmh "

Res: Every evening I experience Malaria…. and someone asked "which kind of disease is this person suffering from?"

Que: All those times you are just taking herbal medicine?

Res: "mmmh " was taking the herbal medicine…. and I explained to them I feel the pain at my left ribs. They concluded that, this is Kalazar after touching it.

Que:"mmmmh "

Res: They bring more herbal products and they tattoo the swelling part of my stomach.

Que: What are some of the symptoms you experienced before coming to the facility?

Res: I lacked appetite, Headache, nose bleeding, Morning and evening fever, weakening the legs…then there you asked yourself..this is that condition…the people say..

Que: What Symptoms made you feel the most need to visit the health facility?

Res: General body weakness. I always walk long distances but since affected by this disease...I felt tiresome for short distances.

Que: So it is General body weakness that forces you to come to Hospital?

Res: "mmmh" was sleeping home doing nothing, so I had to seek for medication.

Que: What else do you think made you to seek treatment at this facility?

Res: That is all that forced me to come here.

Que: For how long did you have the symptoms before visiting the facility?

Res: For one month.

Que: What made you wait before seeking for treatment?

Res: Was taking Herbal medicine thinking that will see some changes..so the herbal medication made me to wait for so long thinking they will treat the condition…

Que: What else made you wait?

Res: Nothing else just local treatment.

Que: Did you seek alternative source of treatment before coming to the facility?

Res: "Mmmm" Before Hospital, we were using Herbal products. Was treated by the Herbalist till when the body get worse than before. I seek the treatment here.

Que: Did the Herbalist treat the disease successfully?

Res: They treat some but many people succumbed. Nowdays they do not even treat the disease.

Que: What are the challenges you experienced as Kalazar patient?

Res: No challenges I get, the only challenge I had is my Body changes.

Que: How does your body changes?

Res: Feeling pain in all body part, weakening bones, Feeling angry anyhow and sleeping frequently…..

Que:What factor motivated you to seek help outside of your household for your illness?

Res: I had that there is Kalazar medicine at this facility, Lodwar Referral Hospital and Uganda that priest take some patient. So I choose NPHC Facility because it is easier to access than other facilities. ("Nurse talking ")

Que: What measures if any helped you during process of seeking care?

Res: None of person helped me.

Que: Among your Household, who decides on whether to seek or not to seek care when persons gets sick?

Res: What decides… is how the disease take the person..on conditions is shows every morning and every evening….

Que:"mmmh "

Res: "eeeh" if the person is sick from morning till evening then the other day still sick. The mother can even decide to take his son or her daughter to the facility.

Que: What about on your community who decides on whether to seek or not to seek care when persons gets sick?

Res: These current days when the person get sick even the neighbor will remind to take him to hospital.

Que:"mmmh "

Res: Old days Fathers were the one to decide because they are the head of house.

Que: Were you aware you could get diagnosis and treatment for Kalazar in this facility before you fell ill?

Res: I was aware.

Que:"mmmmh "

Res: I was aware cause my neighbour had the same disease and he was treated at this facility.

Que: Where do your community members seek help for the condition your are suffering from?

Res: They seek at this facility unless those that are living at far areas like Loreng'kipi whose seek medication at Namudat Hospital in Uganda, taken by a priest.

Que: Please tell me your experience on the health care you are receiving.

Res: The treatment is good, the injection given is working good in body. I'm seeing some changes.

Que: What kind of support are you receiving from family and friends?

Res: No support just prayers.

Que:How much does it cost you as a Kalazar patient in terms of personal expenses?

Res: Total of Kshs 500, 50 for facility card, 200 Malaria treatment, 250 for blood transfusion.

Que: So you are done with facility bill?

Res: I'm done, only that maybe when I'm done with injection. …. I will pay Kshs100 for confirming whether the disease is treated successfully or still in the blood.

Que: In considering the steps you took, what do you think you would do differently now if you could start from the beginning?

Res: Before I sought medication at this facility, I could have died.

Que: "mmmmh "

Res: If not this facility I could have died, I'm thankful for coming to Hospital now my body is feeling well.

Que: What intervention would suggest improving VL care and access to VL care?

Res: Nothing can change only the government to provide medicine of the disease.

Que:"mmmmh "

Res: If there anything they should improve, they could have improved long time ago.

Que: If any of your friend or relative develop VL, What would you recommend to them in terms of treatment?

Res: I will advise him to seek for medication at Hospital. Hospital treatment is everything.

Que: Are you aware of any interventions for VL in the county?

Res: Not aware.

Que: Kindly give more information about the barriers to access VL diagnosis care and treatment.

Res: Not able to access because of Herding Livestock…so I thought just staying at home looking for livestock but it reached at time you can not withstand your body so you think to say I may die today and this livestock will remain so you decide to seek for treatment for the disease..so I was not able access treatment earlier because of herding livestock….

Que: Please tell me What type of people have the greatest challenge of accessing VL treatment?

Res: Youth.

Que: Why youth?

Res: youth, because most of them are pastoralist. They spent most time on bushes.

Que: What are the measures you feel should be put in place to address the barriers?

Res: I request the government to provide free treatment for Kalazar in every facility.

Que: What can you tell me about the risk of developing VL once a person leaves Turkana County and if you are aware of any available resources outside Turkana for VL care?

Res: Not aware.

Que: What do community members say about the condition you are suffering from?

Res: They took it as a bad disease, and prays for the affected patients to get right medication for it.

Que:"ooh"

Res: "mmmh" When you get back from the Hospital treated successfully, the community is happy.

Que: What is the impact of perception on VL care and diagnosis?

Res: No perception, the community took the VL patient like other ordinary people.

Que: What can be done at the community level to reduce stigma?

Res:They are not separating such kind of people in the community who have the VL cases so I have never seen If they have negative attitude towards them..like of TB,HIV..but they see as normal….

Que: What is the best way to involve the community in strategies to combat and control VL.

Res: By teaching people house to house…and awareness to them and introducing playing some competition on awareness to them of the disease..

Time:59 Min and 26 Seconds.
