# Supplementary material for: Barriers and facilitators of care among visceral leishmaniasis patients following the implementation of a decentralized model in Turkana County, Kenya
Source: PLOS Glob Public Health. 2025 Mar 31;5(3):e0004161. doi: 10.1371/journal.pgph.0004161 (PMC11957299; doi:10.1371/journal.pgph.0004161)
Supplement: S1 Data — This file includes the following transcripts: •VL Patient In-depth Interview Transcripts: Verbatim transcripts of interviews conducted with VL patients, capturing their insights and lived experiences. •Healthcare Worker Key Informant Interview (KII) Transcripts: Transcripts from key informant interviews with healthcare workers, detailing their perspectives on decentralized care models for VL. (ZIP) [file pgph.0004161.s003.zip › HCW and IDI transcripts/patient interviews/Res 009_FACILITY 3.docx]

VL DECENTRALISED STUDY

VL PATIENT/CAREGIVER INDEPTH INTERVIEW

**INTERVIEW**

Q1 How many days have you/your child been admitted at this facility?

RESPONSE: I have received six injections to date and I have never slept in hospital, I normally come to pick drugs and go back home. (children crying)

Que:He comes for injections only?

Res:yes,he comes for injections and back home....mmmh.

Q2 Tell me about the condition for which you/your child are/is suffering from?

RESPONSE: Nose bleeding, loss of weight, stomach swelling, loss of appetite he doesn't even drink water.And he also nosebleeds.Something has finished his body ...he looks like a person with kwashiorkor...mmmh..

Q3 What do you think causes the disease you are suffering from

RESPONSE: It is caused by flies that usually lives in hunt hills.

Que: sandfly fro the anthill?

Res: yes sandfly.

Q4 Briefly describe some of the symptoms experienced by a person with the condition/kalazar

RESPONSE: Since he got sick with this disease, he is not able to eat, he has lost weight like that of a person with kwashiorkor.HeI has fever most of the time , running to is a big problem, he also sleeps all the time and don’t drink plenty of water.

Q5 From where did you learn about the condition you/ your child is/are suffering from

RESPONSE: I have so many people in Lokichogio surfer from this disease, I came to learn about this disease from my neighbors and friends who have experience of this disease. They were taken to the facility for medication, he recovered.

Q6 Is there any other member of your household or community member you are aware of that has suffered a similar disease?

RESPONSE: No member of our family have suffered of the disease similar to this but one of my neighbor have been suffered of tuberculosis, symptoms are similar, loss of weight, cough and sweating at night

Q7 Do you think this condition is a problem within the village you come from?

RESPONSE: Yes, this condition is a problem to the community because it kills in a short period of time if not taken seriously.

Q8 Compared to malaria and other conditions, how would you describe VL burden in your area?

RESPONSE: VL is more dangerous because the victim lost lot of blood through nose bleeding and leading to death.

Que: This disease is bad than malaria right?

Res: yes it's worse.In malaria,the patient can even decide to take Panadol and become healed but Kala Azar has more burden than malaria.

Q9 Whom do you think is most at risk of getting kalazar?

RESPONSE: This disease likes Young children because they are always prone of playing in hunt hills not knowing that infected flies lives in it.

Ques:What factors increase this disease?

Res:It is not easy to see this flies because they rarely come out. This flies are always available during rainy season.

Q10 Tell us more about the disease and how you think it is spread?

RESPONSE: This disease is not like HIV/AIDS, HIV can be transmitted from one person to another but VL is not transmitted from one person to another.

Que:The way we are sitting like this can it spread?

Res:No you will not get ...it is only transmitted when an infected sand fly bites the person, you cannot get VL while sitting to close to the person who is infected.

Q11 What do you think you can do to protect yourself and your child from the disease?

RESPONSE: We an avoid playing to areas where hunt hills are surrounded and destroying all the suspected areas around the area. (Noise from the other patients)

Q12 Briefly tell me how the disease is diagnosed

RESPONSE: Blood is removed from the body and taken to the laboratory for screening, urine is also taken for test.They take blood from the hand.

Q13 Briefly tell me how the disease is treated

RESPONSE: Through injections and there is no oral medication,...eeeh ., fifteen injections, there is the one that is administered at the veins and the other ones at the buttocks. (Children crying)

Q14 When did you first become aware that your child is illl?

RESPONSE: I came to know he is sick when he was not in good condition when sleeping start being his habit, his body being weak every time. I just realize that he is not in good condition.His abdomen became big.

Q15 What are some of the symptoms you experienced before coming to the facility?

RESPONSE:Where I come from is almost 30 kilometers..mmmh.. I saw him loose weight, swelling of the stomach and loss of appetite.

Q16 What symptom made you feel the most need to visit the health facility?

RESPONSE: Stomach swelling is the one that shocked me mostly and that’s why I decided to look for medication.That is when I brought him to the lab to get tested.

Que: Is the disease Kala Azar?

Res:yes kala Azar.

Q17 For how long did you have the symptoms before visiting the facility?

RESPONSE: Almost a month, I saw it as a joke until the swelling of the stomach persist and that’s when I became serious.

Q18 What made you wait for (indicate number of days in 17 above) before seeking for treatment?

RESPONSE: I ignored it thinking it is malaria but when I came to the facility, the nurses first removed his blood to test for malaria, the results were brought and I was not sick malaria but instead I was told that I’m suffering from VL.

Que:Is there anything else?

Res:They said it's just malaria...mmmh.

Q19 Did you seek an alternative source of treatment before coming to the facility? Or how did you deal with the symptoms before visiting the facility

RESPONSE: I have been going to small facilities where they gave him Panadol for medication. That medication did not heal him at all. If not me coming to this facility I could have not even known that I’m suffering from VL.

Que: Is there any other place you went for treatment?

Res: No ...mmmmh.

Ques:Did the drugs from the facilities helped?

Res:No.

Q20 What are the challenges you experience as a Kala azar patient?

RESPONSE: I’m not experiencing any challenge, since he started taking this medication, nose bleeding has stopped, weight has increased, his appetite has increased, “if you buy a soda for him now he will finish it”. I’m getting better now.

Q21 What factors motivated you to seek help outside of your household for your illness

RESPONSE: The way he was going to die is what made me to go and look for medication, I feared death and that actually forced me to go and look for help.

Que:What made you choose this hospital?

Res:I chose to go to the hospital because I knew that is the only place I will get help and I believed that he will recover when I reach to the hospital.

Q22 What measures if any helped you during your process of seeking care?

RESPONSE: Presence of my money eeeh...the availability of money made it possible.

Even the injections that he is undergoing now I’m the one who bought it, advice also from friends helped me. ( noise from by standers)

Q23 Among your household, who decides on whether to seek or not seek care when a person gets sick?

RESPONSE: Both parents are the one to decide to if they will take the victim to facility or not, the child belongs to all of them.

Q24 Were you aware you could get diagnosis and treatment for Kala Azar in this facility before you fell ill?

RESPONSE: I was not aware that I could get treatment, I just knew that if they take blood they I will know.

Que: did you know if this child would get treatment here?

Res:I never knew....yes I never knew.

Res:yes, I knew that If I come here they will take his blood.

Que: Who told you that the treatment is here?

Res:I alone knew it.

Que:How is this child treatment?

Res:They give him injections.

Q25 Where do your community members seek help for the condition you are suffering from?

RESPONSE: They usually to Kakuma.

Que:where do your village mates go for treatment?

Res:They go to the hospital....eeeh..mmmh...

Que:So they look for treatment in the hospital?

Res:eeeh ...they go to either to the county or mission hospital.

Q26 Please tell me of your experience on the healthcare you are receiving

RESPONSE: His blood was removed and taken to the lab for screening.

Que:How long did you wait for results?

Res: I waited for one hour....mmmh.

Que:Do you think the treatment your child is taking will heal him?

Res: yes it will help him....yes..it will heal him.

Q27 What kind of support are you receiving from family and friends to help you cope with the long hospital stay and kalazar treatment?

RESPONSE: No.I don’t receive any support from friends or family members, advice is only thing they usually give me.

Que: so it's just you alone in the hospital?

Res:yes it's only me...mmmmh

(he coughed)

Q28 How much does it cost you as a Kalazar patient, in terms of personal expenses? (An estimate is ok). Probe on What are the expenses for/what did you spend on e.g transport to hospital, meals, medication, doctor/nursing fees etc

RESPONSE: I have used one thousand five hundred and fifty (1550) in paying all the tests that were done to him.

Q29 In considering, the steps you took, what do you think you would do differently now if you could start from the beginning?

RESPONSE: At least I got strength because my son has been helped.

Q30 What changes/interventions would you suggest to improve VL care and access to VL Care?

RESPONSE: I suggest health centers to be built in arid areas to avoid people walking for long distance seeking for medication in urban areas, this will reduce transportation cost. VL treatment should be taken to hospitals that are in desert also.They should build wards for patients to sleep instead of going to the hospital and back....mmmh.

This drugs should be taken to the facilities in the village like the dispensaries and when they diagnose you of that disease you are referred to any hospital near you.

Q31 If any of your friends or relatives developed VL, what would you recommend to them in terms of treatment?

RESPONSE: Somebody called Lokorio got sick of this disease, I told him to go to the hospital because that's where you will get medication.When he went ,he was diagnosed in clinic seven ,he took his drugs and now he is okay . There's no other problem.

Que:So is that what you can tell your friend?

RES:yes...he should look for medicines in the hospital.

Que:What else again?

Res:It's just to tell him to go to the hospital.

Que:If he want to survive he should go to the hospital?

Res:... mmmmh.

Q32 Are you aware of any past interventions for VL in the county?

RESPONSE: It's those times when the county hospitals got helped is when the people will get help because the medicines have been brought to the far areas it's just that the Turkana don't know.In places like Lokipoto.

Q33:Kindly give more information about the barriers to access of VL diagnosis care and treatmen

RESPONSE: Since this boy started treatment,I don't see any problem..mmmmh.

Que: How's the medicine?

Res:it's just entering into the bloodstream well....mmmmh.

There's nothing bad I can see like before.(voices from the ward)

Q34Kindly give more information about the barriers to access of VL diagnosis care and treatmen

RESPONSE: Young children ....mmmmh.

Que: Children of how many years?

Res:four years,five years or even six years.

Que:So it's the four years kids that get it?

Res:....mmmh..and five.

Que:Why them?

Res: Because they are the ones who like peeping through the anthill and eating the anthill soil.

Que:What about their immunity,is it strong or weak?

Res:it's weak...mmmh.their body skin.

(Children crying in the ward).

Q35 What are the measures you feel should be put in place to address the barriers and improve access to VL services?

RESPONSE: I will recommend the county to supply more screening machines to hospitals and also employ more trained doctors and distribute them to the facilities. By doing this, the government should bring machines and increase doctors.

Q36 What can you tell me about the risk of developing VL once a person leaves Turkana County and if you are aware of any available resources outside Turkana for VL Care

RESPONSE: Before travelling to a different place, you confirm if the treatment of VL is there or not.

Q37 What do community members say about the condition you are suffering from?

RESPONSE:This disease has become rampant. Due to loss of weight, others say that the conditions are for HIV/AIDS and they fear them too.

Q38 What is the impact of community perceptions on VL care and diagnosis?

RESPONSE:They should bring community health workers to teach people on this disease so they can stop stigmatizing them.

Q39 What can be done at the community level to reduce stigma?

RESPONSE: Encouraging them to love each other and to stop discriminating the affected ones but instead to give them good care.

Q40 What is the best way to involve the community in strategies to combat and control VL

RESPONSE: Training community health volunteers on health education (CHVS) and then the CHVS now will train the community members, they must be taught on how to stay in clean environment, all the hunt hills surrounding their village must be destroyed.

NB: I appreciated the patient for giving me his time, he also appreciated me for visiting him today, he is requesting the government to supply more equipment to the facilities and also build more facilities and also employ doctors.

I come from far places to this place why would they not bring this treatment to where I am.i come almost 40kilometers to this area and all that is money because of motorbikes.

I am so happy the machines are here not like the past when blood sample was transferred to Lodwar or Nairobi and then you stay for long before getting the results.But now everything is near the long distance is the problem .The remaining part is bringing the drugs to those facilities in the rural areas.Somewhere one doctor and the patients are many and when he is gone nothing happens,even when a woman gets to labour there's no one to help.
