# Supplementary material for: Barriers and facilitators of care among visceral leishmaniasis patients following the implementation of a decentralized model in Turkana County, Kenya
Source: PLOS Glob Public Health. 2025 Mar 31;5(3):e0004161. doi: 10.1371/journal.pgph.0004161 (PMC11957299; doi:10.1371/journal.pgph.0004161)
Supplement: S1 Data — This file includes the following transcripts: •VL Patient In-depth Interview Transcripts: Verbatim transcripts of interviews conducted with VL patients, capturing their insights and lived experiences. •Healthcare Worker Key Informant Interview (KII) Transcripts: Transcripts from key informant interviews with healthcare workers, detailing their perspectives on decentralized care models for VL. (ZIP) [file pgph.0004161.s003.zip › HCW and IDI transcripts/patient interviews/Res 010_FACILITY 3.docx]

VL DECENTRALISED STUDY

VL PATIENT/CAREGIVER INDEPTH INTERVIEW

**INTERVIEW**

Q1.Wait…let me start by asking you how many days have your child been admitted at this facility?

RES.i brought him here the last four days it is now the fifth day…so it’s the fifth day now and he is continuing taking the medicines…mmmh.ooh lets continue…

Q2.tell me about the condition for which your child is suffering from?

RES. the child is suffering from this disease called kalazar…….etid…mmh.. that’s the condition he is suffering and he started getting sick…mmh,, so what I would tell you is that this condition has affected my two children and this is the second one and it almost kills before coming to the facility and if it wasn’t referring to the hospital she was to succumbed but for real do you know where is loima…mmh,.. and now she is recovering in lodwar and again it is the same disease that is affecting this boy here.

Q3.tell me what do you think causes the disease the boy is suffering from?

RES. I’m told is cause by a sand flies from the hunt hills, a sand flies from hunt hills…..mmh……haya. Young children most of the time like playing in the hunt hills, they jump on top of the hunt hills and started peeping their eyes inside the hunt hill without knowing that there is a sand flies that bites..What else causes this disease, this disease?......the only thing that causes this disease is just the way I have told you, a sand flies from the hunt hill….mmmh…their no other cause

Q4.briefly describe some of the symptoms experienced by the boy or person with the condition of kalazar or what did you see in his body that made you feel like the boy is suffering from kalazar?

RES. The boy got sick with the symptoms like fever…mmh.. so we thought it is malaria condition because we take to a facility called tulabalany where he receives the medicines but the condition persist..so we realize..eeh its different disease..

Q5. From where did you learn about the condition your child is suffering from?

RES. I did not learn it from know where but instead I learn from my first daughter who I have told you was once sick this disease, I see now the symptoms are similar. My daughter was almost dead, she only survived when I took her to Kakuma mission hospital. When this boy became sick, we first suspect malaria because the boy has got fever, the body was trembling, he was diagnised and I was told that he sick kalazar.

Q6.Is there any member of your household or community member you are aware of that has suffered the similar disease?

RES. Yes they are sick…Q.so what shows them they have the disease or symptoms..?..RES..so they get sick there at villages and go to visit the facility and in that process the condition always affect someone in the stomach..(cough)… until it makes them weak enough to seek treatment to better facilities.

And also some people with condition seek medication to tulabalany facility thinking it is malaria but at the end it is different..Q,was this condition treated successfully?..RES..yes once a person got the treatment he/she got heal because that treatment heals the person easily.

Q7. Let’s go a head ,do you think this condition or kalazar is a problem within the village you came from??

RES. yes it’s a big problem…eeeh in the village than other illnesses because it drops down the blood level in the body….mmh and enlarging someone the spleen (people speaking around)..its like TB,,it is a big problem really so much..okay. we see this disease as a big problem.

Q8. If I may ask you, compared to malaria and other conditions,how would you described kalazar burden in your area?

RES.(…sounds from people) this disease call kalazar is bigger than malaria because malaria you can get treatment and you recover easily…aah…..eeeh.because even you when you have malaria you can get medication but for kalazar there is no any way you get treatment..it is aburden for real…….let’s continue…..

Q9.whom do you think is most at risk of getting kalazar?

RES. for me the small children are worst to get the disease easily..mmh..and for the adults you see differently when person got the disease…eeh

And for the place we lived the person may get the condition and given the traditional medicines and in some way you touch with hand and realized is kalazar(sounds from motor cycle)so you lack the type of treatment because the local medicines cannot treat but in old years someone was cut at the abdomen and reduce the disease…but for now it has come differently..mostly to us pastoralists..

And for the person may get at risk at any time when get in touch until you visit to the facility.

On factors this disease can be get at any time on different conditions and for our area in reserve areas we need to have those treatment at nearest facilities to cover up the distances we come from those places to the area.

Q10.Tell me more about the disease and how you think it is spread?

RES. When we get sick there at home, we usually use traditional medicine thinking that we will get ill, we thought it is just a common disease, we came to know about this disease when I brought this child in this facility, his blood was removed from his body and taken for test, the results were later brought and I was told that the child is sick etid

Q11.what do you think you can do to protect yourself and your child from the disease?

RES. for me doctor I would not say anything because where we lived we encounter different kinds of food and water from different sources for example the succumbed goats we eat their meat and also the fruits from different trees in the bushes..i think for your side you may know the ways to protect from this condition.

Q12.briefly tell me how the disease is diagnosed or how was the boy diagnosed with the disease?

RES. The child become ill at home and was first diagnosed at tulabalany facility in loima and given some drugs hoping it is malaria but the boy condition persisted with fever and continues in worst condition but I touch the boy and went back to the facility to tell them this condition is different so what the do the same day we came across the doctors who have come an outreach and they diagnosed the boy and tested the blood to find the kalazar condition and directed me to refer me to this Kakuma hospital and for that what we request from you to look for ways for us to get help in those villages without coming all along up to here….we want drugs of kalazar to be taken to facilities that are located in reserve areas to reduce cost of transportation.

Q13.briefly tell me how the disease is treated?

RES. The boy getting injections, one in the hand and the other in the leg.Q. how many times a day? RES. twice a day for the days he has been prescribed to take injections for 17days and for sure people like us to come all the way up to here is long distance seriously, we urge the government to build hospitals in reserve areas also and put medicines and nurses their so that we can access them easily. (sounds from motorcycle).

Q14.when did you realize first that the child is ill?

RES.I become a ware a month ago when the child become ill that made me to seek the treatment up here because of this kalazar, I did not take the boy to the hospital immediately because I thought it was any normal illness and that’s I wated one week staying at home. The persist and why I brought him here. (sounds from people and papers)…

Q15.what symptoms you experienced before coming to the facility?

RES. The boy become ill and severe fever persisted making the boy weak .

Q16.what symptoms made you feel the most to visit the facility?

RES. The boy illness persisted because he was very sick and fever all over the body and body becomes weak.

Q17.for how long did the boy have the symptoms before visiting the facility?

RES. The symptoms in the boy last for almost a month before deciding to visit the facility. mmh

Q18.what made you wait for a month before seeking for treatment?

RES. for me there was no any genuine reason for staying but only lacking for money for transportation for our side we have motor cycle..mmh, you can only hire motorcycle if you have money, if you don’t have money you track or go by footing which it took more day to reach the hospital.

Q19.did you seek any alternative source of treatment before coming to the facility?

RES. No other way I treated the boy because when he becomes ill I take to facility and given dosage thinking it is malaria as usual..aayah when the doctors get the condition and and that is the time he referred me to this facility,

Q20.what are challenges you experienced as boy got kalazar?

RES. For sure doctor our place is very far and at home we have livestock to keep so when we came here with my wife because of this boy conditions so all those livestock will be lost and everthing will be stranded because of our stay here like serious.. mmh its remains like that. mmh…eeh

Q21.what factors motivated you to seek help outside of your household for the child illness?

RES. What motivated me seek medication here is for my girl reason,,,mmh because the time she becomes ill we stay with her for so long without knowing her conditions and giving traditional medicines hoping for treatment until we refer her to hospital and for really she got the treatment..the girl..so what made me to take this boy here at this hospital was similar to that of her sister…mh. (sound of a phone ringing)…

Q22.what measures if any helped you during your process of seeking care..?

RES.so what measures doctor will I say because the only thing I’m here is because getting treatment for the child because I understand that the hospital is giving good treatment of the condition.

Q23.among your household, who decides on whether to seek or not seek care when a person gets sick?

RES. doctor,, in the process where the child got sick at home it’s the responsibilities of both me and my wife…mh. to seek treatment to the good hospital no matter of what is available..mmh. hospital is the only thing that is helping me because of the treatment my boy is receiving.

Q24.were you aware you could get diagnosis and treatment for kalazar in this facility before the child fell ill?

RES. i was not aware (the mother of the child speaking) I was just going by Gods way..mhm..hopping I would get treatment at this facility and no one showed or directed me to seek treatment here,,mmh

Que: Who told you to you to come to this hospital

Res: No one have told me…ooh…I guesed myself….i was not sure if my child will get medication since I have not been in that hospital, its just by the grace of God.

Q25.from there at home where do your community members seek help for the condition the child is suffering from?

RES. there is no other place they seek treatment but for us in the village when someone becomes ill of the condition we were cutting the abdomen until it causes people to die until sometimes in our village there is a car that carries people with the condition to seek for treatment to Uganda country(mother speaking)and when they get the treatment they ferry them back to the village..mmh there is no any medication because we didn’t have the knowledge or idea…mmh..

Q26.please tell me of your experienced on the healthcare you are receiving when you got to the hospital and what tests were conducted on you child for them to determine the condition?

RES. i told earlier that the boy was diagnosed and tested at tulabalany facility in loima where those outreach doctors diagnosed him to an extent that blood were taken and the results showed he was suffering with kalazar conditioned and referred me to this Kakuma subcounty hospital.(mother speaking)the referral letter they give to me is the one that directed me to this hospital.

And on the other way how is treatment on side effects?RES.so when he got the treatment he is continuing recovering, the fever that he feels has disappear it is not such much like the beginning,mmh

Q.do you feel that the medication that’s being given can heal the condition? RES. when he was given the injection he start recovering by the way he is not feeling fever now..ah..so I am monitoring also on his movement when given the injection is not that really bad.Q.do you have any follow ups of care when you leave this facility?RES.NO follow up for us we only come daily to receive the treatment and went back to where we stay.

Q27.do you have any kind of support you are receiving from family or friends to help you cope with the long stay in receiving treatment in this facility?

RES.NO kind of support from family members or friends(motorbike passing) may be if there was any had the heart of giving or supporting in terms of transportation it will have been easy but for now I don’t think so…mmh.

Q28.how much does it cost to you as child with kalazar in terms of expense?

RES.I have not spend any money but when I came here we pay only for the admission card..mmh. we bought that card. mh..its only that..Q. what of meals?...RES..no anything..Q.in terms of transport did you spend anything???RES.no any expenditure…okay

Q29.in considering the steps you took what do you think you would do differently now if you could start from the beginning?

RES.in my mind I had two options to decide even the distance Is long from home so whom do I take or tell the conditions to support because I have to take the child to the hospital to get treatment until the time the treatment ends and for that if no means I will start walking towards home but in my mind I want the boy to be heal and get proper treatment,.it is just like that…

Q30.what changes would you suggest to improve for kalazar care and access to kalazar care?

RES.so what I would tell you is that the treatment here in Kakuma to be transferred to our places because I come from loima so what iam suggesting to invent the treatment to our area facilities even when someone get the condition it will be easier to get treatment as early as possible, mh..

Q31.if any of your friends or relatives develop this disease what would you recommend to them in terms of treatment?

RES. i only say because my first daughter got sick of the condition and I came with her to seek medication until she get treated and for this one also I would also get his medication and go back home with him so if in any case that the relative or friends develops such conditions I would recommend him/her to seek treatment to this hospital cause even I experienced such way,..mmh

Q32.are you aware of any past interventions for this disease?

RES. I’m not aware in any way because this is the first time I have heard from you than anywhere else…

Q33.kindly give more information about the barriers to access of kalazar diagnosis care and treatment?

RES. So what I would say is that we need treatment near us..mmh. because this is long distance of looking treatment like lodwar,Kakuma,Lokichogio,but when you don’t look for treatment you would succumbed to this disease because of lacking those resources near us,so in my two options it need to have money for transport to seek care but if you don’t have it become difficult….so I have that options?

Q34.Please tell me what type of people have the greatest challenge accessing kalazar treatment?

RES. From home or where?...so what I would tell you is that this disease is a dangerous disease as I had told earlier that all the people in households came across this condition before they know the treatment on this region they were always ferried by a car to Uganda to seek treatment for the condition so some of them succumbed to death because of the condition and for others who get treatment they get heal and come back home stronger..aah..mmh..so many people get in touch with the disease at the village and are there like that….

Q35.what measures you feel should be put in place to address the barriers and improve access to kalazar services?

RES. The only way I proposed or suggest is providing medical support to the facilities near the people..aah because to prevent this sandflies from biting people and cause the disease and also giving us mosquito nets in large number to protects from bites with either those insects..mmh (mother speaking in other side) also to continue supporting the community with cash transfer money to support on transportation issues when someone need or seek treatment to distance facilities.

Q36.what can you tell me about the risk of developing kalazar once a person leaves Turkana county and if you are aware of any available resources outside the Turkana county for kalazar care?

RES. there are no any available resources outside there because if get the disease or the condition he/she may suffer…eeh..so..mmh,(sounds of motorbike) so the only treatment is here at this facilities within the county,(sounds from motorbike)so even in turkana culture it was only cutting of the abdomen, for now this is the right medication or treatment.

Q37.what do community members say about the condition you son is suffering from?(Sounds interruption of both the motor bikes)

RES. There at home community members expects the treatment to be available the way it is now in occasion you feel sick or ill you go and treated immediately..thats the expectation in our belief for now.(person calling another person).

Q38.what is the impact of community perceptions on kalazar care and diagnosis?

RES.(phone ringing)the way people belief in turkana is coming to hospital to seek for treatment for the condition (respondent laughing)so a person need only medical attention no any thing else.if to test blood for the condition he/she must accept it…

Q39.what can be done at the community level to reduce stigma?

RES. In this community recently there is no kind of stigmatization unless like past years or in old ages when aperson becomes ill or sick in the neighborhood so the person tries personally treating the condition without even others coming to visit…

Q40.what is the best way to involve the community in strategies to combat and control the kalazar condition..?

RES. So what I would say is that all those process the doctors and other health personnel are responsible for that to involve the community on ways to drink good water and give methods of eating good food and other things. so that’s the their responsibilities so what I expecting for us is to give the medication near to us to help us more easily in condition when the person get sick he/she may seek treatment near to those facilities for example now here at Kakuma where we are receiving the treatment.so that’s what I would say,.yeah..mmh..earlier I told you that this all process it needs the guidance by health personnels…..eeh..mmmh so I want you to tell me on ways on how we combat the condition?RES.so I’m requesting the treatment to be near us in those facilities like tulabalany in letea ward in Turkana west,..so I take this moment to thank you for your participation since we started because we have already recorded what we talk and thanks so much for your time.
