# Supplementary material for: Barriers and facilitators of care among visceral leishmaniasis patients following the implementation of a decentralized model in Turkana County, Kenya
Source: PLOS Glob Public Health. 2025 Mar 31;5(3):e0004161. doi: 10.1371/journal.pgph.0004161 (PMC11957299; doi:10.1371/journal.pgph.0004161)
Supplement: S1 Data — This file includes the following transcripts: •VL Patient In-depth Interview Transcripts: Verbatim transcripts of interviews conducted with VL patients, capturing their insights and lived experiences. •Healthcare Worker Key Informant Interview (KII) Transcripts: Transcripts from key informant interviews with healthcare workers, detailing their perspectives on decentralized care models for VL. (ZIP) [file pgph.0004161.s003.zip › HCW and IDI transcripts/patient interviews/Res 011_FACILITY 4.docx]

VL DECENTRALISED STUDY

VL PATIENT/CAREGIVER INDEPTH INTERVIEW

**INTERVIEW**

Que: "Birds noise" How many days your child have been admitted at this facility?

Res: When I came here, at first the child was admitted 9 injections. I get back home when done with injection. Then I came here on Monday last week.

Que: you came on Monday?

Res:"eeeh"

Que: So it is like you have stayed here for a week.

Res: Yes, one week and half because of this.

Que: Tell me about the condition for which your child is suffering from?

Res: He is suffering from "etid" Kalazar.

Que: What do you think causes the disease your child is suffering from?

Res: even me I don't know what causes it. It just appears at my son. It started as Malaria and when I came here, I was told it is caused by sand flies from anthills…

Que: Here in facility they said it is caused by sand flies?

Res: "eeeh"

Que: What the community you are living in said about the cause of the disease?

Res: They don’t know the cause of the disease.

Que: Does your child grow with the disease?

Res: No, He felt sick when he is grown.

Que: So you Don't know the causes of the disease?

Res: Maybe dirty water and dirty food we usually take at reserve areas.

Que: What are the symptoms identified on your child illness?

Res: He lack appetite, and when it get at night his body get warmer.

Que: "mmmh"

Res: And his body changes, looks different as he was before getting illness.

Que: "mmh"

Res: And also I touched his stomach and found that thing there.

Que:"mmm"

Res: After identify we did all Turkana traditions things but all fails.

Que: "Oh" And from where did you learn the condition your son is suffering from?

Res: It is believed that sandflies brings the disease.

Que: Who told you that sandflies causes it?

Res: I learned from other people affected with the same disease.but mostly I learned it from this facility.

Que: Is there any other member of your household or community member you aware that has suffered a similar disease?

Res: On my family side None is sick.

Que:"mmmh"

Res: This boy here only was sick.

Que: What about other community members including neighbours?

Res: The only person I know she is my neighbour son. Who is here with me at facility.

Que: What are the symptoms do his son experienced?

Res: She experienced the same symptoms of my son.

Que: "mmh"

Res: She also asked me how my child is feeling.

Que: Did you asked her about his child condition?

Res: She also tells me how his child is feeling. Like He always feel hot during evening, lack appetite,"child talking"He drinks water alot.

Que: How long does her child stayed at Hospital?

Res: Two weeks

Que: Is there any positive changes on her child body?

Res: The child is fairing better now, He came here in bad condition since he get medication, Even food he is taking now…….

Que: Do you think this condition is a problem within the village you came from?

Res: For me is not a problem.

Que:"mmmmh "

Res: It is a problem only when you lack treatment.

Que:Compared to Malaria and other condition, How would you describe VL burden in your area?

Res: Kalazar is tough.

Que:"mmmmh "

Res:Because when someone affected with Malaria, takes Panadols, it reliefs pain on his body. And also it can be treated with other local treatment than this one of Kalazar that needs a specific treatment "Birds noise and other patients talking behind "

Que: Whom do you think is most at risk of getting Kalazar?

Res:For me I can't say it is only children get this disease, this disease affects every person including adults. "Coughs "

Que: Which category of individual is most at risk of getting this disease?

Res: Children under 12 years.

Que: Why does it affect children Mostly?

Res: They consume everything thus affects them.

Que: What about the adults that don't consume dirty things?

Res: It might be transmitted to them through sandflies bite and uncovered food that they take.

Que: Which places is most at risk of getting Kalazar?

Res: Mostly Turkana people living in reserve.

Que: Why does the disease attack those people living in reserve?

Res:As I told you earlier, it is because of dirty things we usually consume.

Que: Which kind of dirty things?

Res: like for example our structure are poor. When the wind blows, it blows to our food and our drinking water is at open places that every animals uses it including dogs.

Que: "mmmmh "

Res: That is what brought disease to the body.

Que: Tell more about the disease and how you think it is spread?

Res: "child cries " "phone ringing "it might spread through wind blowing and transmitted to other person.

Que: So then I can get it from your son because I'm sitting towards wind direction? "Laughter "

Res: Even me I don't know how it get to other person.

Que: What do you think, you can do to protect yourself and your child from the disease?

Res:"child cough "Maintaining hygiene at the compound. Cooking food well and boiling drinking water.

Que:Tell how the disease is diagnosed?

Res: Blood testing at the lab, and Hand Touching at the stomach.

Que: Tell how the disease is treated

Res:,,,,,"patients talking " by giving medicine.

Que: Which kind of medicine?

Res: not tablets, injected one. "Child talking "

Que: When did you first become aware that you child is ill?

Res: I knew it long time ago.

Que: When did it started?

Res: It started on June….. I thought its a Malaria. When I take to the dispensary they say there is no disease in blood.

Que:What kind of symptoms you experienced before coming to the facility?(background noise)

Res: The body skin changes to pale, Lack appetite, sleeping frequently.

Que: Why does his hair is yellow?

Res: It is the disease that turn his hair to yellow.

Que: What else did you experienced?

Res: Nothing else…But the child was urinating concentrated urine, Weight reduces to 4kgs. Sometimes it becomes problem for him to urinate…and the blood was transfused..so the child weight shooted to 8kgs..

Que: What symptoms made you feel the most need to visit the health facility?

Res: Body weight of his child, it reduces. "Child playing " that is what made me come at this facility.

Que: For How long did you have the symptoms before visiting the facility?

Res: For many months, as I told you earlier it started on June now we are at December, almost 5 Months now.

Que: What made you wait before seeking for treatment?

Res: Have been taking to dispensary but no disease detected apart from Malaria, also I was treating him with herbal medicine and tattooing the swelling part of the stomach.

Que: Did you seek alternative source of treatment before coming to the facility?

Res: "mmm" I was using Herbal products.

Que:What challenges did you experienced?

Res:I get all problems

Que: Which kind of problems?

Res: My homestead left with nobody, I lack food here "child cough"

Que: What factor motivated you to seek help outside of your household for your illness?

Res: I seek treatment at this facility because it is accessible.

Que: This is the only hospital around here?

Res: They are many but I choose this because it the biggest Hospital at the place with treatment equipment.

Que: What measures if any helped you during process of seeking care?

Res: I'm here alone, so nobody helped me.

Que: Among your Household, who decides on whether to seek or not to seek care when persons gets sick? "Child talking "

Res: The father of the child, who decided for me to take this child to the hospital..

Que: So you can't decide yourself to take your child at Hospital when he is sick?

Res: We talk as a family how we could treat him. But the father is the final man because He is the one who to decide which animal we use for treatment expenses.

Que: Were you aware you could get diagnosis and treatment for Kalazar in this facility before you child fell ill?

Res: I was aware the disease is treated here at this facility "Child playing "

Que: Where did you get the information?(sounds from playing birds)

Res: I get the information from people who were treated at this facility.

Que: Did they treated successfully?

Res: Yes, they were treated well.

Que: Where do your community members seek help for the condition your are suffering from?

Res: They seek help at this facility.

Que: Please, tell me your experience on the health care you are receiving.

Res: Good reception from the health workers, The child is fairing better now and still undergoing with medicine. Food to feed him is the only problem I had.

Que: Do doctors follow up the health status after treatment?

Res: They Don't follow at home, it is me who came here for his body check up as we get back home.

Que: What kind of support are you receiving from family and friends?

Res: Since I came at this facility, no person come and visit us.

Que: So, no support you are receiving at any member of the family?

Res: No support.

Que:even your huband?

Res:my husband stayed back home to look for other things at home..

Que:How much does it cost you as a Kalazar patient in terms of personal expenses?

Res: I spent Kshs 500.

Que: What is this 500 shillings for?

Res: They used for blood screening at Laboratory.

Que: In considering the steps you took, what do you think you would do differently now if you could start from the beginning?

Res: If I could have taken him to the Hospital earlier, He could have done with his injection and get better.

Que: What intervention would you suggest to improve VL care and access to VL care.

Res: General cleanness, and improving our structures to prevent dust.

Que: "mmmmh "

Res:"eeeh"

Que: So according to you, it is dirty environment that causes Kalazar?

Res: "Mmh " it is poor hygiene and uncooked food."Child coughing "

.

Que: If any of your friend or relative develop VL, What would you recommend

.mto them in terms of treatment? "Child cough "

Res:,,,,,Will inform the sick person to seek treatment at 4re of any interventions for VL in the county.

Res: Not aware. There was company of World vision that teaches people about Hygiene and sanitation of toilets.

Que: Kindly give more information about the barriers to access VL diagnosis care and treatment.

Res: Was confused by Turkana beliefs and traditions of using Herbal Medicine for treatment.

Que: Please tell me What type of people have the greatest challenge accessing VL treatment and why?

Res: Nowadays everybody reach at facility when felt his or her body is not functioning normally. "Child talking "

Que:Why women are mostly at Hospital?

Res: Youth mostly beliefs that, when they take those facility medicine, they bring other diseases to his body.

Que:What are measures you feel should be put in place to address the barriers and improve access to VL services?

Res:The Government should bring VL medicine to nearby facilities, Donate Food..for support to our bodies..

Que: What do the community members say about the condition you are suffering from?

Res: It is a bad disease."Child talking " they advice me to seek medication at Hospital.

Que: What is the impact of community perception on VL care and diagnosis?

Res: Family people take it as normal disease. Maybe the other people of which

nothing I can tell them.

Que: What can be done at the community level to reduce stigma?

Res: By teaching people about the disease.

Que: What is the best way to involve the community in strategies to combat and control VL?

Res: By talking to them.

Que: How would we reach them?

Res: By visiting house to house, some occasion they organize meetings to people about the disease…thank you for participation……

Time: 38 Minutes 55 Seconds.
