# Supplementary material for: Barriers and facilitators of care among visceral leishmaniasis patients following the implementation of a decentralized model in Turkana County, Kenya
Source: PLOS Glob Public Health. 2025 Mar 31;5(3):e0004161. doi: 10.1371/journal.pgph.0004161 (PMC11957299; doi:10.1371/journal.pgph.0004161)
Supplement: S1 Data — This file includes the following transcripts: •VL Patient In-depth Interview Transcripts: Verbatim transcripts of interviews conducted with VL patients, capturing their insights and lived experiences. •Healthcare Worker Key Informant Interview (KII) Transcripts: Transcripts from key informant interviews with healthcare workers, detailing their perspectives on decentralized care models for VL. (ZIP) [file pgph.0004161.s003.zip › HCW and IDI transcripts/patient interviews/Res 013_FACILITY 4.docx]

VL DECENTRALISED STUDY

VL PATIENT/CAREGIVER INDEPTH INTERVIEW

**PATIENT INTERVIEW**
QUE 1:How many days have your child been admitted at this facility?
RES : Half of the last month .16days today."eeh"

QUE 2:Tell me about the condition your son is suffering from?
RES: They said he is sick of kala Azar.

QUE 3:What do you think causes the disease your child is suffering from?
RES:I don't know what causes it ,if it's the Satan or God that sents it to a human body  'eeh' ("power generator sound and birds chirping')

QUE 4: Briefly describe some of the symptoms experienced by a person with kala Azar
RES: something drains the blood in someone's body,it finishes until he loose weight and becomes weak and sleepy,eyes become swollen and a protruding abdomen.

QUE: What else?

RES: Fever at night and a scaly skin.That is why he is lying down in one side.

QUE 5:From where did you learn about the condition your son is suffering from?
RES:This disease has come offlate it wasn't there.The Turkanas were just living like that.It has come nowadays and it's rampant In the world.

QUE: Where did do you get the information from?

RES: Have never heard about this disease but I came to know if it when my son got it.When I brought him to the government's hand and was diagnosed of kala Azar.That is  where I heard about kala Azar.("babies cry".)

QUE: The doctor tells you?

RES: The doctor was the one who tested. I was curious what he was suffering from and when I brought to the governments hand ,the government said it is kala Azar.It was here in the hospital where he was tested with no knowledge of what kind of disease is this.

QUE 6:Is there any member of your household or community member you are aware or has suffered a similar disease?
RES:No one knew at all.("child playing in the background)

QUE 7:Do you think this condition is a problem within the village you come from?
RES:yes it a big problem ,I don't know if it's God or Satan brings this disease like this.

QUE 8: Compared to Malaria and other conditions,how would you describe VL burden in your area?
RES:Kala Azar is a very bad disease . It's deadly when it comes.It finishes somebody completely.Malaria is treatable  "aahh" and after treatment somebody comes back to life but this one damages and that's what it's visible in him suffering.

QUE 9:Whom do you think is most at risk of getting kala Azar?
RES:Maybe it's the governments that should know and help the Turkana people Because us the illiterate does not know.

QUE: Which category of individual is risk of getting Kalazar?

RES: There's no one I can say can contact kala Azar".my husband is old and am also there to there's no way I should know.

QUE: Which places are prone to this disease?
RES: It's most prevalent in rural areas. Reserve there and it's also in town. I don't know why it's cases are high.

QUE: Which periods of time are most prevalence to the people?
RES: When reaches the rainy season mostly end of year that's when kala Azar become rampant.

QUE 10:Tell me more about kala Azar and how it's spread?
RES:When this disease begin to strike somebody's body becomes hairy,eyes become yellow,get sickly in the morning and nights that's when you will know that this person has become ill of a certain disease.And when it's the morning sleeps and even the mostly affordable foods like maize he declines to eat.

QUE: How do think it transmit?
RES: In my thinking I don't know how it's spread .I assume that it's God who sends it and place it on someone's belly.Provided that there is no even saying it was transmitted from maybe a cut .And so we say maybe it's being sent to someone.("baby cries)

QUE 11:What do you think you can do to protect yourself and your child from the disease?
RES:There's nothing I can do.'Myself or my child,I wouldn't say I can protect from this disease.God is the one who puts it inside a person's body "eeeeh"This small kid I don't know where he gets the disease from.

QUE 12: Briefly tell me how the disease is diagnosed.
RES:"like I told you,i saw him become sick and when I brought him to the doctor's hands,that's when he took the blood from the veins and said it was kala Azar.

QUE 13: Briefly tell me how the disease is treated
RES:When I bring him he is given an injection to the leg,an then you were told to go.He is injected two injections one on the hand and the other on the thigh every morning.('children crying and birds chirping'.)

QUE 14:When did you first become aware that your child is ill?
RES:My son became anaemic one month ago.It continued to finish his blood and so when the stomach became big is when I wondered ''oooooh'' what was this. He came to the “government’s hand”(mkono ya serikali) at last.,,,,,,,,,,,,,”mmm”

QUE 15: What are some of the symptoms you experienced before coming to the facility?
RES: He had yellow eyes,he had a protruding abdomen and lost appetite.

QUE 16:What symptom made you feel the most need to visit the health facility?
RES:He slept flat like someone who was about to die.I just took him saying that this thing has killed him.""eeh "and brought him .I saw his skin become hairy and looked bad. He was just sleeping throughout.

QUE 17: For how long did you have the symptoms before visiting the facility?
RES: He had the symptoms for one month.

QUE 18:What made you wait for those 30 days before seeking treatment?
RES:I never knew that he is sick of kala Azar ,,she became swayed just like that by when he was still strong.I never saw him sleeping or down.When I noticed he has started to sleep too much is when I decided to bring him to the hospital.""mmmh""

QUE 19:Did you seek any alternative source of treatment before coming to the facility?
RES:I never went to look for treatment somewhere else only Lopiding Sub -County Hospital.

QUE: Did you seek traditional medicine?

RES: There was nothing I did to him even when he had fevers.It was when I came here and asked the doctors to examine him so as to know the reason why he had fevers.

QUE: Did you used any other medicine?
RES: I never used any medication………..

QUE 20:What are the challenges you face as a parent to a kala Azar child?
RES:I have a problem with his feeding food and that after the doctor gives him the drug he became weak and sleeps ,,After waking up he feels like eating and I have nothing to give him.I  am  told to give him good food  but I don't find any and so he sleeps hungry that night.The same applies the same morning ,I come to the hospital.He is getting a problem because when the treatment commenced ,the only remaining issue is what to put in his mouth so that his blood will be back to normal.I really don't have anything to offer to him.

QUE: Is anyone of your household suffering from Kalazar?
RES: It's only this person is the only burden in my family.The others are just okay.I see that this disease is a dangerous and can lead you to death.

QUE 21: What factors motivated you to seek help outside of your household for your son's illness?
RES: What motivated me is when they started administering medication to my son and I even saw him sit and open his eyes they way you see.Nothing else helped me.The doctors medication is helping him.

QUE 22:What measures helped you during your process of care seeking?
RES: Nothing else helped me apart form the drugs administered by the doctor.

QUE 23:Among your household,who decides on whether to seek or not seek care when a person gets sick?
RES: My husband is the decision maker.So when someone gets sick he informs me on his in ability to help in other way but you should go to the government's hand (mkono ya serikali) because they are the ones who can help. Even when they is a problem with drought,the government is the one to help. He cannot decide because there is nothing he can do.'Just go to the government.They say the government has eyes and it's the only one that helps people.

QUE 24:Were you aware you could get diagnosis and treatment for kala Azar in this facility before your son become ill?
RES:God is the one who showed me of this place.That I knew I would get life in the governments hands.I came just like that and said to myself that when you have problems the only place you can run to is the doctors hands.And the doctor examines you.

QUE: Was you there his Kalazar drugs here or not?

RES: I never knew that this facility treat kala Azar.But God made treatment available.

QUE 25:Where do your community members seek help for the condition your son is suffering from?
RES: They come to this same hospital.They don't go anywhere only Lopiding Sub -County hospital.

QUE: How the community say about the disease?

RES: They say it's a bad disease and if you can't go to the hospital you .It's more  dangerous than other diseases.

QUE 26: please tell me of your experience on the healthcare you are receiving
RES:The drugs administration is okay.my Son is not the way he was.He was very sick but when I brought him to the government's hand and gave him drugs ,,he has regained strength.

QUE: When the child blood transfused

RES: They took blood sample at 7am in the morning and at 8am the result were already there.

QUE: They were given treatment after the results?
RES: He was introduced to treatment the same time…….”mmmm”
When I got the medication as compared to when I came with him when he was almost dead.The few days he has been in medication he has improved.
The drugs are given through injection 'eeeh'there are no oral drugs.Only that when the drugs become much the hand become swollen of injections and it's given through this other hand.There is nothing bad I see.

QUE: How will you suggest on drugs folloe up after treatment?
RES: I suggest if the government follow up on patient it is good. But if they can give some aid that will help the patient back home to regain strength.
That's why I say if the government is good they can help with that.So that when they say this person has been discharged there is something for them to eat back at home to be healthy again.

QUE 27:What kind of support are you receiving from family and friends to help cope with the hospital visits and kala Azar treatment?
RES: There's no support am getting.No one,no one has ever visited me at home,no not even one person.Me and this boy we just sleep at home ,his body has not improved yet.He his just hungry.He lack what to eat good food.I chance on getting meals.

QUE 28:How much does it cost you as a parent to a kala Azar child,in terms of personal expenses.
RES:I have not used any cash .I had only ksh 500 that I got from charcoal .That's the only amount I used to pay for diagnosis.There was no money to pay for other services.Even medication I don't know if the government will pay I will lack money to pay.You also see that I am poor.

QUE 29:In considering the steps you took,what do you think you would do differently now if you could start from the beginning?
RES: There's nothing I was willing to do but God showed me eyes to seek for treatment.That is why am just in the doctors hands.If my son would be fine in the hand of government, The Government is the one who will help.In terms of medication and food for my child.

QUE 30:What changes or interventions would you suggest to improve VL care and access to VL care ?
RES:The government should treat its people and those that are needy like my son to be helped by giving aid to them.
Even this drugs should be given for free because I don't have money to pay for the governments drugs.The problem with this person is just lack of food,he is getting his medication well but because there is nothing in the stomach.The drugs are very strong as said by the government.They say that you need to eat well at home .So for me I don't have anything to give him .That's what I want to tell the government.The government should conduct diagnosis.

QUE 31:If any of your friends or relatives develop VL,what would you recommend to them in terms of treatment?
RES:If my neighbors got kala Azar I will tell him/her to go to the hospital because that's where help is.In the village if you decide to stay you just die.The government will help with treatment and nutritional support.

QUE 32:Are you aware of any past interventions for VL in the county?
RES:I have never but I also want to know the truth through my sick child because it's good to know the truth and what the government haa done.Then you will say that the county helped me in this way or the other.In terms of treatment and food.

QUE 33:Kindly give more information about the barriers to access of VL diagnosis,care and treatment.
RES:It's a challenge when the person has not gotten diagnosed, but all this is done and introduced to  treatment by the doctors,he regains strength.
The long waiting hours before being injected,due to the other patients.So you are told to wait For other patients to finish.


QUE 34: What type of people have the greatest challenge accessing VL treatment and why?
RES: The county is the one who should get aid and help kala Azar patients. Men and women and also children when they get Kalaazar die if they don't get treated.
Old men and women got affected by this disease when they get it." child interruptions"
When a old man or woman get this disease it's very bad to them "eeeh"...

QUE: Why the old people?
RES: Because Kala Azar comes first starting with the protruding abdomen. Their age makes them not access treatment early.


QUE 35:What are the measures you feel should be put in place to address the barriers and improve access to VL services?
RES: I say that if the county people are willing to help the kala Azar patients, They should offer some nutritional support,if it's the drug they should help too....'eeeh'
They should increase supply of drugs to the the hospital so that they will get there each Time they become Ill.
The county should also look for aids to help them gain strength when on treatment. Because that drug can also destroy the body either for old men or others. When you take medication without proper nutrition.

QUE 36:What can you tell me about the risks of developing VL once a person leave Turkana county?
RES: yes he/she can contact the disease if he drinks dirty water from the river. Especially water, he/she can get kala Azar .

QUE: Was you aware of this disease?

RES: When I was growing up this disease was not there but when I become old all things have come ,Like there's a kala Azar person 'ooh' liver and many others .I don't know what has brought all that maybe it's the devil .


QUE 37:What do community members say about kala Azar that your child is suffering from?
RES: The community people say that this disease has come and it's bad.If you can't go to the hospital you die.
They believe that you should go to the hospital to get treated.

QUE 38:What is the impact of community perceptions on VL care and diagnosis?
RES:Their sayings prompts someone to go to the hospital and be diagnosed.They say if you see your child having fevers you should go to the doctor to determine whether it's kala Azar or malaria…….”Birds noise”


QUE 39:What can be done at the community level to reduce stigma?
RES:The county people should visit the villages and train that you should not stay with a kid when he/she becomes sick. You should take the child to the hospital to be tested to know whether it's kala Azar or malaria. The government people should train people.

QUE 40:What is the best way to involve the community in strategies to combat and control VL?
RES:The government should get a solution and know that the kala Azar disease is becoming rampant.They should wake up and help the illiterates from the rural areas because they are dying of kala Azar .
They should find either drugs to treat them.
They should come together to help the people.

QUE: Is there anything you would like to ask me?
Res: What I can say is that having medication without proper nutrition gives me stress, and that is why am heading to being weak. That is what I would tell you.
